# Stereoselective *E*‑Carbofunctionalization of Alkynes to Vinyl-Triflates *via* Gold Redox Catalysis

**DOI:** 10.1021/acsorginorgau.5c00084

**Published:** 2025-09-24

**Authors:** Filippo Campagnolo, Lorenza Armando, Elisa Boccalon, Alessandra Cicolella, Manfred Bochmann, Giovanni Talarico, Luca Rocchigiani

**Affiliations:** † Department of Chemistry, Biology and Biotechnology, University of Perugia and CIRCC, Via Elce di Sotto 8, 06123 Perugia, Italy; ‡ Scuola Superiore Meridionale, Largo San Marcellino, 80138 Naples, Italy; § School of Chemistry, Pharmacy and Pharmacology, 6106 University of East Anglia, Norwich Research Park, NR4 7TJ Norwich, U.K.; ∥ Department of Chemical Sciences, 9316 University of Naples Federico II, Via Cintia, 80126 Naples, Italy

**Keywords:** gold catalysis, vinyl-triflates, stereoselectivity, 1,2-carbofunctionalization, DFT studies

## Abstract

Carbofunctionalization of alkynes with trifluoromethylsulfonate nucleophiles is a powerful strategy for the synthesis of vinyl triflates with diverse molecular complexity. However, stereoselective protocols are challenging to realize, and the development of novel strategies for controlling the selectivity is highly desirable. In this work, we show that gold complexes bearing the hemilabile MeDalPhos ligand (MeDalPhos = di­(1-adamantyl)-2-dimethylamino-phenylphosphine) catalyze the *E*-stereoselective carbofunctionalization of internal alkynes using aryl/vinyl iodides and AgOTf as simple starting reagents. Based on the outer-sphere nature of this reaction and the beneficial effect of the MeDalPhos ligand, the *Z*-selective attack is practically suppressed, leading to an ideal kinetic selectivity. Mechanistic studies, both experimental and theoretical, revealed that the interplay between kinetics and thermodynamics is crucial in determining the final *E*/*Z* ratios for each substrate.

## Graphical abstract

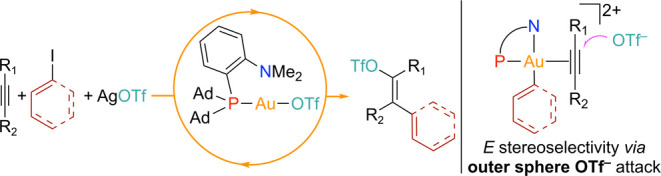

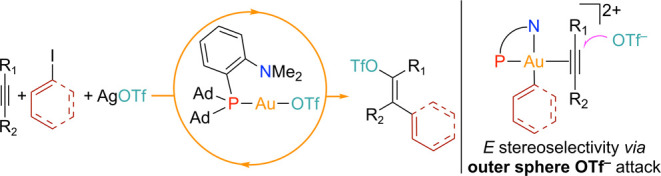

## Introduction

Organic triflates R–OTf (OTf^–^ = CF_3_SO_3_^–^) are useful reagents that find diverse applications in synthetic chemistry. The remarkable nucleofugal properties of the OTf^–^ anion, indeed, make the cleavage of C–OTf bonds easy under nucleophilic substitution or solvolysis conditions and enable a wide range of derivatization reactions.^1,2^ Within this family of compounds, vinyl triflates are of particular interest and demonstrated great potential in carbocationic chemistry.^3–5^ Moreover, they are key substrates in transition metal-catalyzed cross-coupling reactions^6–11^ owing to their facile oxidative addition to low-valent metal centers, which leads to cationic intermediates where the stereochemistry of the double bond is retained.^12,13^

Vinyl triflates are typically prepared upon trifluoromethanesulfonation of enolates with triflic anhydride or amides (Scheme 1a).^14^ However, synthetic methods involving alkynes are becoming increasingly interesting due to the broad availability and structural diversity of these substrates.^15^ In this respect, triflation of C≡C bonds with TfOH or TfOTMS reagents has attracted attention and various stoichiometric/catalytic protocols have been recently developed (Scheme 1b).^16–18^

**Scheme 1:**
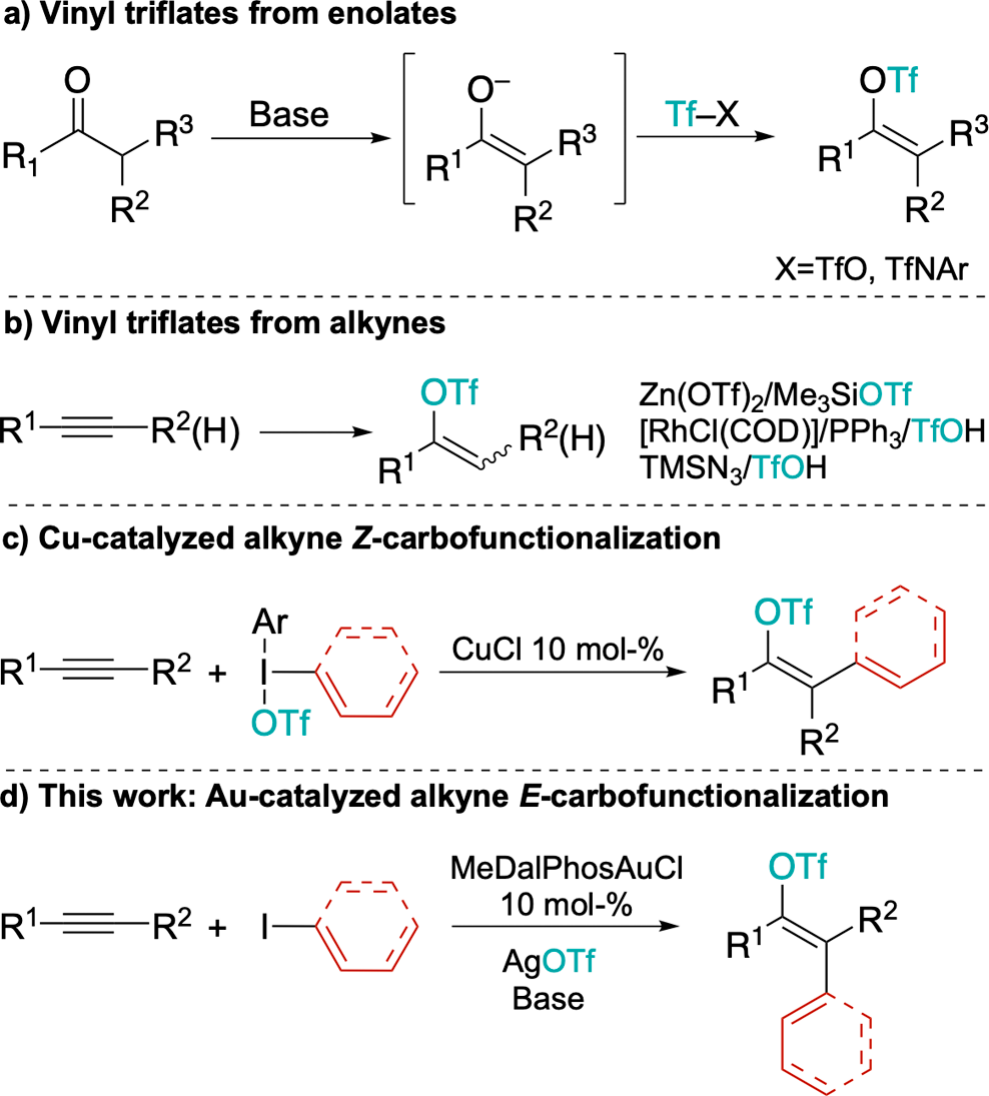
Synthesis of Vinyl Triflates and Synopsis of This Work

These reactions, though, inevitably afford vinyl triflates having a β-hydrogen atom on the double bond. Therefore, a few 1,2-carbofunctionalization protocols have been developed to access more complex vinyl triflate skeletons.^19^

Notable work by Gaunt^20^ and Liu^21^ showed that terminal and internal alkynes are selectively converted to trisubstituted vinyl triflates by a Cu-catalyzed carbofunctionalization reaction that exploits iodonium derivatives as both oxidants for Cu­(I) and OTf sources (Scheme 1c). Isolated vinyl triflates^20^ have preferentially *Z* configurations, likely arising from an inner sphere C–OTf reductive elimination. Similarly, Wang and Studer reported the formation of perfluoroalkyl vinyl triflates from iodonium reagents and alkynes under radical conditions, affording *E* vinyl triflates.^22^ Despite the intense research in the field, carbofunctionalization protocols affording *E* vinyl triflates from aryl iodides, alkynes, and simple triflate sources are not commonplace and would be highly desirable.

During the past decade, oxidant-free redox gold catalysis^23–25^ emerged as a useful tool for the selective 1,2 carbofunctionalization of alkenes^26–28^ and alkynes.^29^ Reasonably, this reactivity may be exploited in combination with OTf^–^ nucleophiles to generate vinyl triflates.^30^ In particular, gold catalysts bearing the hemilabile MeDalPhos ligand (MeDalPhos = di­(1-adamantyl)-2-dimethylamino-phenylphosphine) are appealing as they easily undergo oxidative addition of iodoarenes,^31–34^ forming cationic Au­(III) intermediates that are able to activate unsaturated substrates toward nucleophilic attack.

Usually, OTf^–^ counterions are assumed to be innocent during catalysis so that AgOTf is often used as a halide abstractor during reactions involving hemilabile gold catalysts.^28,32,35^ Nevertheless, we recently observed that the MeDalPhosAuCl complex mediates the formation of alkyl triflates when activated with AgOTf in the presence of iodoarenes and α-olefins.^36,37^ This observation was key to demonstrate the unique character of gold and its complementarity to Pd in the context of the catalytic Heck coupling reactivity.^38–41^

We reasoned that using alkynes instead of alkenes would afford vinyl triflates starting from readily available substrates like ArI and AgOTf (Scheme 1d).^42^ According to the earlier mechanistic proposals for the alkene reactions,^36,43,44^ the electrophilic carbofunctionalization step occurs *via* an outer sphere mechanism. In the present case, this would enable *E* selectivity upon nucleophilic attack of the OTf^–^ anion onto coordinated alkyne.^45,46^

In the present work, we explore the feasibility of such reactions, from both the stoichiometric and catalytic points of view, rationally testing the scope of substrates to probe for the generality of the reactions and dissect the reaction mechanism and its limitations.

## Results and Discussion

### Stoichiometric Experiments and Catalysis Optimization

The viability of our mechanistic hypothesis was first tested with stoichiometric reactions. The previously reported^36^ gold­(III) [(P^N)­Au­(Ar)­(OTf)]­[OTf] ion pair **1OMe** (P^N = MeDalPhos, Ar = 4-methoxyphenyl) was generated *in situ* upon reacting the Au­(I) chloride precursor with AgOTf and *p*-iodoanisole (2.5 and 1.1 equiv, respectively) and successively mixed with 2.5 equiv of 3-hexyne, which was chosen as a model substrate. After 12 h at 298 K, the ^1^H NMR spectrum of the reaction mixture showed quantitative consumption of **1OMe** to give the reduced [(P^N)­Au­(3-hexyne)]­[OTf][Bibr ref47] salt **3** and a single organic derivative that was assigned to *E*-4-methoxyphenylhex-3-enyl trifluoromethanesulfonate (**2a**, Figures 1 and S1, Supporting Information) based on 1D- and 2D-NMR experiments. In particular, ^19^F ^1^H HOESY NMR experiments allowed us to confirm the *E* configuration of the product based on the presence of selective dipolar interactions between the CF_3_ moiety and both the CH_2_ groups of the ethyl chains (Figure S2, Supporting Information).

**Figure 1:**
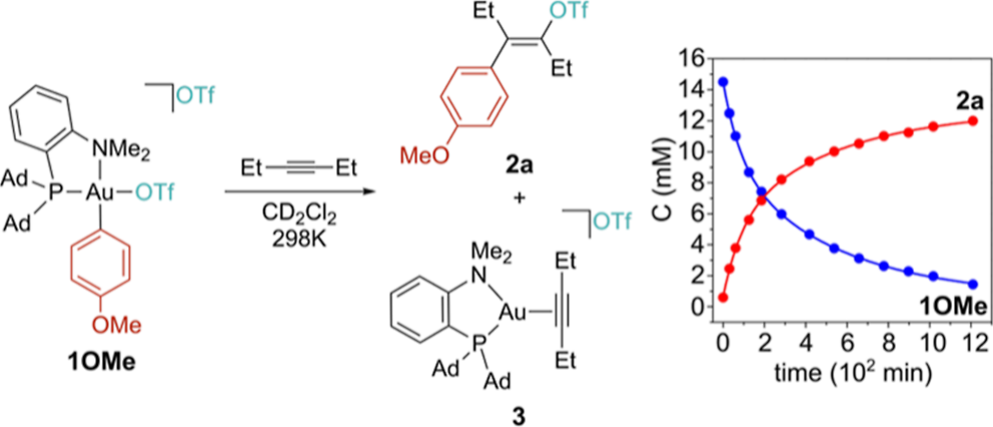
Reaction between **1OMe** and 2.5 equiv of 3-hexyne (CD_2_Cl_2_, 298 K) and the corresponding kinetic profile.

Upon following the reaction as a function of time by ^1^H NMR (Figure 1), it can be seen that **2a** and **3** form directly from **1OMe** without the accumulation of any intermediate, in agreement to what we observed already with α-olefins.^36,37^ While the formation to **2a** is quantitative, **3** forms in only about 65% yield owing to the partial conversion to its N-protonated form^36,48^ (see the Supporting Information). The Au­(I) resting state is stable in the presence of **2a** for days in solution at room temperature, suggesting that DalPhos-type Au­(I) complexes do not undergo easy oxidative addition of vinyl triflates.

Gratifyingly, the reaction proceeds also under catalytic conditions when MeDalPhosAuCl (10 mol %) was mixed in CD_2_Cl_2_ with 3-hexyne, *p*-iodoanisole, and 1.1 equiv of AgOTf with respect to the substrates. Under these unoptimized conditions, 28% conversion was achieved after 24 h at room temperature (38% at 48 h). Analysis of the crude by ^1^H NMR spectroscopy suggested that low conversions are due to complete protonation of the Au­(I) resting state, possibly owing to the presence of adventitious water contained in the silver salt. Amine protonation prevents further oxidative addition of the Ar–I bond and stops the catalytic cycle. 2,6-Lutidine (0.4 equiv to the substrates) was therefore added as an external base to avoid catalyst deactivation. Optimal conditions were found using 1,2-dichloroethane (DCE) as reaction medium at 60 °C, achieving over 95% conversion after 16 h (Table 1). Such conditions proved effective also for a scale up of the reaction to 1 mmol scale, without any loss of conversion/selectivity.

**Table 1:**
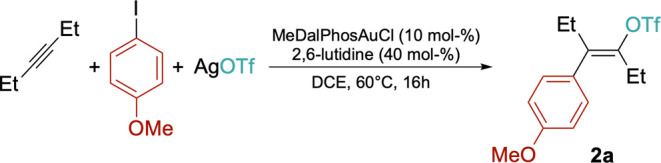
Optimization of Catalytic Conditions^a^

entry	deviation from standard conditions	yield (**2a**)^b^ (%)
1	no LAuCl, no 2,6-lutidine, 80 °C, 48 h	
2	no LAuCl, 2,6-lutidine 0.3 equiv, 80 °C, 48 h	
3	no 2,6-lutidine, DCM, 50 °C, 48 h	38
4	DCM, 50 °C, 48 h	89
5	80 °C	>95
6	none	>95

a Standard conditions: *p*-iodoanisole 0.05 mmol; 3-hexyne 0.05 mmol; AgOTf 0.08 mmol; 2,6-lutidine 0.02 mmol; DCE 500 μL.

b Determined by ^1^H NMR.

Control experiments indicated that the reaction retains a high selectivity for **2a** up to reaction temperatures of 80 °C (*E*/*Z* = 95:5) and does not take place in the absence of gold, with or without added base (Table 1). This rules out the occurrence of acid-catalyzed pathways triggered by the degradation of DCE by AgOTf.^49^

### Scope of the Reaction

The scope of the reaction was first tested by changing the alkyne, keeping *p*-iodoanisole and AgOTf as substrates working under the standard conditions. First, the reaction is not fully regioselective in the case of asymmetric alkynes such as 2-hexyne, for which two regioisomers **2b** and **2b′** were observed in a 52:48 ratio (>95% conversion, Scheme 2). The latter increases to 70:30 by lowering the temperature to 50 °C and using DCM as solvent, even though the conversion is slightly reduced to 90% after 48 h. In both cases, high *E* stereoselectivity (>97%) was observed.

**Scheme 2:**
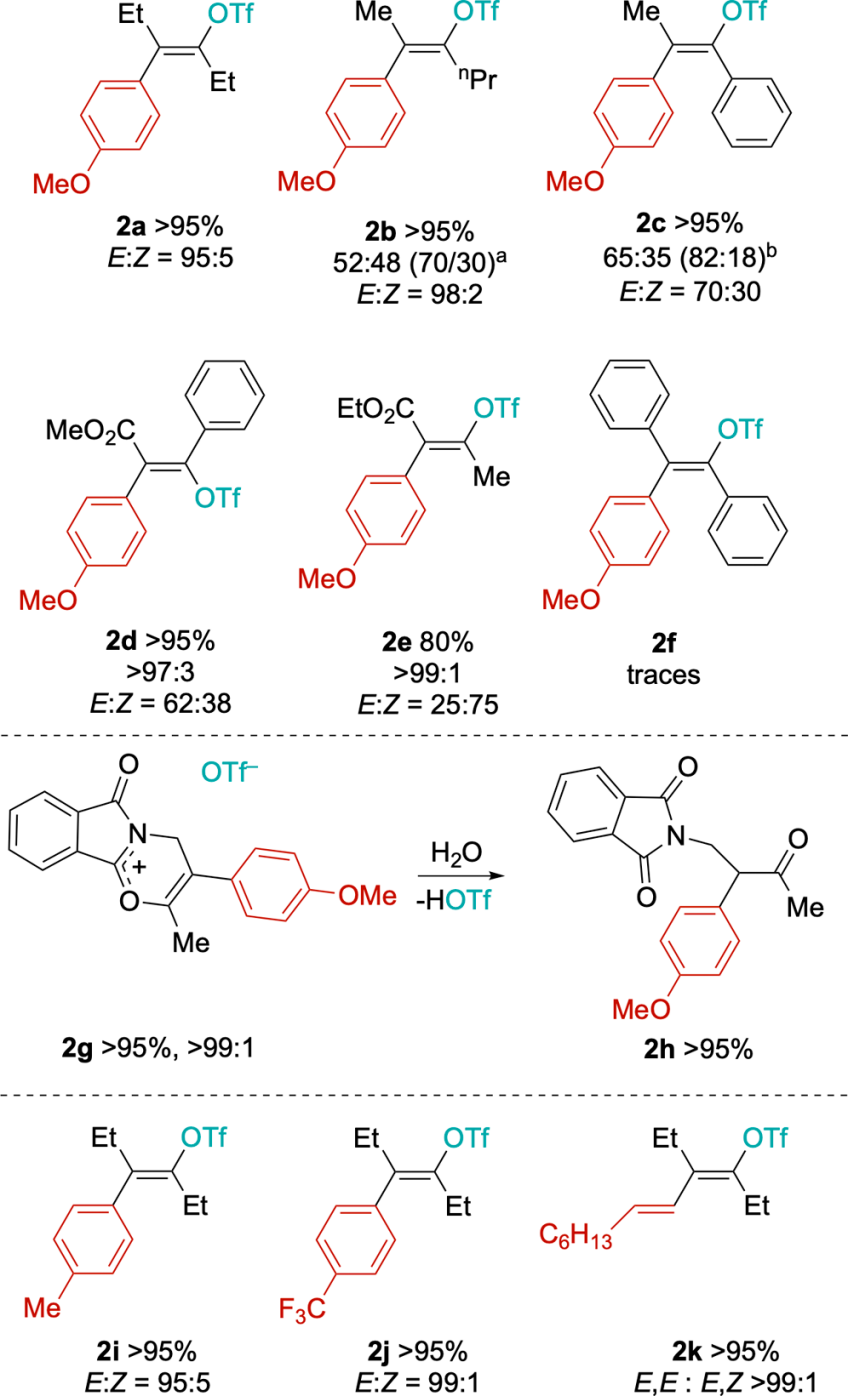
General Scope of Alkyne Carbofunctionalization^a^

a Major isomer is shown along regioisomeric ratios for vinyl triflates affording multiple isomers (^a^DCM, 50 °C, 48 h; ^b^DCE, 25 °C, 48 h, conversions by ^1^H NMR).

In the case of 1-phenyl-1-propyne, both regio- and stereoselectivity are reduced and the major product **2c** (OTf in α position with respect to the Ph ring) is obtained in the mixture with all the other 3 possible isomers, in an overall **2c**/**2c′** regioisomeric ratio of 65:35 (*E*/*Z* = 70:30). An improved regioselectivity (***E*-2c** = 82%) is obtained on lowering the temperature to 25 °C and running the reaction for 48 h (Supporting Information). Ester-based methyl phenylpropiolate and ethyl 2-butinoate can also be converted into the corresponding vinyl triflates **2d** and **2e** in high yields and regioselectivity.^50^ While **2e** is obtained as the expected major *Z* stereoisomer (*E*/*Z* = 25:75), **2d** is mostly present as the *E* isomer (*E*/*Z* = 62:38), where the *p*-methoxyphenyl and the OTf groups are in the *syn* configuration.

Bulky alkynes are more difficult to convert. Diphenylacetylene, for example, can only be converted to vinyl triflate **2f** in low yield (<10%) and only using high temperatures. Other alkynes such as 4,4-dimethylpentyne, di-*tert*-butylacetylene bis carboxylate, or bis-1-adamantylacetylene did not react at all, suggesting that the steric bulk of the substrate is a main limitation for the scope of this catalytic reaction (see mechanistic studies).

A notably different result was obtained with *N-*(2-butynyl)­phthalimide, which does not afford the typical vinyl triflate but rather undergoes a heterocyclization reaction, leading to salt **2g** in high yield and selectivity. The latter spontaneously hydrolyzed to the corresponding ketone **2h** during chromatographic purification. No conversion was observed with terminal alkynes such as phenylacetylene or halogenated 2-bromo-1-pentyne.

The scope of the iodide was explored by using 3-hexyne and AgOTf as substrates under standard conditions. Replacing *p*-iodoanisole with *p*-iodotoluene does not affect the reactivity and vinyl triflate **2i** (Scheme 2) is obtained with the same yield and selectivity observed for **2b**. Similarly, *p*-iodo-trifluoromethylbenzene affords **2j** in excellent *E* selectivity (*E*/*Z* 99:1). The protocol can be extended to iodoolefins as well, as exemplified with *trans*-1-iodoctene, which affords the corresponding dienyl triflate **2k** in good yields. There is an optimal selectivity for the *E*,*E* isomer, suggesting that the stereochemistry of the 1-iodoolefin is retained upon the oxidative addition/reductive elimination sequence.^51^

Steric bulk is also important in limiting the iodoarene scope. As an example, 1-iodonaphthalene was found to be completely unreactive as well as the electron-poor iodo-pentafluorobenzene. This seems to indicate that the catalytic reaction is sensitive to minor changes in the alkyne/iodoarene structure. To pinpoint the important factors that limit conversion and selectivity, mechanistic experiments have been performed by in situ NMR spectroscopy.

### Mechanistic Studies

Looking at the scope of the reaction, it appears that alkynes bearing alkyl substituents lead to high *E* selectivity, while *E*/*Z* ratios are reduced when aromatic substrates are employed. To have a grasp of the factors that affect the selectivity, we carried out a stoichiometric experiment by reacting MeDalPhosAuCl, AgOTf, *p*-iodoanisole, and 2-phenylpropyne in the absence of base and monitored selectivity as a function of time (Table 2 and Figures S3 and S4).

**Table 2:**
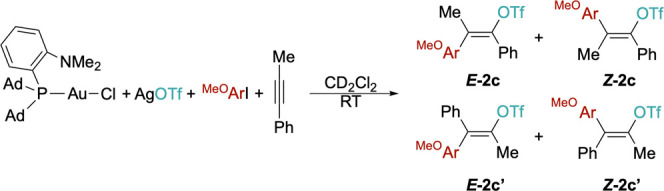
Selectivity Monitoring in Stochiometric Reactions^a^

time (min)	% conversion	**E-2c**/**Z-2c**/**E-2c′**/**Z-2c′**	**E-2c**/**Z-2c**
20	5	86:5:9:0	94:6
40	10	86:5:9:0	94:6
240	50	84:5:10:1	94:6
420	66	82:7:10:1	92:8
1320	95	77:11:10:2	87:13

a 0.01 mmol LAuCl, 0.025 mmol AgOTf, 0.01 mmol *p*-iodoanisole, 0.015 mmol 3-hexyne.

The results indicate that, under these conditions, the reaction is almost quantitative after 22 h (298 K, CD_2_Cl_2_) and affords an *E*/*Z* ratio of 87:13 and a **2c**/**2c′** ratio of 88:12, both increased with respect to what was observed in the catalytic reaction under standard conditions.

Notably, the stereoselectivity decreases with increasing conversion, while regioselectivity is mostly unaffected by the reaction time. This can be explained by considering that regioselectivity is dictated by the energy difference between the two transition states leading to 1,2 and 2,1 attacks, which is constant at the same temperature. On the other hand, the decreasing stereoselectivity for both **2c** and **2c′** can be instead explained assuming the occurrence of a slow double bond isomerization, possibly acid-catalyzed, after the generation of the product, which is enabled by the steric pressure of the two aromatic rings.^52^ This is in line with the observation that catalytic runs performed at lower temperatures show increased selectivity (Scheme 2).

*In situ* NMR investigations were performed also to shed some light on the observed scope of iodoarene substrates. First, naphthyl derivative **1naph** was generated upon fast oxidative addition of 1-iodonaphthalene in the presence of 2.5 equiv of AgOTf. In comparison with **1OMe**, anion metathesis seems to be slightly more difficult for the naphthyl derivative and 90% conversion is observed after prolonged heating at 55 °C, despite using a molar excess of AgOTf (Figure 2). The ^19^F NMR spectrum of **1napth** showed the presence of two different signals at δ_F_ = −77.7 and 78.2 ppm, which were assigned to coordinated and outer-sphere triflate anions.^53^ The observation of two separate resonances is indicative of a reduced rate of triflate exchange for this species compared with **1OMe**, which may be related to a lower lability of the metal–anion interactions. ^19^F NOE NMR indicated, however, that such exchange is not frozen on the NMR time scale and leads to extensive chemical exchange saturating the NOESY spectrum (τ_M_ = 100 ms, Figure 2b). Based on line broadening,^54^ the exchange rate can be roughly estimated as 125 s^–1^, affording a limiting Δ*G*_298K_^≠^ value of 14.5 kcal/mol. This value is considerably larger than that of **1OMe** (10.2 kcal/mol) and indicates that replacing an anisole with a naphthyl group hampers anion exchange.

**Figure 2:**
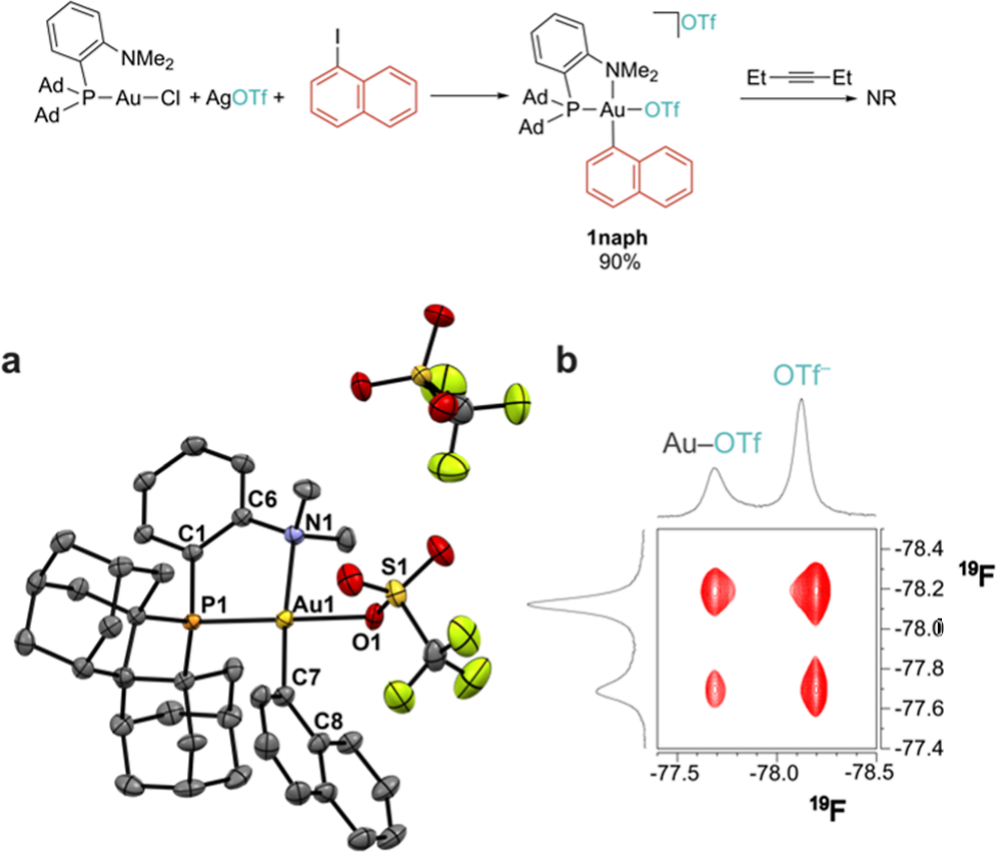
Top: synthesis of **1naph**. Bottom: (a) crystal structure of **1naph** (thermal ellipsoids are shown at 50% probability, hydrogen atoms and solvent molecules are omitted for clarity); selected bond distances (Å) and angles (°): Au1–P1 2.290(1), Au1–N1 2.175(3), Au1–C7 2.054(4), Au1–O1 2.129(4), O1–S1 1.457(3), Au1–O1–S1 124.8(2), P1–Au1–N1 86.67(9), P1–Au1–O1 173.30(9), O1–Au1–C7–C8 63.8°. (b) ^19^F NOESY NMR spectrum (CD_2_Cl_2_, 298 K, τ_m_ = 100 ms) showing extensive chemical exchange between coordinated and outer-sphere OTf^–^ anions.

To get molecular insights into this effect, we obtained single crystals of the Au­(III) derivative that were analyzed by X-ray diffraction. The molecular structure of **1naph** (Figure 2a) is quite similar to that of **1OMe**,^36^ featuring the typical square planar Au­(III) configuration with metal–ligand distances in the range expected for this class of compounds (Au1–P1 2.290(1), Au1–N1 2.175(3), Au–C7 2.054(4) Å). The gold-triflate distance is only marginally shorter (Au1–O1 2.129(4) for **1naph** vs 2.143(2) Å for **1OMe**), while the Au1–O1–S1 angle amounts to 124.8(2)° (120.6(1)° for **1OMe**). The naphthyl group is tilted with respect to the (P^N)Au plane with an O1–Au1–C7–C8 dihedral of 63.8°. Due to the steric pressure of the NMe_2_ group, the triflate anion orients its CF_3_ moiety in front of the naphthyl ring (C8–F2 short distance of 3.149 Å).

Despite the strong structural similarity between **1naph** and **1OMe**, the former species proved to be unreactive toward 3-hexyne over the period of 2 days at room temperature. This suggests that the lack of catalytic conversion arises from a difficult coordination of the alkyne to the Au center. Most likely, this is not related to a stronger metal–anion interaction but rather to an increased steric pressure of the naphthyl ring that may complicate the substrate distortion required for the nucleophilic attack.

A similar result was obtained in the case of IC_6_F_5_, which undergoes oxidative addition affording the corresponding Au­(III) iodide perfluoroaryl complex.^31^ In this case, ^31^P NMR studies indicated that halide exchange is not quantitative and a mixture of **1C**_**6**_ **F**_**5**_ (δ_P_ = 124.1 ppm) and its chloride analogue (δ_P_ = 102.0 ppm) form in solution after warming up at 55 °C for 2 h, despite using a 2.5 molar excess of AgOTf. Using a larger excess of silver salts leads to unidentified side products that precipitate from the solution. As in the case of **1naph**, **1C**_**6**_ **F**_**5**_ shows two different OTf resonances in the ^19^F NMR spectrum (broad singlet at δ_F_ = −77.7 and sharp singlet at δ_F_ = −78.7 ppm), suggesting that triflate exchange is slow in the NMR time scale (see Supporting Information). These observations seem to suggest that the pentafluorophenyl ring increases the Lewis acidity of the gold center, which would reduce the anion dissociation tendency. In agreement with the latter, **1C**_**6**_ **F**_**5**_ is unreactive toward 3-hexyne and no conversion to vinyl triflate was observed over 24 h at room temperature.

*Trans*-1-iodo-octene shows a behavior similar to that of *para*-iodoanisole as the quantitative formation of **1C**_**8**_ **H**_**15**_ was observed upon reacting the starting gold­(I) chloride with 2.5 equiv of AgOTf and 1.5 equiv of iodide. The ^1^H NOESY spectrum of **1C**_**8**_ **H**_**15**_ indicates that the vinyl retains the trans configuration while the ^19^F NMR spectrum shows the presence of only a single OTf signal (δ_F_ = −77.9 ppm), indicative of fast anion exchange at RT. The addition of 1.5 equiv of 3 hexyne to **1C**_**8**_ **H**_**15**_ leads to the quantitative formation of **2k** over the period of 4 h at RT (Figure 3).

**Figure 3:**
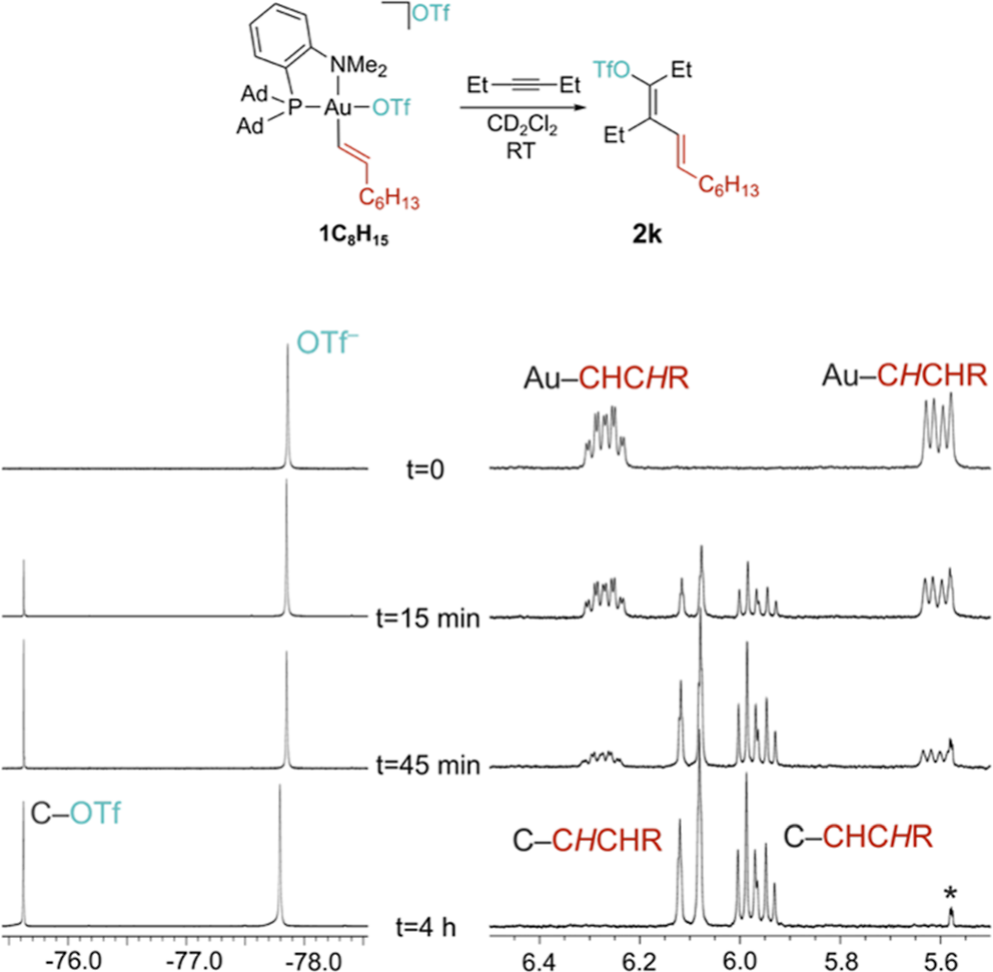
Reactivity of **1C**_**8**_ **H**_**15**_ with 3-hexyne and evolution of the ^19^F NMR (left) and a section of the ^1^H NMR spectrum (right) after the addition of 1.5 equiv of alkyne (CD_2_Cl_2_, 298 K); the asterisk denotes a CHDCl_2_ sideband.

Summarizing, mechanistic studies underline the importance of steric factors in this reaction. Bulky alkynes do not coordinate to the Au­(III) center after oxidative addition, so that the Au–OTf bond is not broken and no nucleophilic attack takes place. Similarly, bulky iodoarenes seem to prevent the coordination of any alkyne, leading to no conversion. To gain a better understanding of these phenomena, we performed a DFT investigation of the most important intermediates and reactivities.

### DFT Calculations

Details of DFT calculations are reported in the Supporting Information. Figure 4a shows the Gibbs energy profile for the reaction pathway with 3-hexyne, starting from intermediate **INT0** formed after the oxidative addition of *p*-iodoanisole and continuing to the release of final product **2a**. The competitive reaction pathways leading to the *E* (**prod-E**, black line) and *Z* (**prod-Z**, gray line) isomers were evaluated.

**Figure 4:**
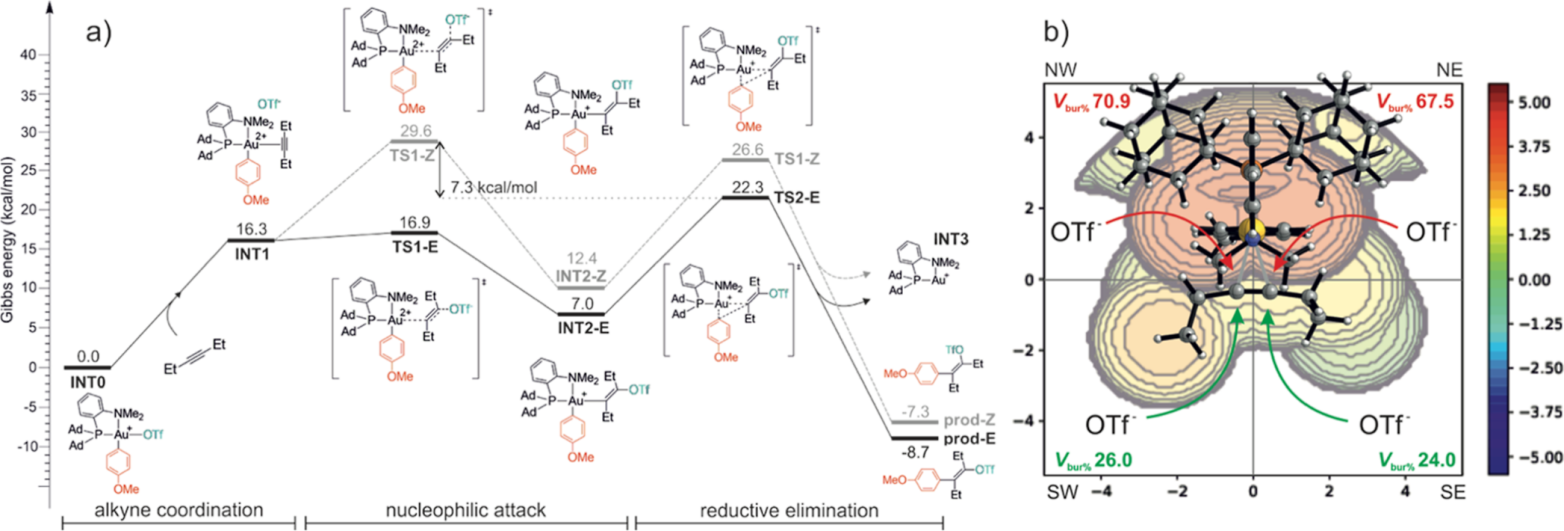
(a) Gibbs energy profile (kcal/mol) of the carbofunctionalization reaction leading to product **2a**; (b) steric map with the percentage of buried volume (% *V*_bur_) of **INT1**. The OTf^–^ anion was excluded from the computation of the % *V*_bur_ for clarity.

Upon coordination to the metal center (**INT1**), the alkyne is oriented perpendicularly with respect to the (P^N)Au plane and undergoes nucleophilic attack by the triflate anion to form the *E*-configured product *via* transition state **TS1-E**, with an activation barrier of 16.9 kcal/mol. By contrast, the formation of the *Z* product *via* **TS1-Z** is energetically less favorable, requiring an additional 12.7 kcal/mol with respect to **TS1-E** (Figure 4a). The origin of such a large energy difference might be easily visualized through the analysis of the steric map of **INT1** reported in Figure 4b. The formation of the *E* isomer involves attack of OTf^–^ from the less hindered region of the map (SW and SE quadrants in Figure 4b). The *Z* nucleophilic attack necessitates instead a less favorable approach of the alkyne from the more sterically congested sides (NW and NE quadrants in Figure 4b). Moreover, the steric interactions between the anion and the ligand framework impose a significant distortion in the Au–C­(sp)–C­(sp) bond angle in **TS1-Z** (112.3°), compared to **TS1-E** (86.7°), as better illustrated in Figure S47A,B (Supporting Information).

For completing the reaction paths, we modeled the reductive elimination leading to the *E* and *Z* products. The Gibbs energetic values for such steps (**TS2-E** and **TS2-Z** in Figure 4a) are closer, with respect to the nucleophilic attack analogous steps. **TS2-Z** (26.6 kcal/mol) is indeed 4 kcal/mol higher in energy than **TS2-E** (22.3 kcal/mol), and in both TSs, the Au–Csp–Csp bond angles are approximately 110° (Figure S47C,D, Supporting Information). The Au–N bond lengths increase from 2.25 Å in **INT2** to 2.43 Å, suggesting that elongation of the Au–N bond is required to facilitate the reductive elimination. Interestingly, DFT calculations indicate a switching in the rate-determining step (RDS) for the reaction, with the nucleophilic addition (**TS1-Z**) for *Z*-selective and the reductive elimination (**TS2-E**) for *E*-selective pathways, respectively. Such a feature is confirmed also by computing the complete energetic profile paths of 2-butyne (Figure S48, Supporting Information).

The theoretical estimation of stereoselectivity, based just on the energy difference between the RDS of the *E* and *Z* pathways, is extremely high (approximately 7 kcal/mol). It remains high also by computing the energetics in the presence of additional OTf^–^ anions (Figure S49, Supporting Information). However, the experimentally observed 95:5 *E*/*Z* ratio is accurately accounted for by considering the relative thermodynamic stabilities of the *E* and *Z* products (ΔΔ*G* of **prod-E** and **prod-Z** = 1.4 kcal/mol, Figure 4a), also taking into account potential acid-catalyzed isomerization processes.^55^

These findings indicate that thermodynamic and kinetic parameters must be considered for stereoselectivity of the reaction. Both factors concur to explain the observed lack of reactivity also in the case of bulky alkynes such as di-*tert*-butylacetylene. The calculated reaction path is reported in Figure S50 (Supporting Information). The computational analysis revealed a general increase in both the activation barrier (57.3 kcal/mol in the most favorable case) combined with unfavorable thermodynamics (Figure S50, Supporting Information). The employment of such a hindered alkyne leads to an increased distortion of the system in **TS2-E-** ^***t***^ **Bu** with respect to **TS2-E** (Figures S51A vs S47C, Supporting Information), and in unfavorable interactions occurring between the *tert*-butyl groups and −NMe_2_, aryl, and adamantyl moieties, as evidenced in Figure S51B (Supporting Information).

## Conclusions

In conclusion, we have provided a convenient route to a range of vinyl triflates by exploiting an *E*-stereoselective, ligand-enabled gold-catalyzed 1,2 alkyne carbofunctionalization with aryl/vinyl iodides and AgOTf.

Mechanistic studies provided insights on the catalytic cycle (Scheme 3), which matches with our previous findings on α-olefins carbofunctionalization^36,37^ and consists in a sequence of oxidative addition (a), anion metathesis (b), nucleophilic attack by OTf^–^ to a coordinated alkyne (c), and reductive elimination of the product (d). The DalPhos ligand system here plays a doubly beneficial role as it facilitates oxidative addition^31^ and imparts a notable stereoselectivity to the nucleophilic attack step. The degradation of the *E*/*Z* ratio that is observed with some substrates is likely arising from isomerization reactions that occur after the catalytic reaction. We also elucidated some of the factors that may limit the substrate scope, such as steric encumbrance and electronics. The most critical step appears to be alkyne coordination/nucleophilic attack, which becomes particularly unfavorable with bulky alkynes/aryl iodides.

**Scheme 3:**
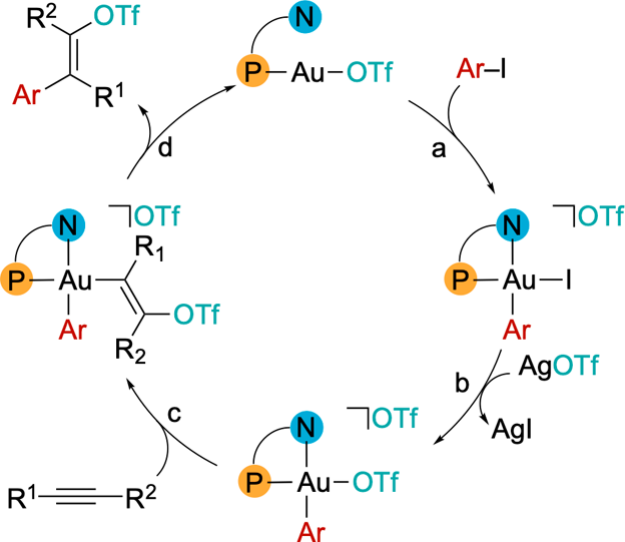
Mechanism of Gold-Catalyzed Alkyne *E*-Carbofunctionalization to Vinyl Triflates

The capability of [(P^N)­Au­(Ar)­X]­[OTf] complexes to trigger nucleophilic attack of a good leaving group like the OTf^–^ anion is an intriguing strategy that may open to a series of multistep catalytic reactions in which organic triflates are generated as transient intermediates.^56^ It is anticipated that this strategy will be extended to other unsaturated substrates to understand the broad implications of this reactivity.

## Experimental Section

All manipulations were performed under exclusion of moisture and oxygen in flame-dried Schlenk glassware on a Schlenk line or in a nitrogen-filled MBraun Unilab glovebox with high-capacity recirculation (<1 ppm of O_2_ and H_2_O). Chloro­[di­(1-adamantyl)-2-dimethylaminophenylphosphine]­gold­(I) (MeDalPhosAuCl) was purchased from Merck or synthesized following literature procedures from commercially available di­(1-adamantyl)-2-dimethylaminophenylphosphine (Merck) and dried under high-vacuum conditions before use. Substrates 3-hexyne, 2-hexyne, 2-phenylpropyne, methyl phenylpropiolate, ethyl 2-butinoate, diphenylacetylene, *N*(2-butynyl)­phthalimide, phenylacetylene, 2-bromo-1-pentyne, *p*-iodotoluene, 1-iodonaphthalene, iodopentafluorobenzene, *p*-iodotoluene, *p*-trifluoromethyl-iodobenzene, and *trans*-1-iodoctene (Merck) were dried under high vacuum and stored in the glovebox. Liquid reagents were stored under activated 4 Å molecular sieves. AgOTf (Merck) was used as received and stored in the glovebox. Solvents for synthesis and chromatography were dried over the appropriate drying agent, freeze–pump–thawed, and distilled before use. CD_2_Cl_2_ (Eurisotop) was freeze–pump–thaw degassed, distilled over CaH_2_, and stored over 4 Å molecular sieves in the glovebox.

^1^H, ^1^H PGSE, ^19^F, ^31^P­{^1^H}, ^13^C­{^1^H}, ^1^H NOESY, ^19^F NOESY, ^1^H, ^13^C HMQC, ^1^H, ^13^C HSQC, ^1^H, ^13^C HMBC, and ^19^F ^1^H HOESY NMR experiments have been recorded on a Bruker Avance III HD 400 spectrometer equipped with ^1^H, BB smartprobe (400.13 MHz for ^1^H) and Z-gradients or on a Bruker Avance NEO 600 spectrometer equipped with a Prodigy Bruker Cryoprobe (600.13 MHz for ^1^H) with a *z* gradient coil. ^1^H NMR spectra are referenced to residual protons of the deuterated solvent. ^13^C NMR spectra are referenced to the D-coupled ^13^C signals of the solvent. ^19^F NMR spectra are referenced to an external standard of CFCl_3_. ^31^P NMR spectra are referenced to an external standard of H_3_PO_4_.

### General Procedure for Stoichiometric Reactions

A 4 mL vial equipped with a 5 mm magnetic stirring bar was charged with MeDalPhosAuCl (7.8 mg, 0.012 mmol, 1.0 equiv), AgOTf (10.1 mg, 0.048 mmol, 4.0 equiv), and the iodoarene substrate (0.012 mmol, 1.0 equiv). CD_2_Cl_2_ (500 μL) and 2,6-lutidine (0.5 μL, 0.004 mmol, 0.4 equiv) were then added to afford a heterogeneous mixture. The resulting mixture was transferred to a 5 mm J. Young NMR tube for analysis. When required, the alkyne (0.012 mmol, 1.0 equiv) was subsequently added, and the reaction mixture was further analyzed by NMR spectroscopy. For reactions requiring heating, the J Young NMR tubes were placed inside an oil bath at 55 °C.

### General Catalytic Procedure

A 4 mL vial equipped with a 5 mm magnetic stirring bar was charged with MeDalPhosAuCl (3.0 mg, 0.005 mmol, 0.1 equiv), AgOTf (15.3 mg, 0.060 mmol, 1.3 equiv), and the iodoarene substrate (0.050 mmol, 1.0 equiv). Dichloroethane (DCE, 500 μL) and 2,6-lutidine (2.2 μL, 0.018 mmol, 0.4 equiv) were then added to afford a heterogeneous mixture. The alkyne (0.050 mmol, 1.0 equiv) was subsequently added, and the resulting reaction mixture was stirred at 60 °C for 8–16 h within a heating block in the glovebox. After completion of the reaction, the solvent was removed under reduced pressure, and the residue was dissolved in CD_2_Cl_2_ under an inert atmosphere for analysis by ^1^H NMR spectroscopy. Liquid reactants were measured by using a microsyringe. Solid alkynes were preweighed in a separate vial, into which the initial reaction mixture was transferred. Yields and characterization data for the vinyl triflates are given in the Supporting Information.

### General Procedure for Vinyl Triflate Isolation

The reaction mixture was concentrated under reduced pressure and dissolved in a minimum amount of the eluent. The resulting solution was loaded onto a prepacked silica gel flash chromatography column and purified to afford the product as a colorless oil. Products sensitive to degradation were instead extracted with dry, degassed pentane and isolated by solvent removal under reduced pressure. Complete conditions and solvents are specified in Table S2 (see the Supporting Information).

### Upscaled Synthesis of **2a**

A dry, degassed Schlenk tube equipped with a magnetic stirring bar was loaded with MeDalPhosAuCl (65.6 mg, 0.1 mmol, 0.1 equiv), AgOTf (282.0 mg, 1.1 mmol, 1.1 equiv), and *para*-iodoanisole (234.0 mg, 1.0 mmol, 1 equiv). Successively, DCE (1.5 mL) and 2,6 lutidine (46.6 μL, 0.4 mmol, 0.4 equiv) were added to obtain a heterogeneous mixture. 3-Hexyne (115.2 μL, 1.0 mmol, 1.0 equiv) was injected by using a micrometric syringe, and the mixture was stirred at 60 °C for 16 h in an oil bath. At the end of the reaction, all the volatiles were removed under reduced pressure and pentane was added affording an off-white suspension. The supernatant solution was passed through a prepacked silica gel flash chromatography column, and the resulting solution was dried under vacuum affording **2a** as a colorless oil. Yield: 289.3 mg (85%).

## Supplementary Material



## Data Availability

The data underlying this study are available in the published article and its Supporting Information.

## Notes

*In situ* NMR experiments, NMR characterization data, crystallographic data, and details of the DFT calculations (PDF)
