# Supplementary material for: Stereoselective E‑Carbofunctionalization of Alkynes to Vinyl-Triflates via Gold Redox Catalysis
Source: ACS Org Inorg Au. 2025 Sep 24;5(6):548–56. doi: 10.1021/acsorginorgau.5c00084 (PMC12679304; doi:10.1021/acsorginorgau.5c00084)
Supplement: Supplementary file 1 [file gg5c00084_si_001.pdf]

SUPPORTING INFORMATION

Stereoselective *E*-carbofunctionalization of alkynes to vinyl-triflates *via* gold redox catalysis

Filippo Campagnolo,[a] Lorenza Armando,[a] Elisa Boccalon,[a] Alessandra Cicolella,[b] Manfred Bochmann,[c] Giovanni Talarico,[b,d,*] and Luca Rocchigiani[a,*]

[a] Department of Chemistry, Biology and Biotechnology, University of Perugia and CIRCC, via Elce di Sotto 8, 06123, Perugia, Italy.
E-mail: luca.rocchigiani@unipg.it

[b] Scuola Superiore Meridionale, Largo San Marcellino, 80138, Naples, Italy

[c] School of Chemistry, Pharmacy and Pharmacology, University of East Anglia, Norwich Research Park, NR4 7TJ, Norwich, UK.

[d] Department of Chemical Sciences, University of Naples Federico II, Via Cintia, 80126, Naples, Italy.
E-mail: talarico@unina.it

CONTENTS:

1. **Additional spectra for *in situ* NMR reactions**
2. **Characterization of organic products**
3. **X ray crystallography**
4. **DFT details and additional computational results**

1. Additional spectra for *in situ* NMR reactions

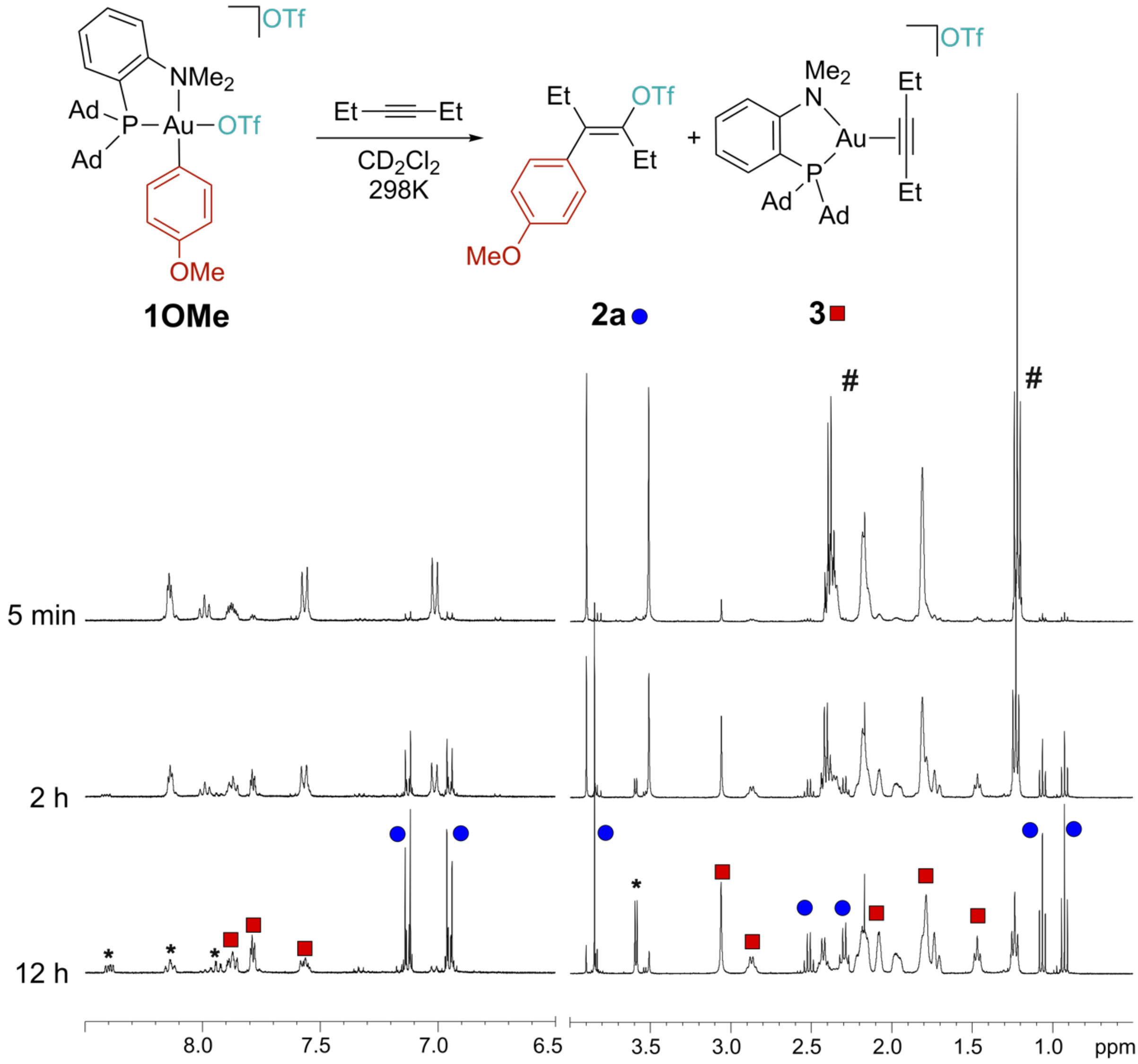

Figure S.1 Evolution of the ^{1}H NMR spectrum of **1OMe** after the addition of 2.5 equiv of 3-hexyne (298K, CD_2Cl_2); asterisks denote protonated [(P-NH)AuX] salt, hashtag denote 3-hexyne.

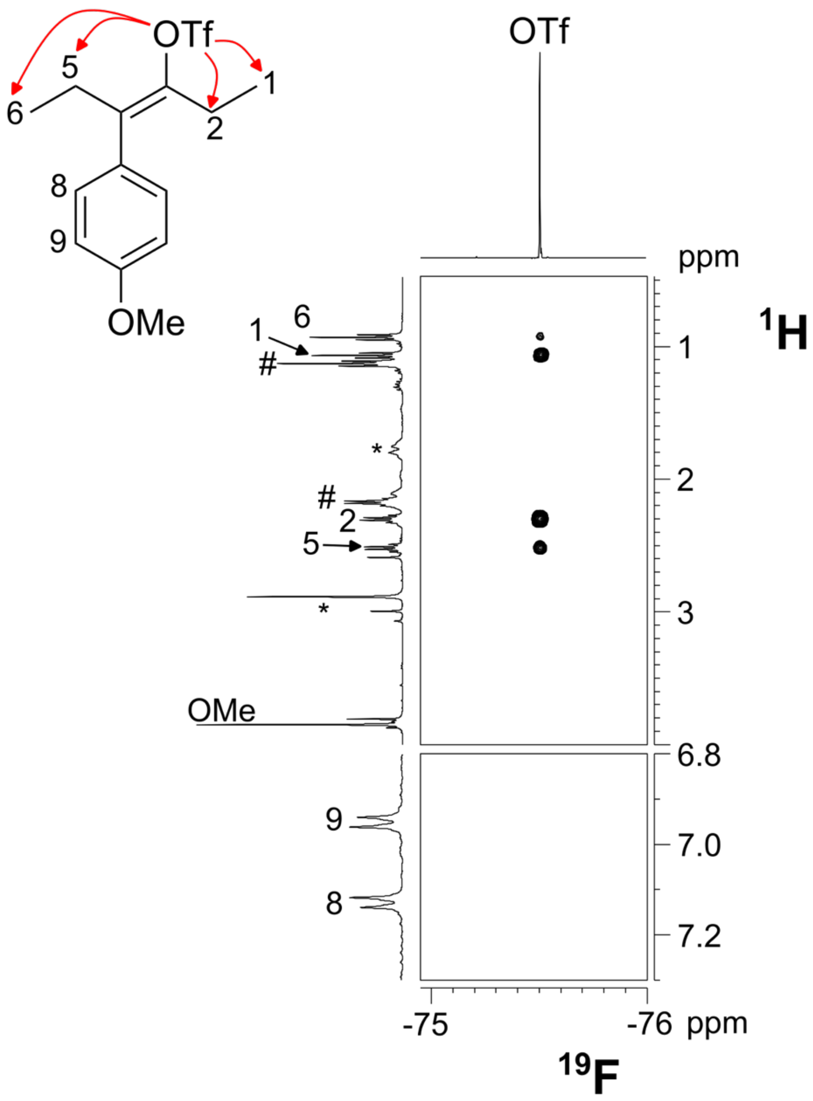

Figure S.2 Two sections of the ^{19}F ^{1}H HOESY NMR spectrum of **2a** (CD_2Cl_2, RT) highlighting specific NOE interactions confirming *E* stereochemistry. Asterisks denote residual gold complexes, hashtags denote 3-hexyne.

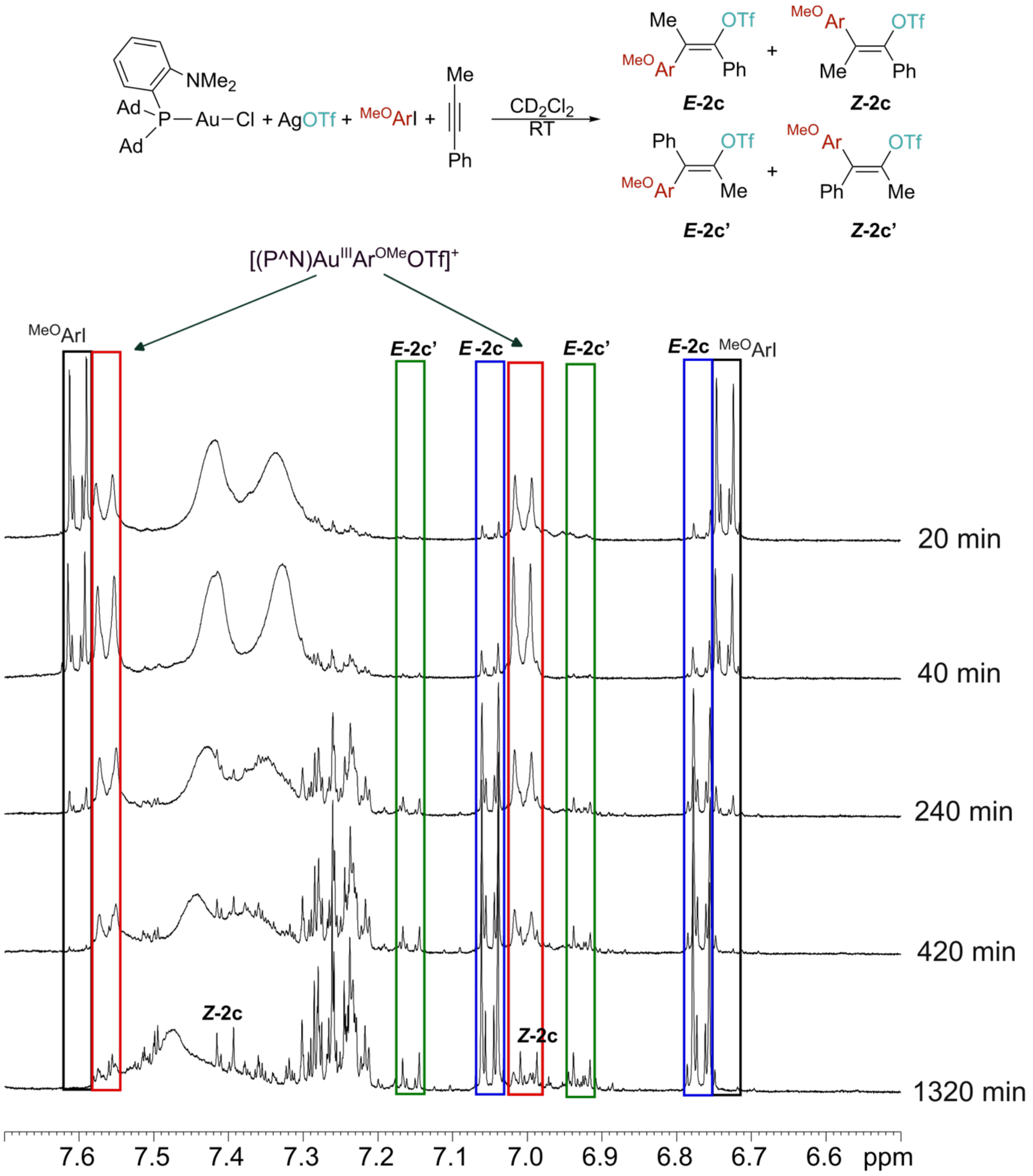

Figure S.3 Time evolution of a section of the ^1H NMR spectrum of a mixture containing MeDalPhosAuCl (0.01 mmol), AgOTf (0.025 mmol), *p*-iodoanisole (0.01 mmol) and 1-phenyl-1-propyne (0.015 mmol) in CD_2Cl_2 at 298 K.

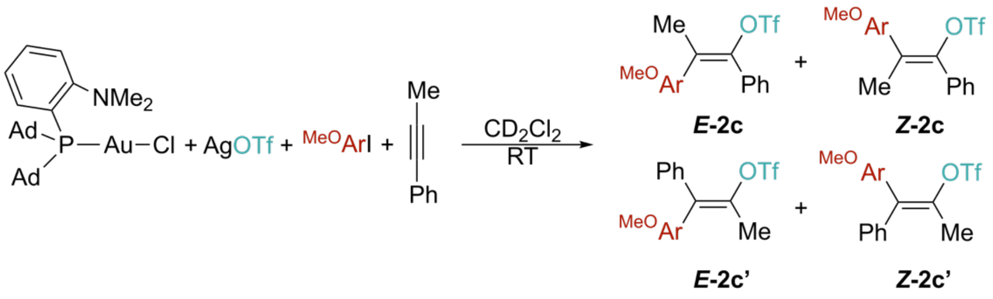

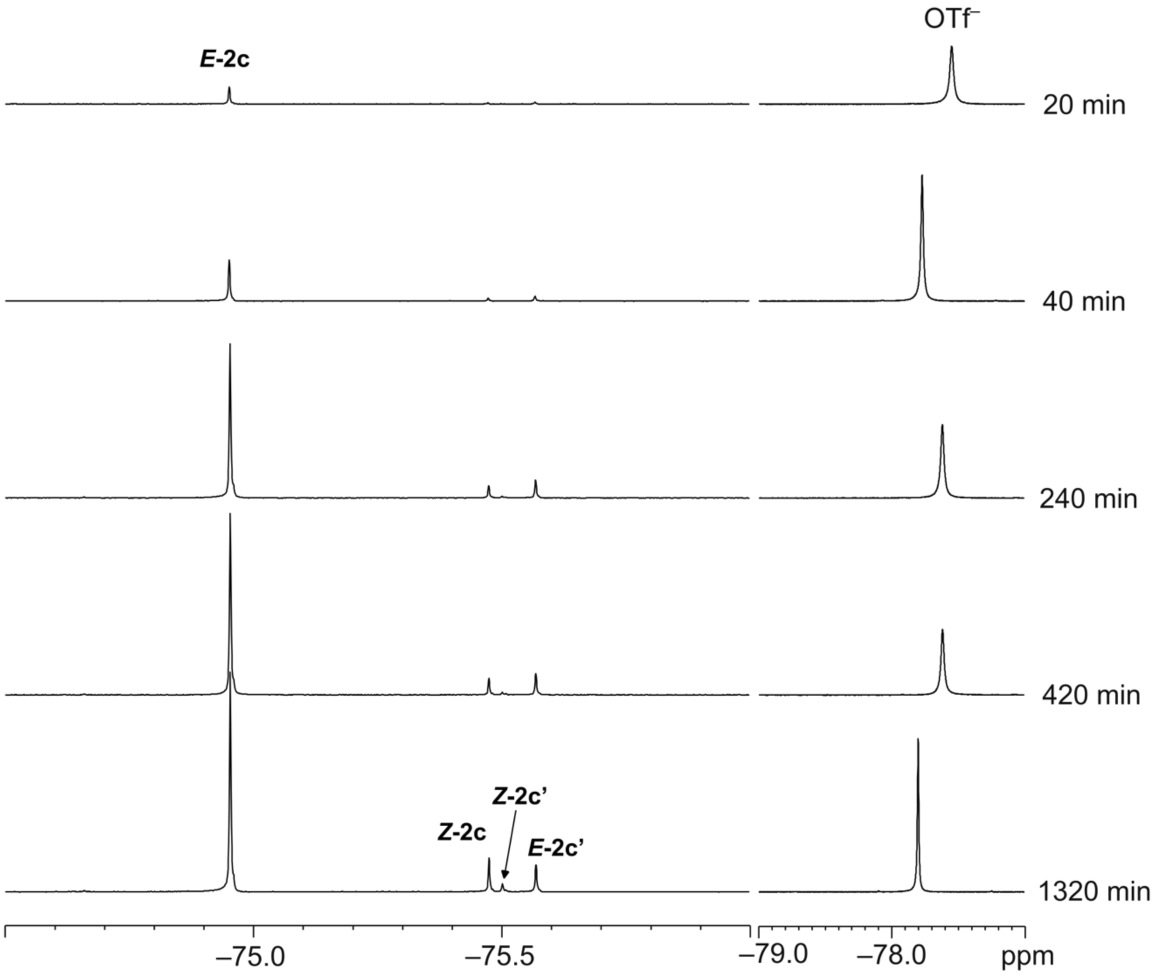

Figure S.4 Time evolution of two sections of the ^{19}F NMR spectrum of a mixture containing MeDalPhosAuCl (0.01 mmol), AgOTf (0.025 mmol), *p*-iodoanisole (0.01 mmol) and 1-phenyl-1-propyne (0.015 mmol) in CD_2Cl_2 at 298 K.

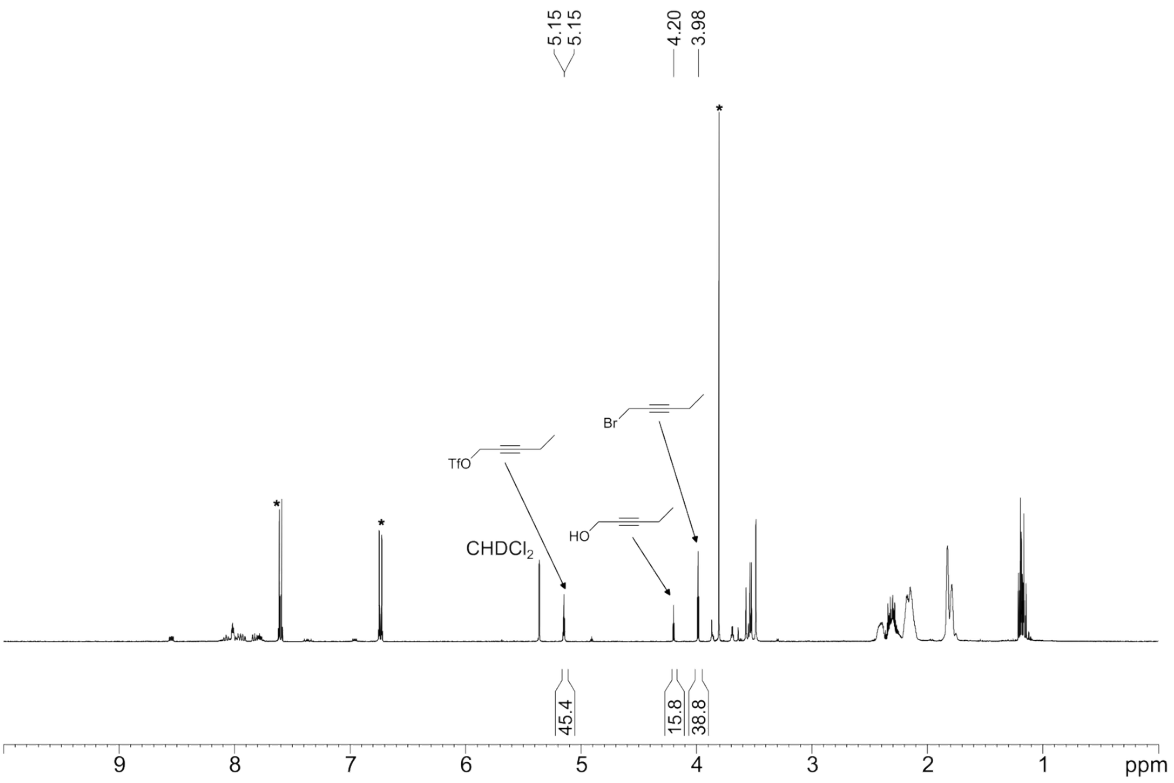

Figure S.5 ^{1}H NMR spectrum obtained after mixing MeDalPhosAuCl (0.01 mmol), AgOTf (0.025 mmol), *p*-iodoanisole (0.01 mmol) and 1-Br-2-pentyne (0.015 mmol) in CD_2Cl_2 at 298 K; asterisks denote unreacted iodoanisole.

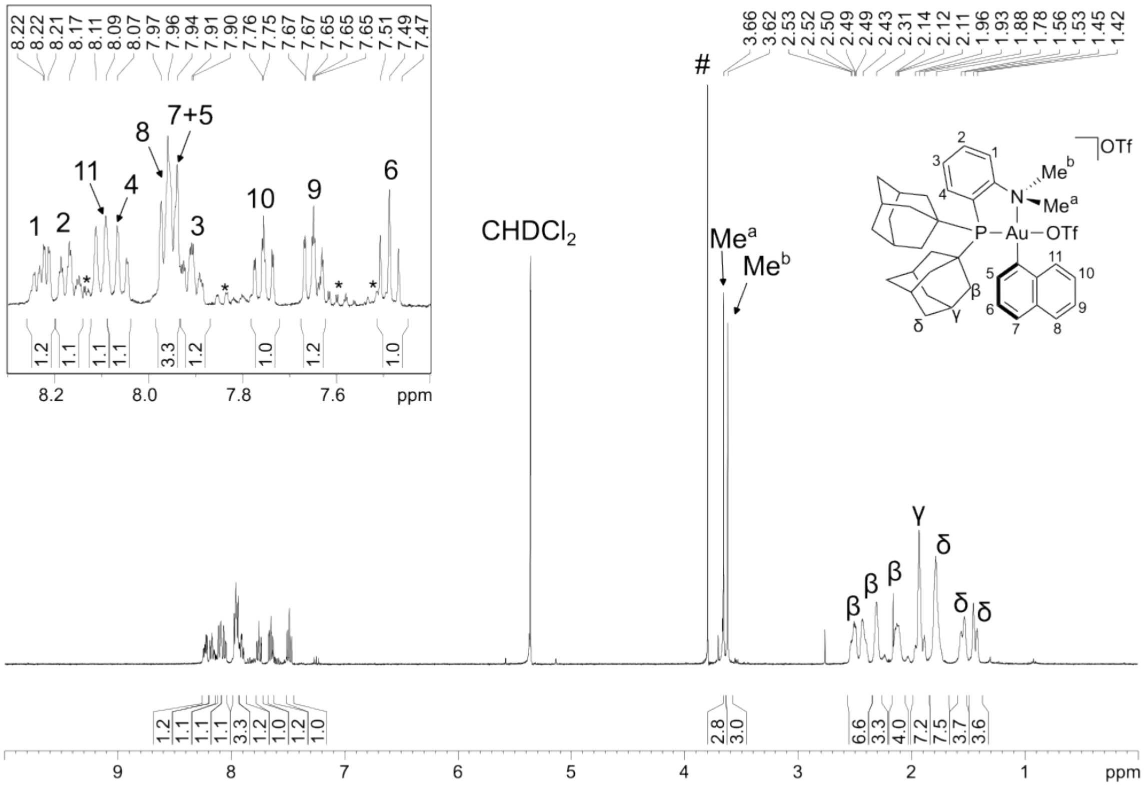

Figure S.6 ^{1}H NMR spectrum of **1naph** obtained *in situ* (CD_2Cl_2, 298 K); asterisks denote excess iodonaphtalene, hashtag denote DCE admixtures.

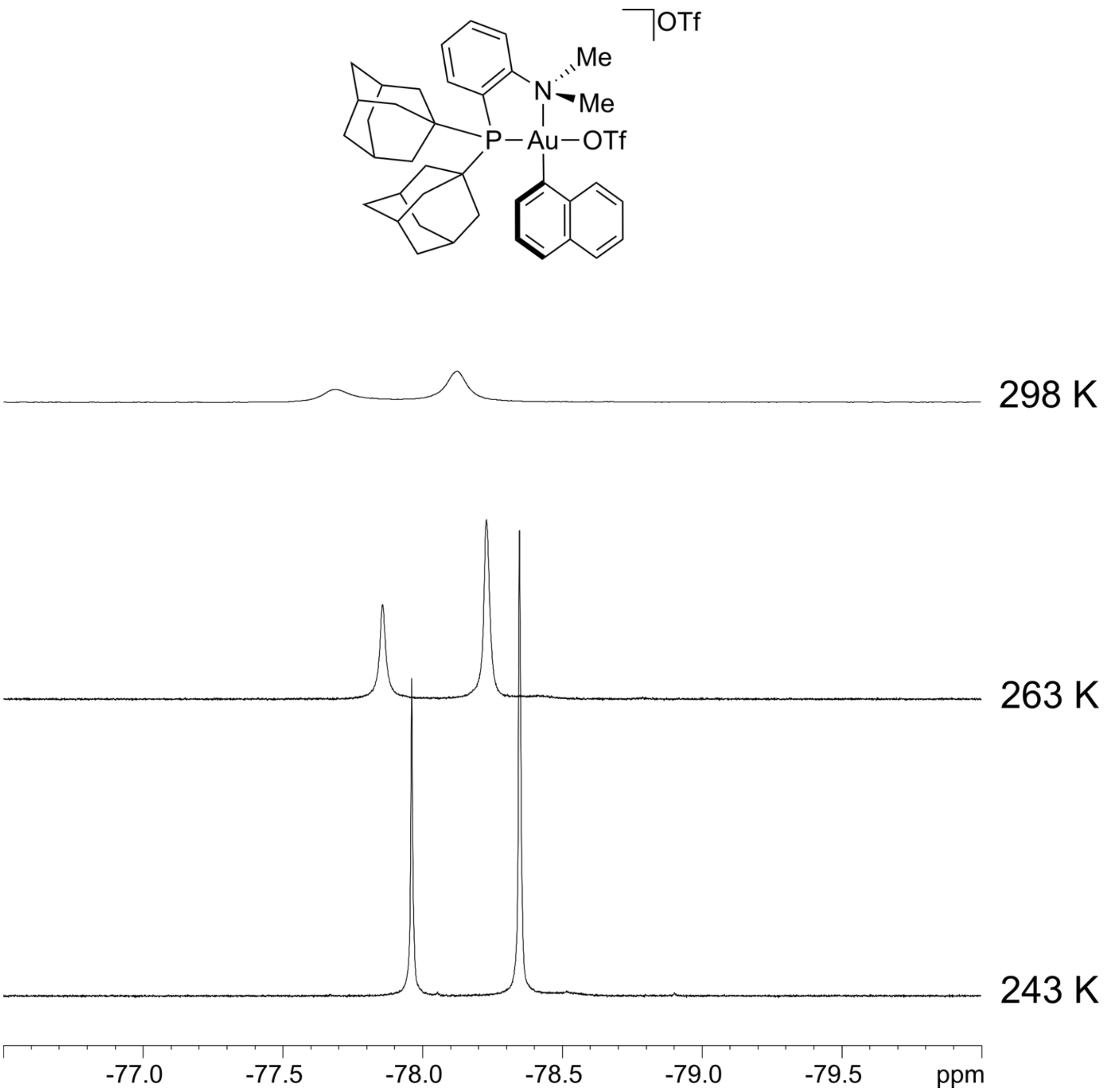

Figure S.7 VT ^{19}F NMR spectra of **1naph** in CD_2Cl_2.

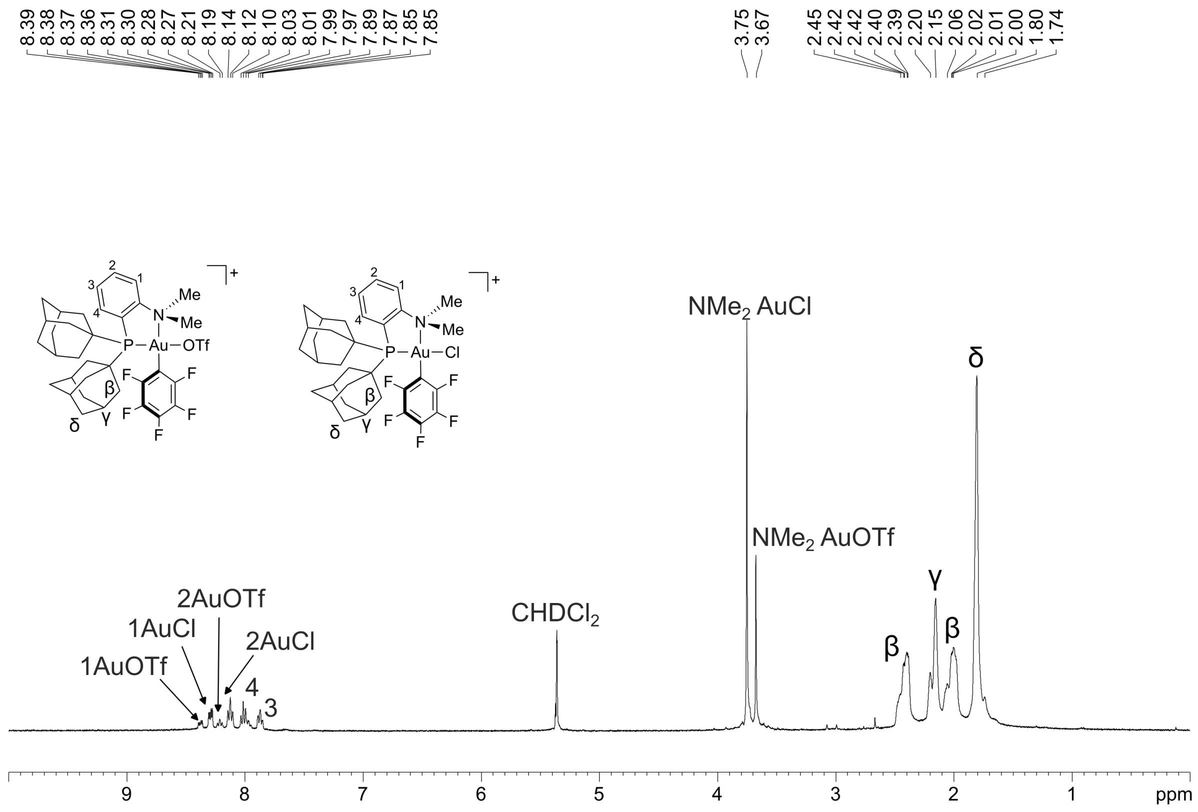

Figure S.8 ^{1}H NMR spectrum obtained after mixing MeDalPhosAuCl with 2.5 equiv of AgOTf and 10 equiv of IC_6F_5 in CD_2Cl_2 and warming up at 55 °C for 2h.

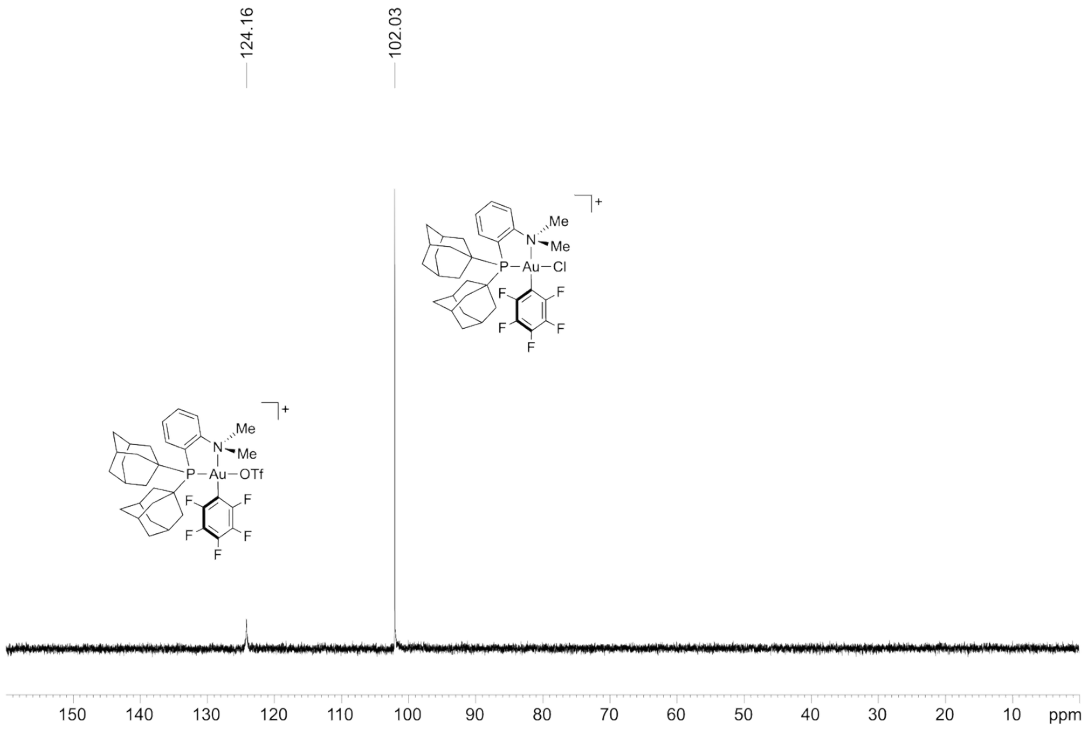

Figure S.9 ^{31}P{^{1}H} NMR spectrum obtained after mixing MeDalPhosAuCl with 2.5 equiv of AgOTf and 10 equiv of IC_6F_5 in CD_2Cl_2 and warming up at 55 °C for 2h.

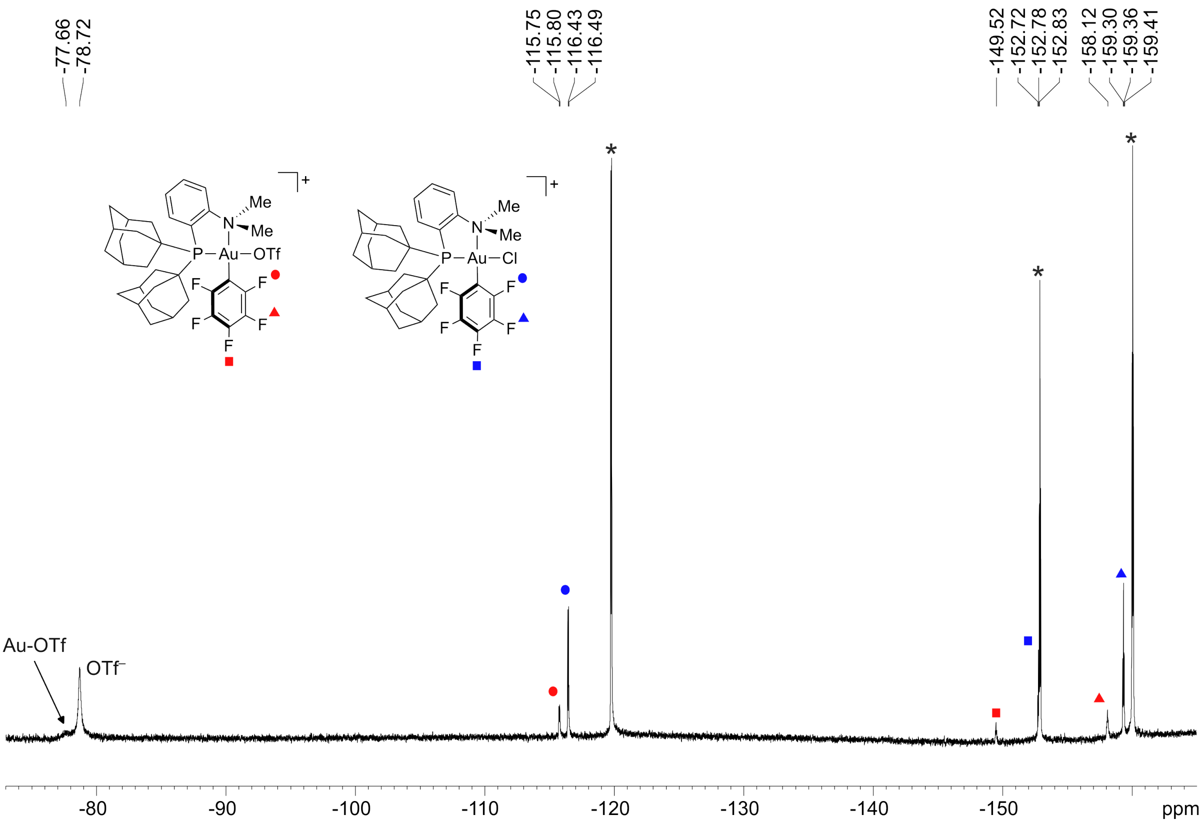

Figure S.10 ^{19}F NMR spectrum obtained after mixing MeDalPhosAuCl with 2.5 equiv of AgOTf and 10 equiv of IC_6F_5 in CD_2Cl_2 and warming up at 55 °C for 2h; asterisks denote excess IC_6F_5.

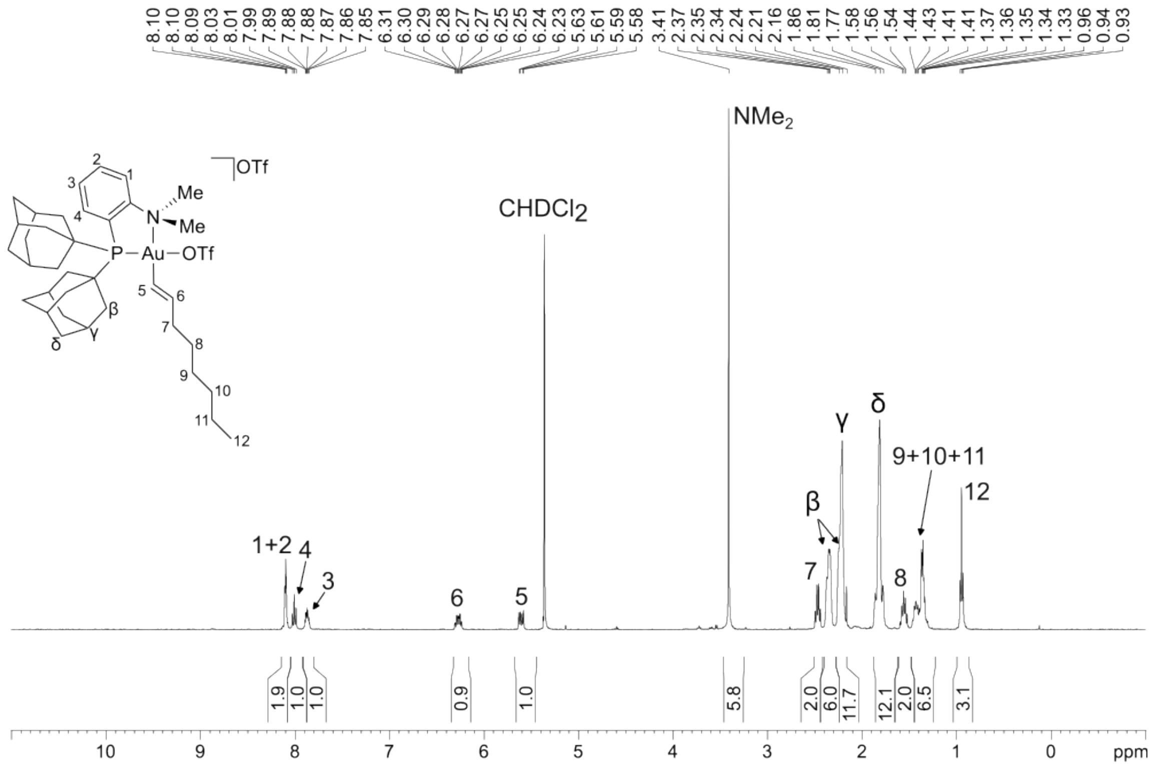

Figure S.11 ^{1}H NMR spectrum of **1C$_8$H$_{15}$** (CD_2Cl_2, 298 K).

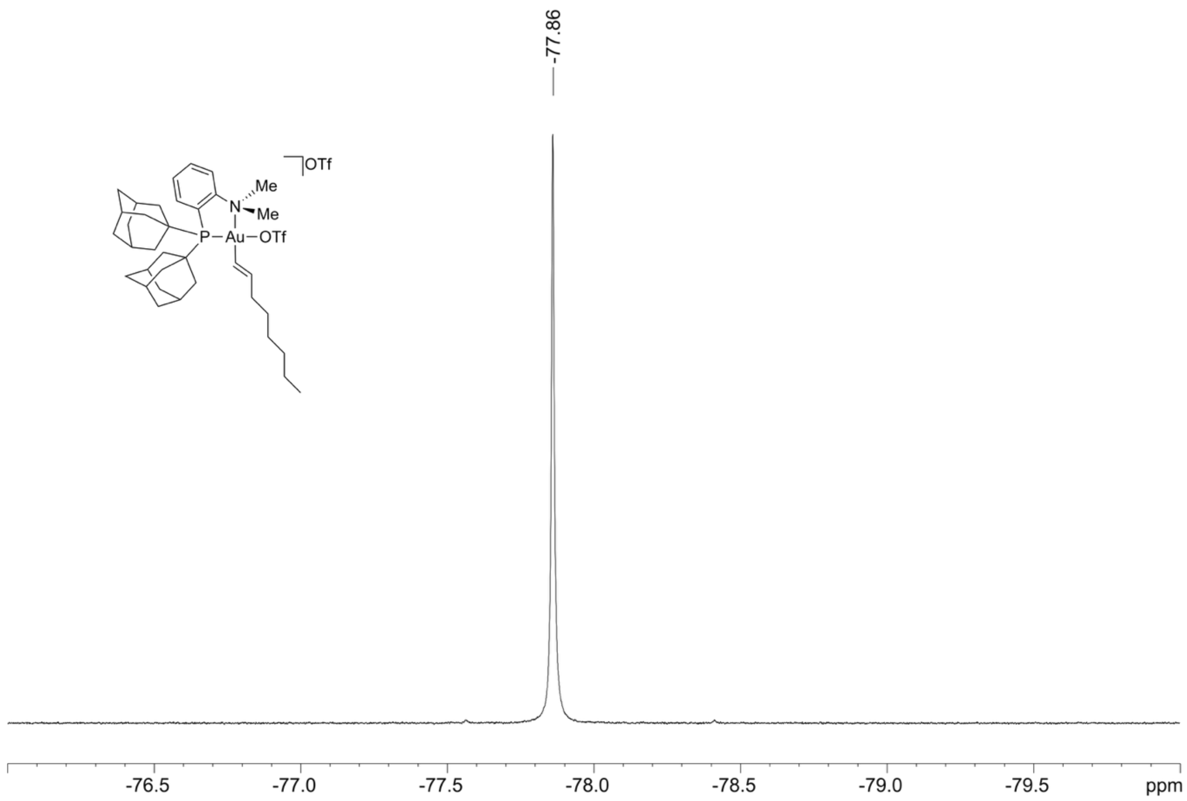

Figure S.12 ^{19}F NMR spectrum of **$1C_8H_{15}$** (CD_2Cl_2, 298 K).

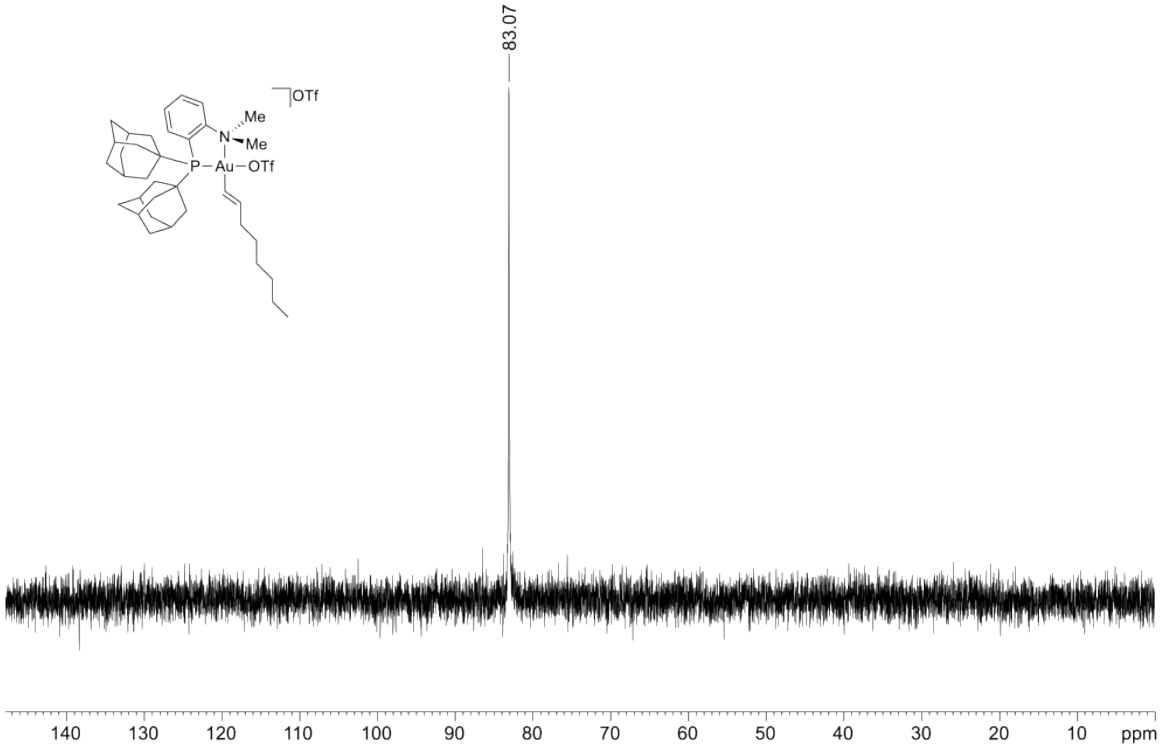

Figure S.13 $^{31}P\{^{1}H\}$ NMR spectrum of **$1C_8H_{15}$** (CD_2Cl_2, 298 K).

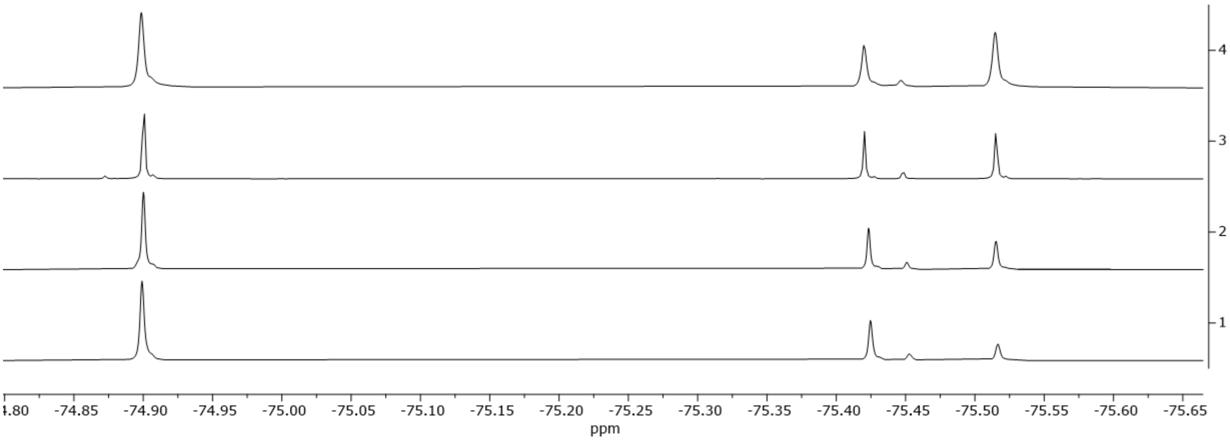

Figure S.14 ^{19}F NMR spectra showing the different distribution of products for the catalytic reaction affording **E/Z 2c/2c'** at different temperatures (1: 25°C, 2:40°C, 3: 60°C, 4:80 °C).

Table S.1 Temperature effect on selectivity for the catalytic carbofunctionalization of 1-phenyl-1-propyne. It should be noted that the ratio of products does not change between 8 and 16 h for reaction conducted at 60°C and 80°C.

**T**	**t**	**2c [%]**		**2c' [%]**		**Total**
**[°C]**	[h]	E	Z	E	Z	[%]
**25**	48	57	26	5	12	100
**40**	24	52	24	5	19	100
**60**	16	43	24	5	28	100
**80**	16	39	22	5	34	100

2. Characterization of organic products

2a - (E)-4-(4-(methoxy)phenyl)hex-3-en-3-yl trifluoromethanesulfonate. ^{1}H NMR (600 MHz, CD_2Cl_2) δ 7.07 (d, *J* = 8.8 Hz, 2H, *H9*, *H13*), 6.89 (d, *J* = 8.8 Hz, 2H, (*H10*, *H12*), 3.79 (s, 5H, *H15*), 2.46 (m, 3H, *H3*), 2.23 (m, 3H, *H6*), 1.00 (t, *J* = 7.4 Hz, 5H, *H8*), 0.87 (t, *J* = 7.5 Hz, 5H, *H7*). ^{13}C{^{1}H} NMR (151 MHz, CD_2Cl_2) δ 159.3 (*C11*), 147.4 (*C1*), 135.6 (*C2*), 129.5 (*C4*), 129.5 (*C9*, *C13*), 120.6 (q, *J* = 319.3 Hz, *C19*), 113.9 (*C10*, *C12*), 55.2 (*C15*), 25.7 (*C3*), 24.4 (*C6*), 11.5 (*C8*), 11.3 (*C7*). ^{19}F NMR (565 MHz, CD_2Cl_2) δ -75.51 (*F20, F21, F22*).

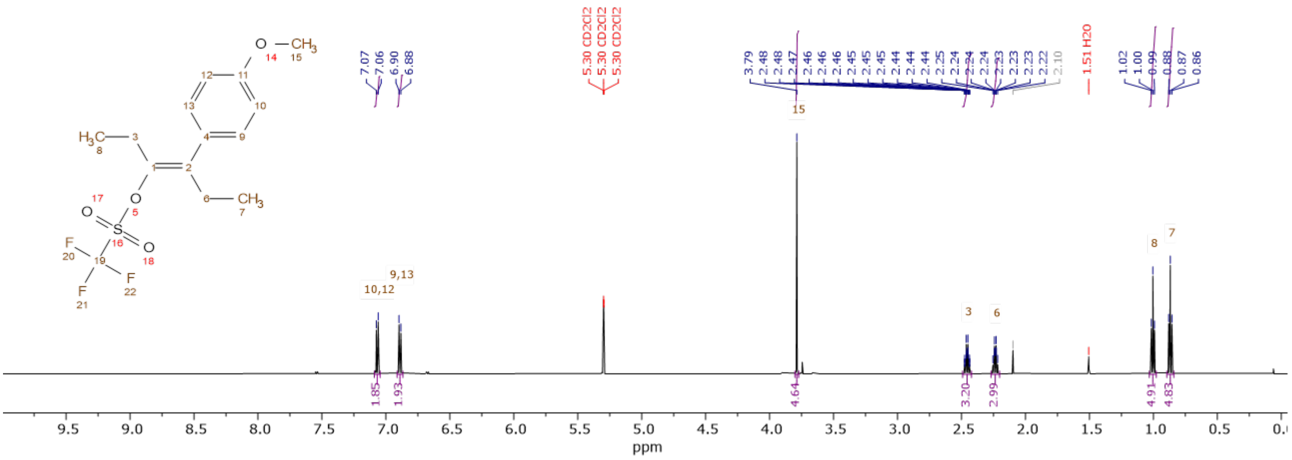

Figure S.15 ^{1}H NMR spectrum of **2a** (CD_2Cl_2, 298K).

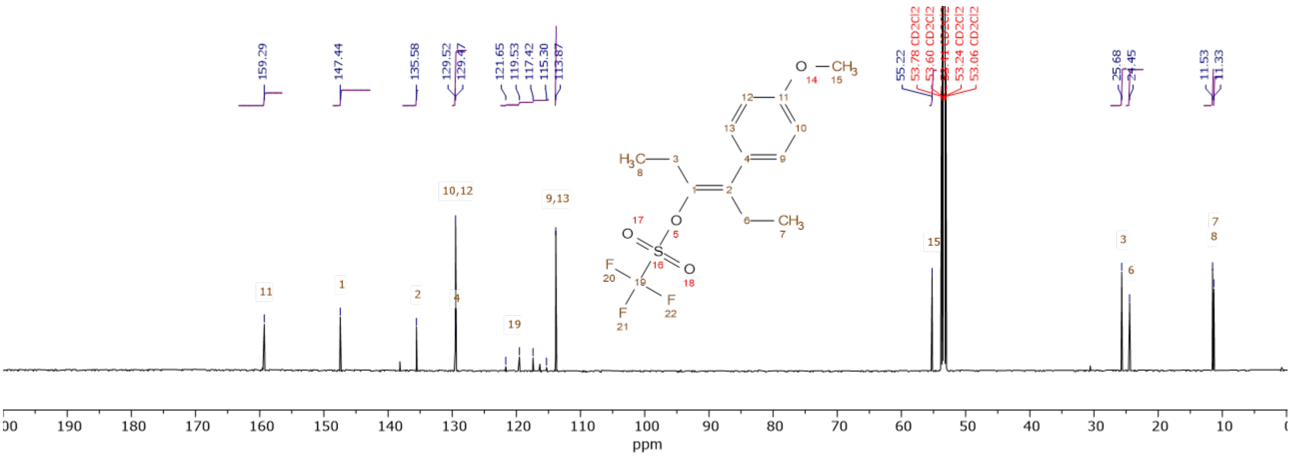

Figure S.16 ^{13}C{^{1}H} NMR spectrum of **2a** (CD_2Cl_2, 298K).

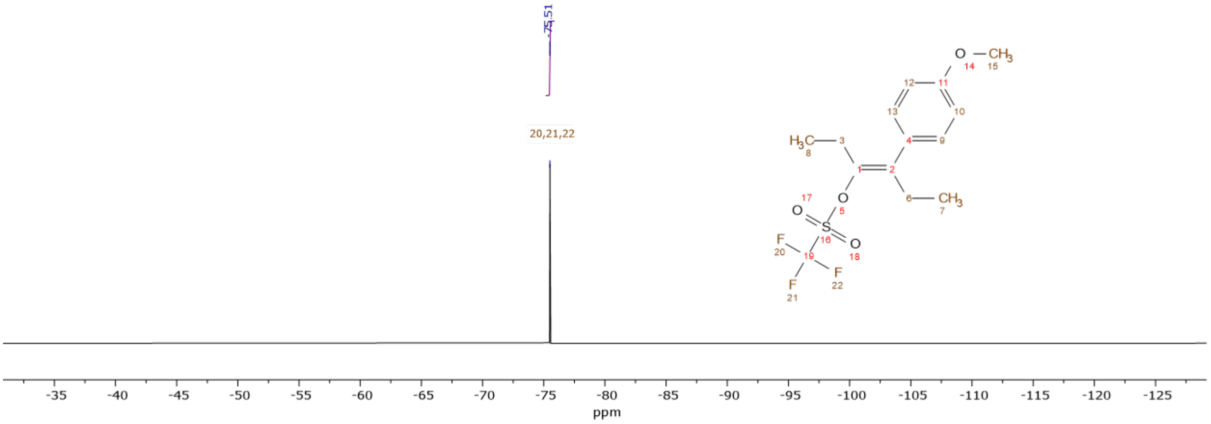

Figure S.17 ^{19}F NMR spectrum of **2a** (CD_2Cl_2, 298K).

2b/2b'- (E)-3-(4-methoxyphenyl)hex-2-en-2-yl trifluoromethanesulfonate / (E)-2-(4-methoxyphenyl)hex-2-en-3-yl trifluoromethanesulfonate. ^{1}H NMR (400 MHz, CD_2Cl_2) δ 7.15 (dd, *J* = 9.3, 7.4 Hz, 1H, *H34*, *H38*, *H9*, *H13*), 6.94 (dd, *J* = 8.8, 2.1 Hz, 1H, *H35*, *H37*, *H10*, *H12*), 3.85 (s, 2H, *H40*, *H15*), 2.49 (m, 1H, *H27*), 2.32 (m, 1H, *H5*), 2.11 (s, 1H, *H3*), 2.00 (s, 1H, *H25*), 1.52 (m, 1H, *H6*), 1.32 (m, 1H, *H28*), 0.90 (t, *J* = 7.4 Hz, 1H, *H29*), 0.85 (t, *J* = 7.4 Hz, 1H, *H7*). ^{13}C{^{1}H} NMR (101 MHz, CD_2Cl_2) δ 159.2 (*C11, C36*), 146.9 (*C2*), 142.6 (*C23*), 134.5 (*C24*), 131.3 (C4), 130.7 (*C1*), 129.7 (C30), 129.6 (*C34*, *C38*), 129.0 (*C9*, *C13*), 120.0 (q, *J* = 319.0 Hz, *C41*), 119.2 (q, *J* = 319.0 Hz, *C19*), 113.9 (*C35*, *C37*), 113.8 (*C10*, *C12*), 55.2 (*C40*, *C15*), 34.1 (*C27*), 32.6 (*C5*), 20.5 (*C28*), 19.9 (*C6*), 19.4 (*C3*), 17.4 (*C25*), 13.5 (*C29*), 13.0 (*C7*). ^{19}F NMR (376 MHz, CD_2Cl_2) δ -75.40 (*42, 43, 44*), -75.46 (*20, 21, 22*).

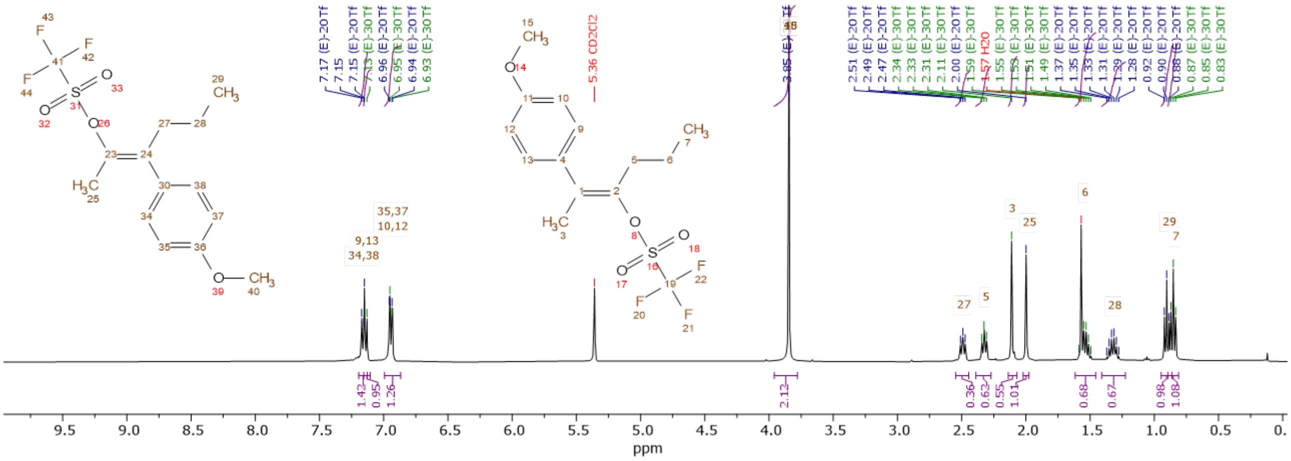

Figure S.18 ^{1}H NMR spectrum of **2b/2b'** (CD_2Cl_2, 298K).

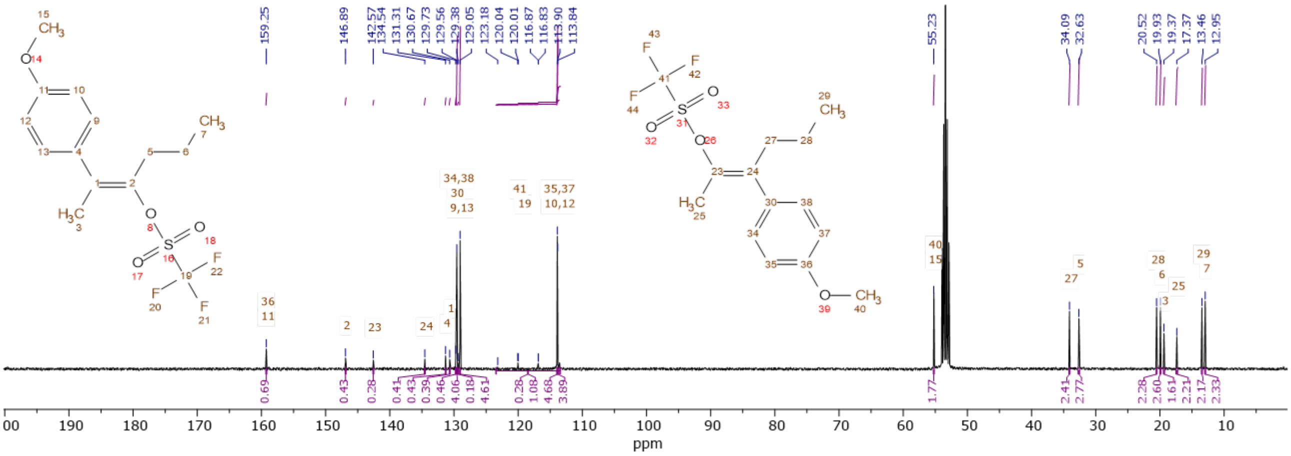

Figure S.19 ^{13}C{^{1}H} NMR spectrum of **2b/2b'** (CD_2Cl_2, 298K).

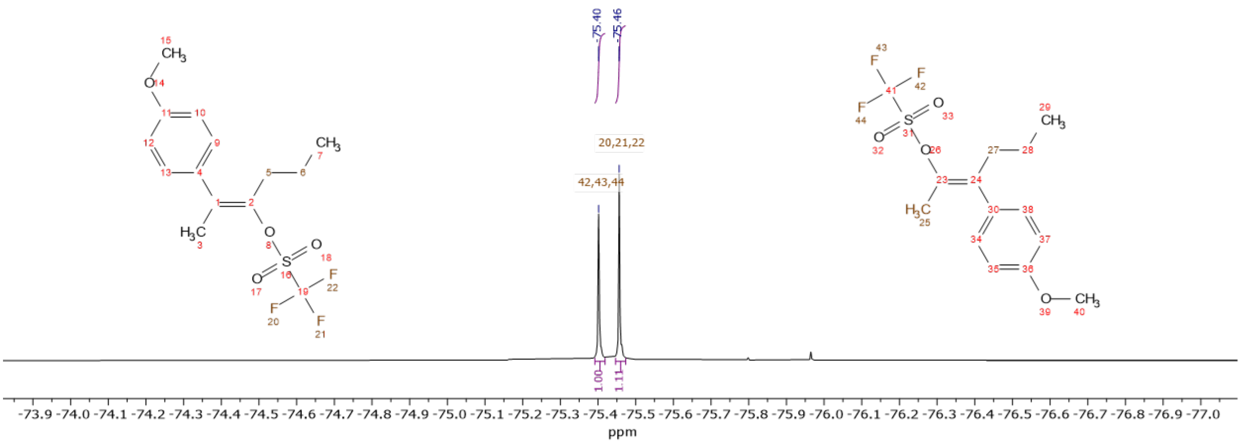

Figure S.20 ^{19}F NMR spectrum of **2b/2b'** (CD_2Cl_2, 298K).

E-2c, **Z-2c, E-2c'** and **Z-2c'**- (Z)-2-(4-methoxyphenyl)-1-phenylprop-1-en-1-yl trifluoromethanesulfonate - (E)-2-(4-methoxyphenyl)-1-phenylprop-1-en-1-yl trifluoromethanesulfonate - (E)-1-(4-methoxyphenyl)-1-phenylprop-1-en-2-yl trifluoromethanesulfonate - (Z)-1-(4-methoxyphenyl)-1-phenylprop-1-en-2-yl trifluoromethanesulfonate. ^{1}H NMR (400 MHz, CD_2Cl_2) δ 7.57 (dd, *J* = 7.3, 2.5 Hz, 1H, *H37, H41*), 7.50-7.20 (m, ArCH, 8H), 7.40 (d, *J* = 8.7 Hz, 1H, *H54-H56*), 7.16 (d, *J* = 8.7 Hz, 1H, *H29, H31*), 7.05 (d, *J* = 8.8 Hz, 1H, *H4, H6*), 7.00 (d, *J* = 8.8 Hz, 1H, *H51, H53*), 6.93 (d, *J* = 8.8 Hz, 1H, *H26, H28*), 6.86 (d, *J* = 8.7 Hz, 0H, *H76,H78*), 6.77 (d, *J* = 8.8 Hz, 1H, *H1, H3*), 3.87 (s, 1H, *H66*), 3.84 (s, 2H, *H46*), 3.83 (s, 0H, *H91*), 3.77 (s, 3H, *H21*), 2.32 (s, 3H, *H11*), 2.26 (s, 2H, *H36*), 2.22 (s, 0H, *H85*), 2.11 (s, 2H, *H60*). ^{13}C{^{1}H} NMR (101 MHz, CD_2Cl_2) δ 159.6 (*C27*), 159.3 (*C52*), 159.1 (*C2*), 130.9 (m, *C5, C29, C30, C31*), 129.8 (m, *C4, C6, C12, C16, C37, C41*), 129.3 (*C54, C56*), 128.1 (C67, C71), 114.2 (C76, C78), 113.8 (m, C26, C28, C51, C53), 113.7 (C1, C3), 55.3 (*C46*), 55.2 (*C66*), 55.1 (*C21*), 20.8 (*C60*), 19.7 (*C11*), 18.7 (*C85*, *C36*). ^{19}F NMR (565 MHz, CD_2Cl_2) δ -74.95 (23, 24, 25), -75.47 (48, 49, 50), -75.50 (98, 99, 100), -75.56 (73, 74, 75).

Note: the target compounds were identified in the mixture, but complete assignment was not possible due to overlapping signals and poor concentration of the minor isomers.

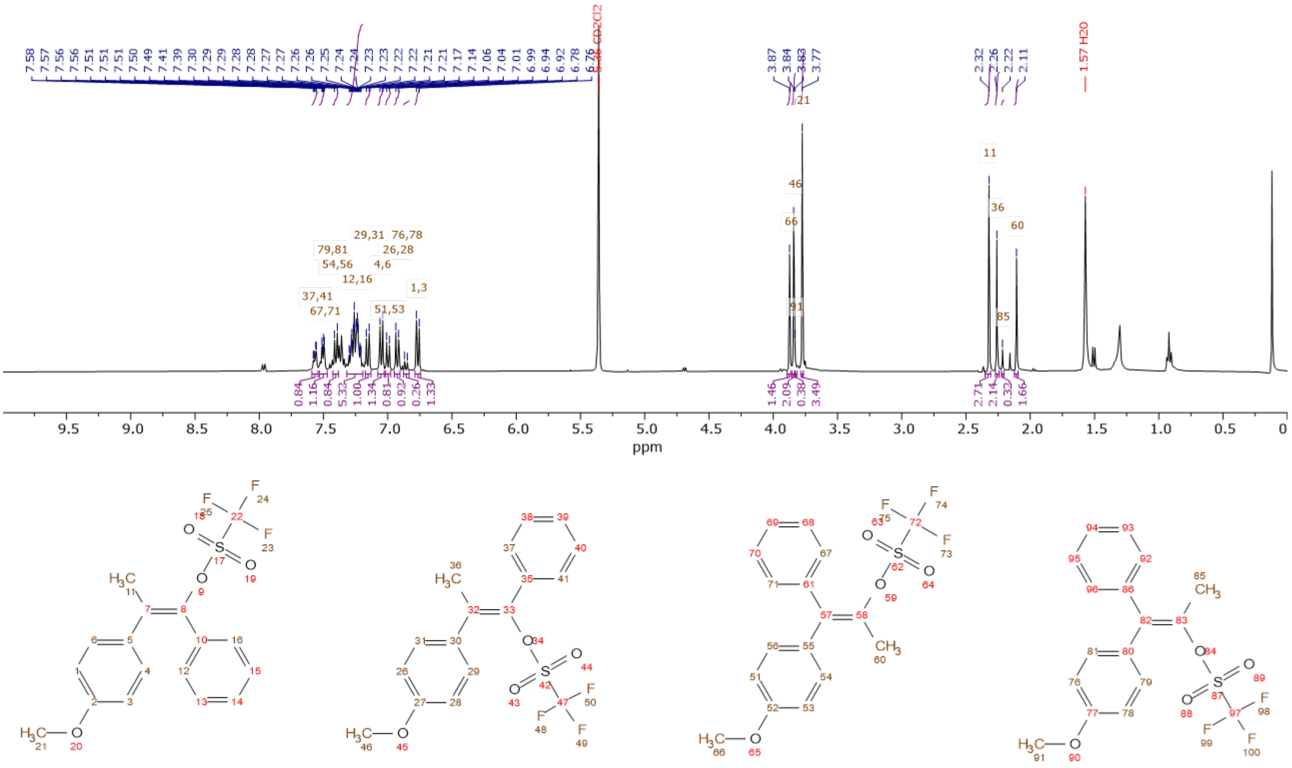

Figure S. 21 ^{1}H NMR spectrum of **Z** and **E 2c/2c'** (CD_2Cl_2, 298K).

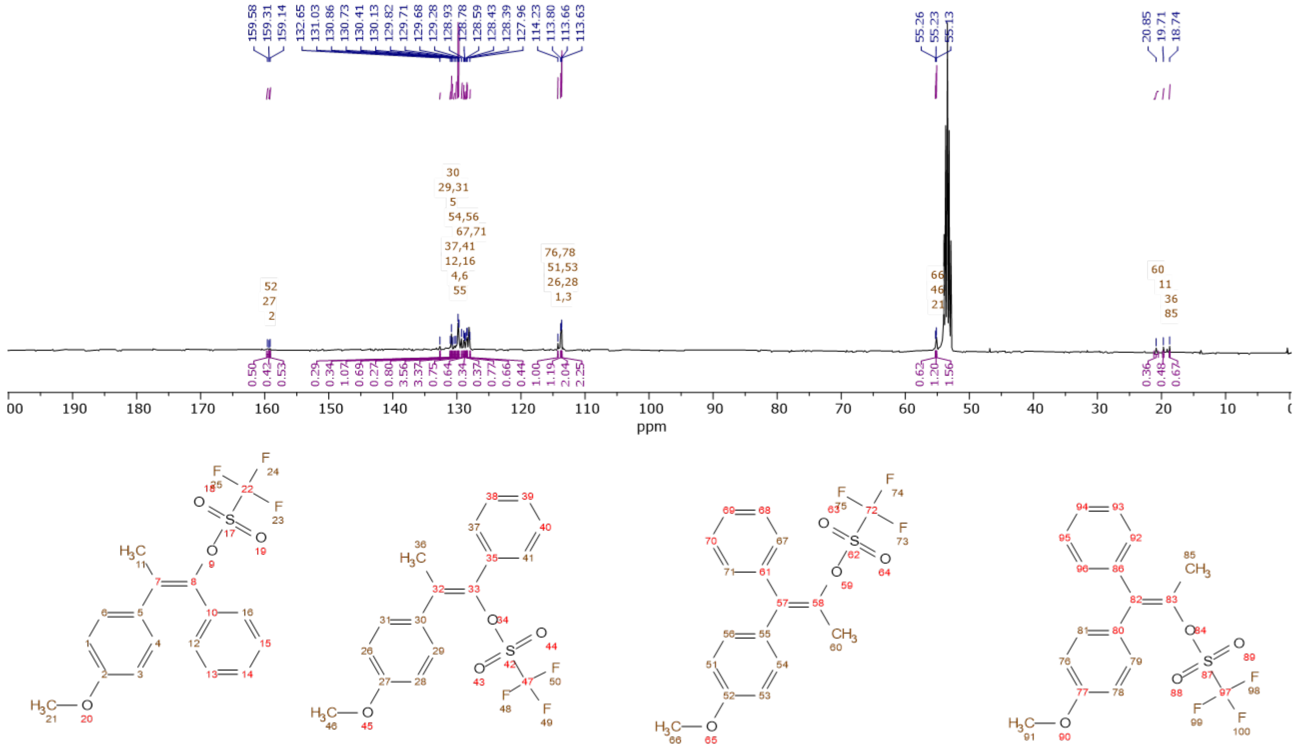

Figure S.22 $^{13}C\{^{1}H\}$ NMR spectrum of **Z** and **E 2c/2c'** (CD_2Cl_2, 298K).

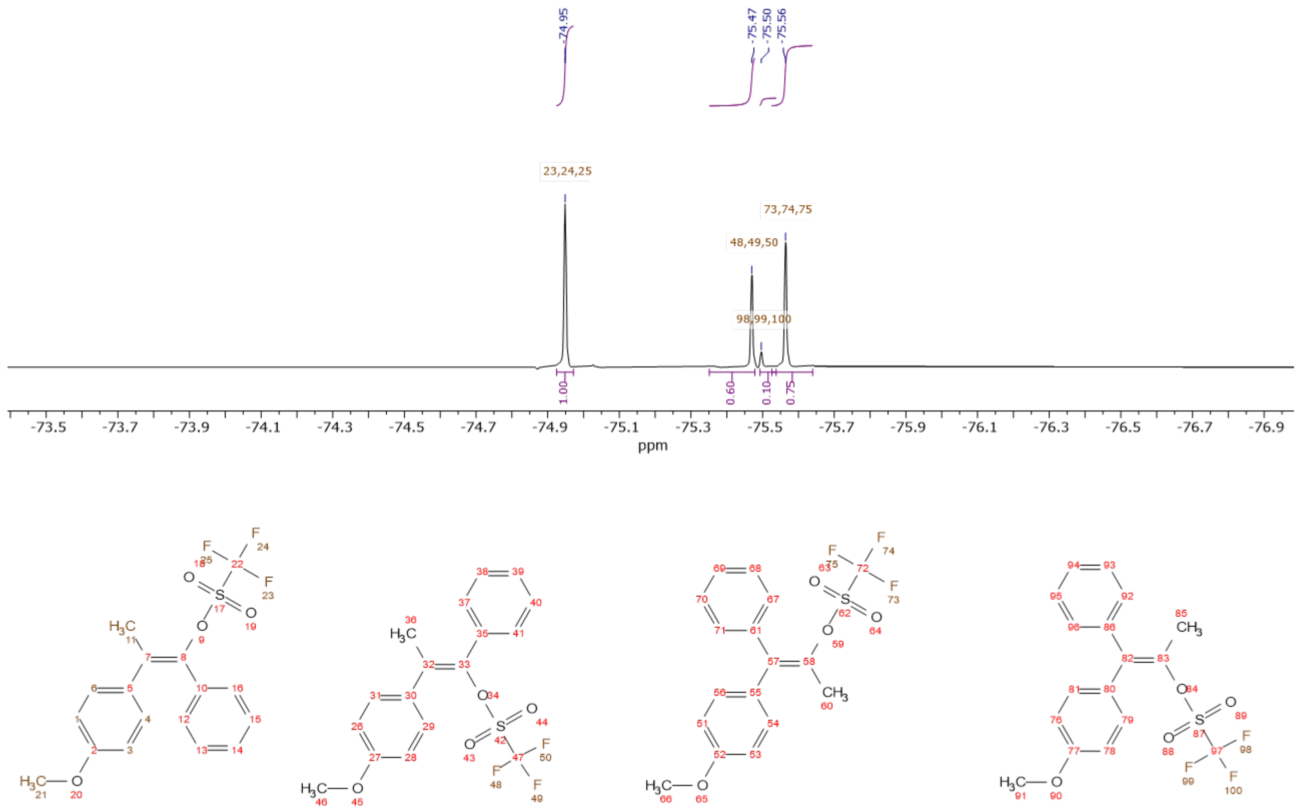

Figure S.23 ^{19}F NMR spectrum of **Z** and **E 2c/2c'** (CD_2Cl_2, 298K).

E-2d and **Z-2d** - methyl (Z)-2-(4-methoxyphenyl)-3-phenyl-3-(((trifluoromethyl)sulfonyl)oxy)acrylate and methyl / (E)-2-(4-methoxyphenyl)-3-phenyl-3-(((trifluoromethyl)sulfonyl)oxy)acrylate. ^{1}H NMR (400 MHz, CD_2Cl_2) δ 7.60 (dd, *J* = 7.8, 1.8 Hz, 1H, *H37*, *H41*), 7.52 (m, 3H), 7.35 (m, 3H, *H9*, *H10*, *H11*, *H12*, *H13*), 7.09 (d, *J* = 8.8 Hz, 1H), 7.02 (d, *J* = 8.8 Hz, 1H, *H43*, *H45*), 6.79 (d, *J* = 8.8 Hz, 1H, *H15*, *H17*), 3.90 (s, 2H), 3.88 (s, 2H, *H48*), 3.78 (s, 2H), 3.59 (s, 2H). ^{13}C{^{1}H} NMR (101 MHz, CD_2Cl_2) δ 166.5 (*C32*), 165.7 (C4), 160.4 (*C44*), 159.9 (16), 148.3 (*C29*), 147.7 (*C1*), 132.2 (*C31*), 131.3 (*C3*), 131.0 (*C14*, *C18*), 130.4 (*C11*), 130.4 (*C42*, *C46*), 129.8 (*C10*, *C12*), 129.1 (*C2*), 128.6 (*C37*, *C38*, *C39*, *C40*, *C41*), 128.4 (*C9*, *C13*), 128.3 (*C30*), 124.2 (*C5*), 123.1 (*C33*), 118.0 (q, *J* = 322.0 Hz, *C25*), 116.4 (q, *J* = 322.0 Hz, *C53*), 113.9 (*C43*, *C45*), 113.9 (C15, C17), 55.3 (*C48*), 55.2 (*C20*), 52.7 (*C8*), 52.6 (*C36*). ^{19}F NMR (376 MHz, CD_2Cl_2) δ -74.60 (*F26, F27, F28*), -74.93 (*F54, F55, F56*).

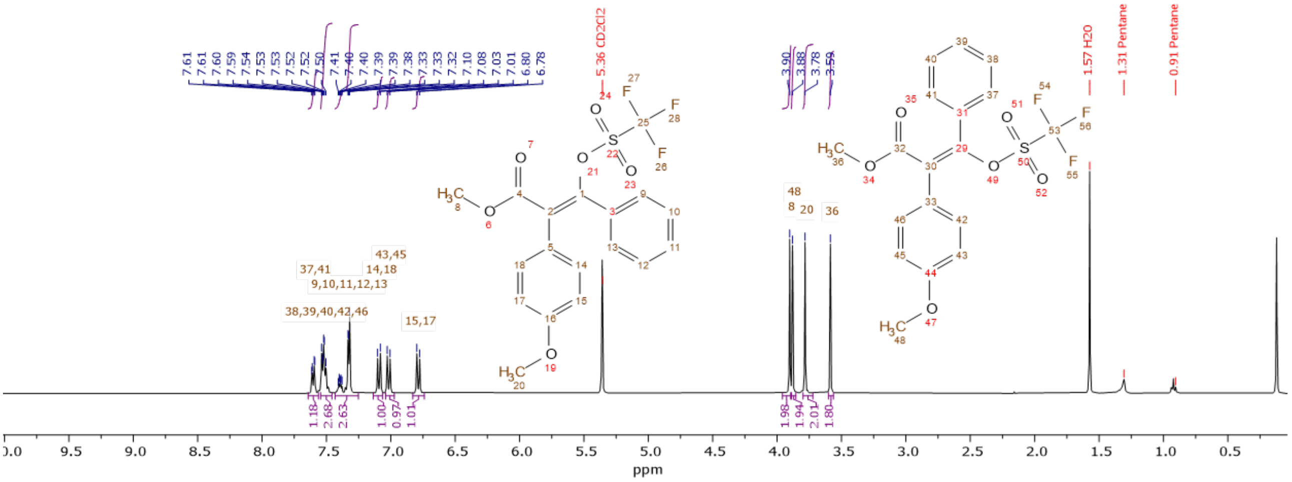

Figure S.24 ^{1}H NMR spectrum of **E-2d** and **Z-2d** (CD_2Cl_2, 298K).

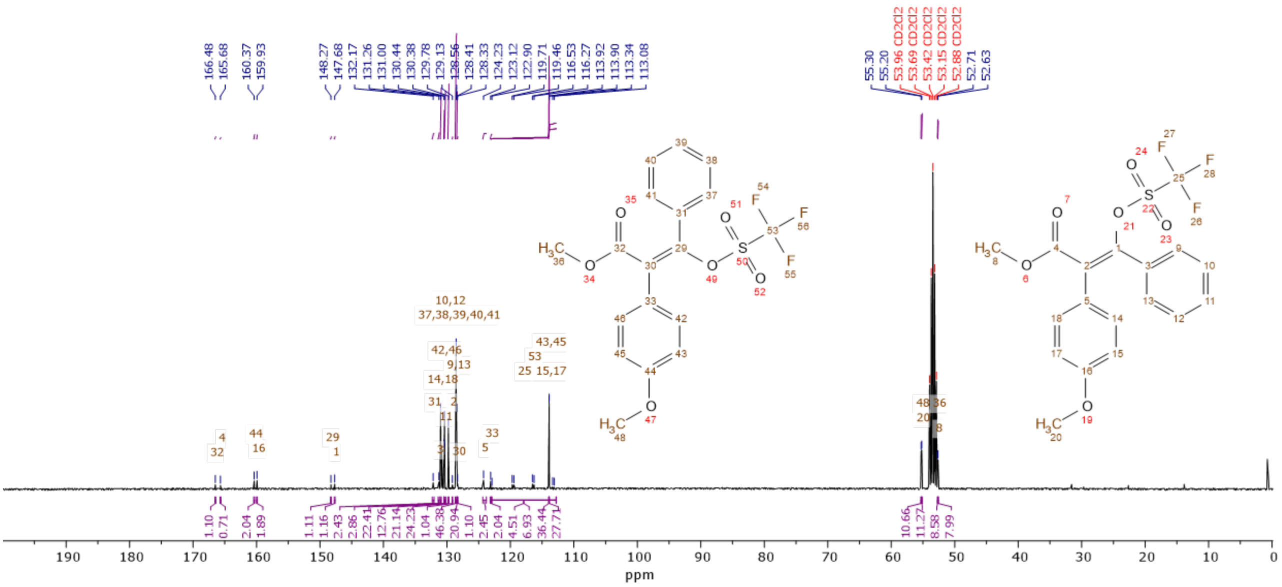

Figure S.25 ^{13}C{^{1}H} NMR spectrum of **E-2d** and **Z-2d** (CD_2Cl_2, 298K).

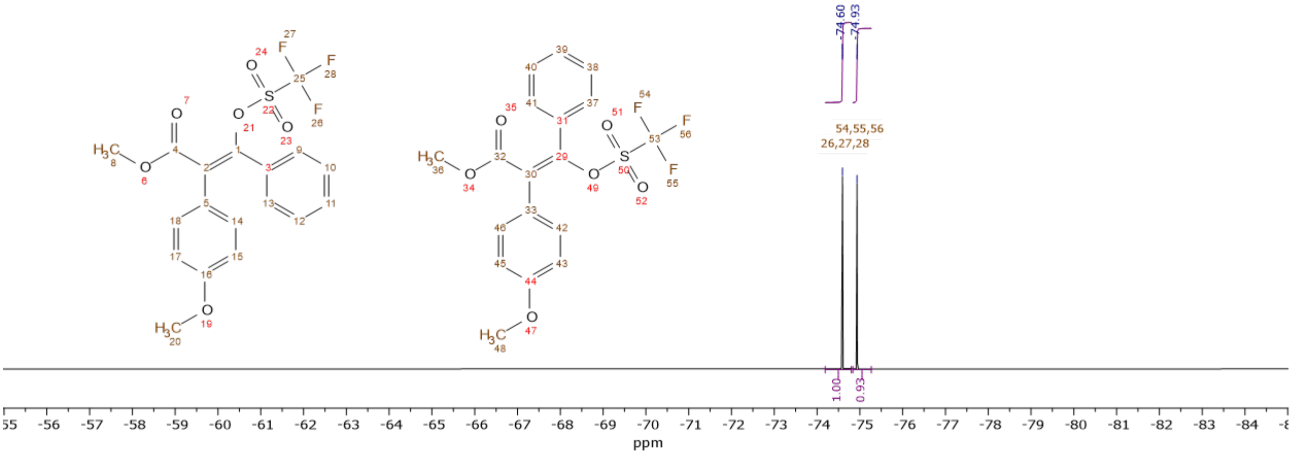

Figure S. 26 ^{19}F NMR spectrum of **E-2d** and **Z-2d** (CD_2Cl_2, 298K).

E-2d and **Z-2d** could be separated by small-scale chromatography (pentane eluent) in very low yield (<10%).

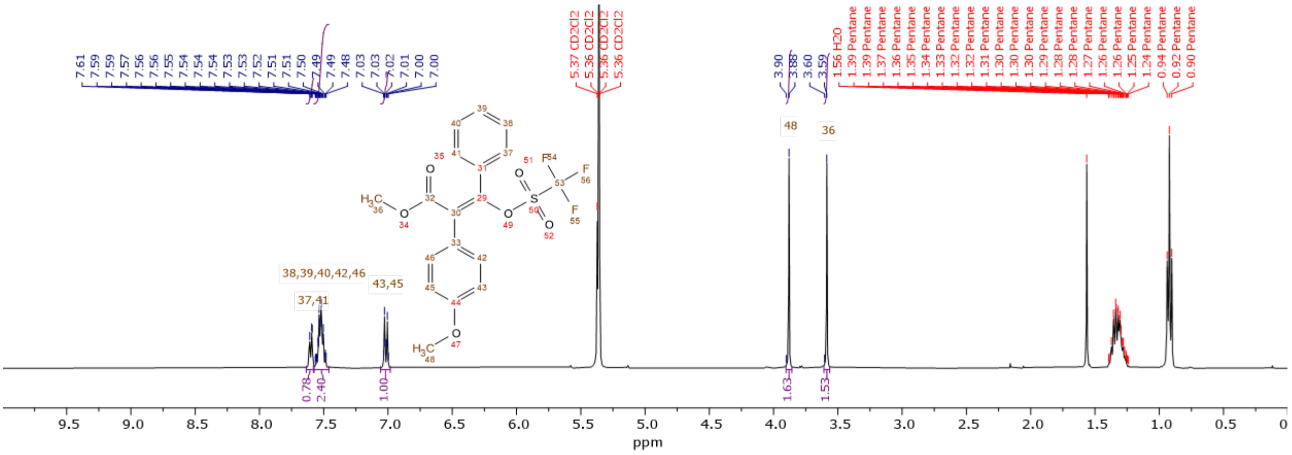

Figure S.27 ^{1}H NMR spectrum of purified **Z-2d** (CD_2Cl_2, 298K); extra solvent peaks are due to residual pentane.

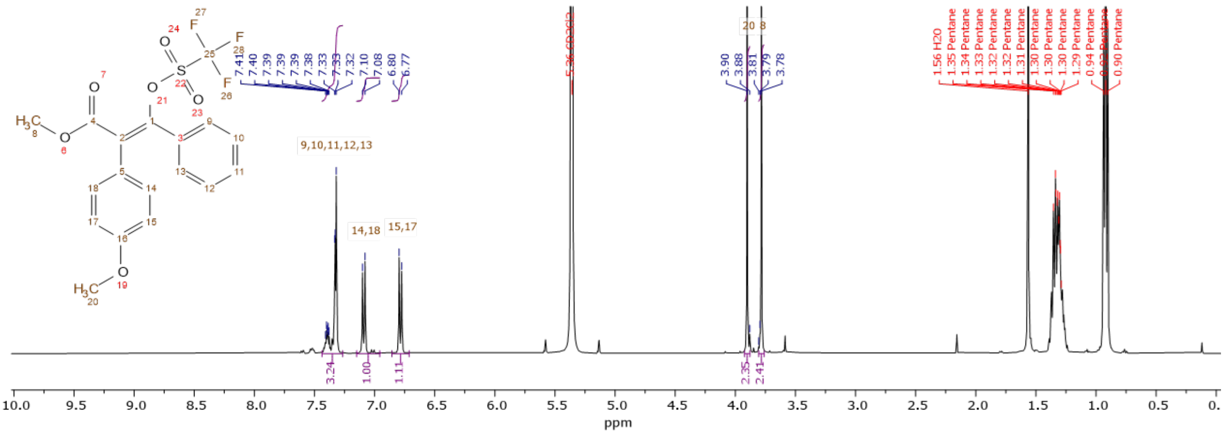

Figure S.28 ^{1}H NMR spectrum of purified **E-2d** (CD_2Cl_2, 298K); extra solvent peaks are due to residual pentane.

E-2e and Z-2e - ethyl (Z)-2-(4-methoxyphenyl)-3-(((trifluoromethyl)sulfonyl)oxy)but-2-enoate and ethyl (E)-3-(4-methoxyphenyl)-2-(((trifluoromethyl)sulfonyl)oxy)but-2-enoate. ^{1}H NMR (600 MHz, CD_2Cl_2) δ 7.20 (m, 1H, *H32, H36*), 7.18 (m, 1H, *H8*, *H12*), 6.91 (m, 1H, *H33, H35*), 6.89 (m, 1H, *H9*, *H11*), 4.21 (q, *J* = 7.1 Hz, 2H, *H43*, *H19*), 3.80 (s, 2H, *H41*), 3.79 (s, 1H, *H17*), 2.43 (s, 1H, *H6*), 2.06 (s, 2H, *H30*), 1.25 (t, *J* = 7.1 Hz, 2H, *H44*), 1.23 (t, *J* = 7.0 Hz, 1H, *H20*). ^{13}C{^{1}H} NMR (151 MHz, CD_2Cl_2) δ 164.8 (*C28*), 160.1 (*C34*), 159.9 (*C10*), 152.2 (*C1*), 148.5 (*C25*), 128.3 (C2), 128.3 (*C26*), 124.6 (*C27*), 124.1 (*C3*), 118.8 (m, *C45, C21*), 62.0 (*C43*), 61.9 (*C19*), 55.3 (*C41*), 55.2 (*C17*), 19.1 (*C6*), 18.0 (*C30*), 13.8 (*C20*), 13.7 (*C44*). ^{19}F NMR (565 MHz, CD_2Cl_2) δ -74.96(*F46, F47, F48*), -75.31(*F22, F23, F24*).

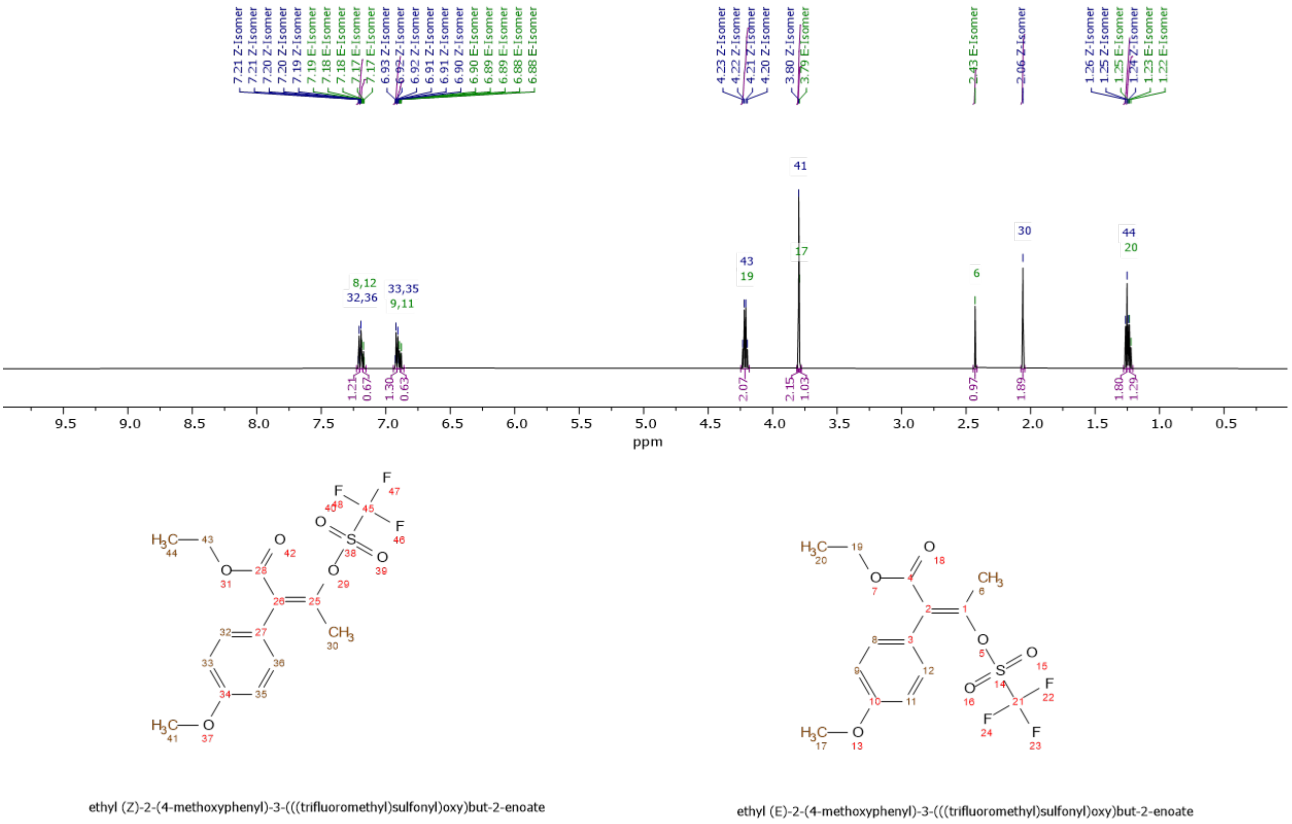

Figure S.29 ^{1}H NMR spectrum of **E-2e** and **Z-2e** (CD_2Cl_2, 298K).

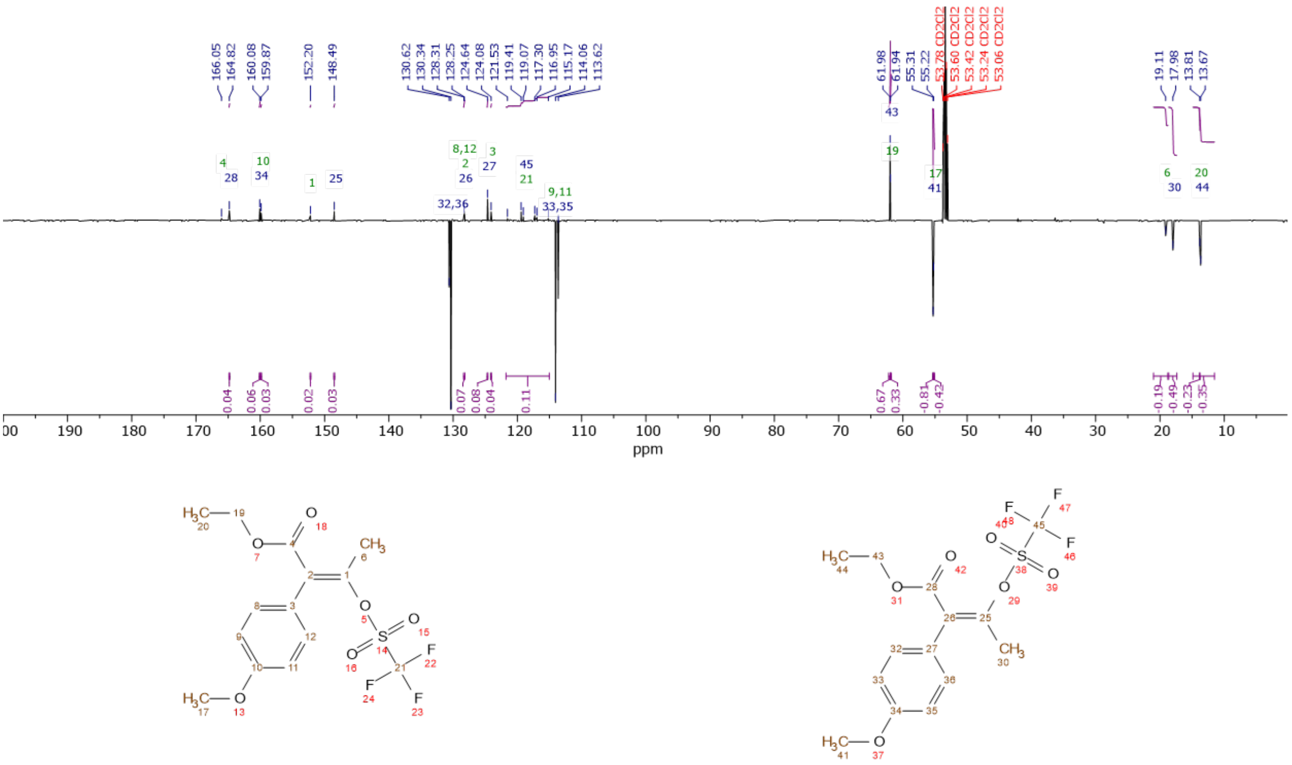

Figure S.30 $^{13}C\{^1H\}$ Jmod NMR spectrum of **E-2e** and **Z-2e** (CD_2Cl_2, 298K).

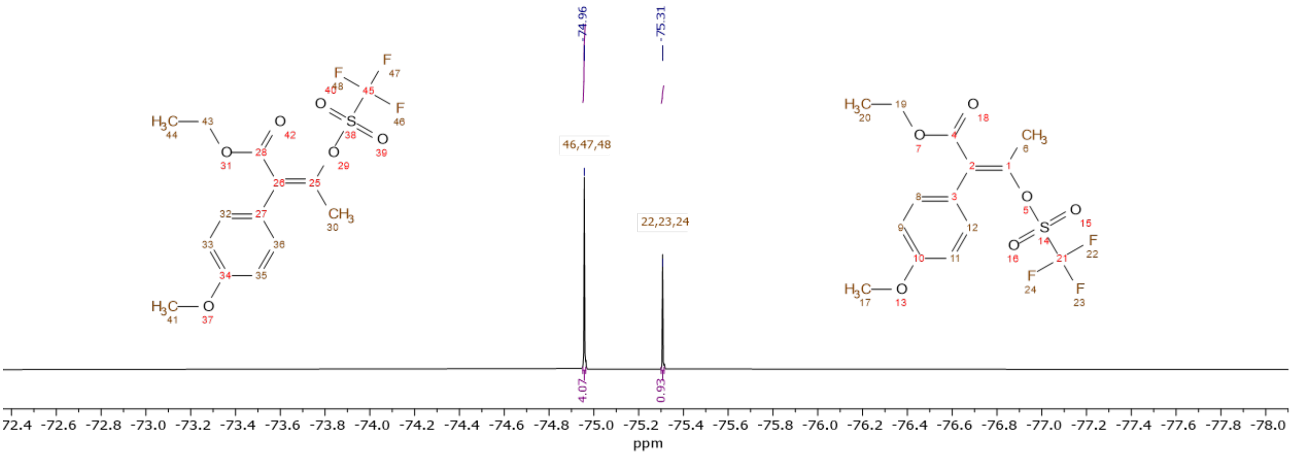

Figure S.31 ^{19}F NMR spectrum of **E-2e** and **Z-2e** (CD_2Cl_2, 298K).

2g - 3-(4-methoxyphenyl)-2-methyl-6-oxo-4,6-dihydro-[1,3]oxazino[2,3-a]isoindol-5-ium. ^{1}H NMR (400 MHz, CD_2Cl_2) δ 8.11 (dd, *J* = 7.3, 0.8 Hz, 1H, *H6*), 8.08 (m, 1H, *H3*), 8.05 (m, 1H, *H1*), 7.97 (td, *J* = 7.4, 1.5 Hz, 1H, *H2*), 7.34 (m, 2H, *H17*, *H21*), 7.03 (m, 2H, *H18*, *H20*), 4.59 (q, *J* = 2.0 Hz, 2H, *H12*), 3.88 (s, 3H, *H23*), 2.25 (t, *J* = 1.9 Hz, 3H, *H15*). ^{13}C{^{1}H} NMR (101 MHz, CD_2Cl_2) δ 172.6 (*C9*), 163.2 (*C7*), 160.57 (*C19*), 144.8 (*C14*), 138.4 (*C1*), 136.1 (*C2*), 129.4 (*C17*, *C21*), 127.9 (*C4*), 127.62 (*C5*), 126.8 (*C3*), 126.4 (*C6*), 114.6 (*C18*, *C20*), 114.0 (*C13*), 55.4 (*C23*), 41.3 (*C12*), 15.6 (*C15*). ^{19}F NMR (376 MHz, CD_2Cl_2) δ -78.91 (*F29, F30, F31*).

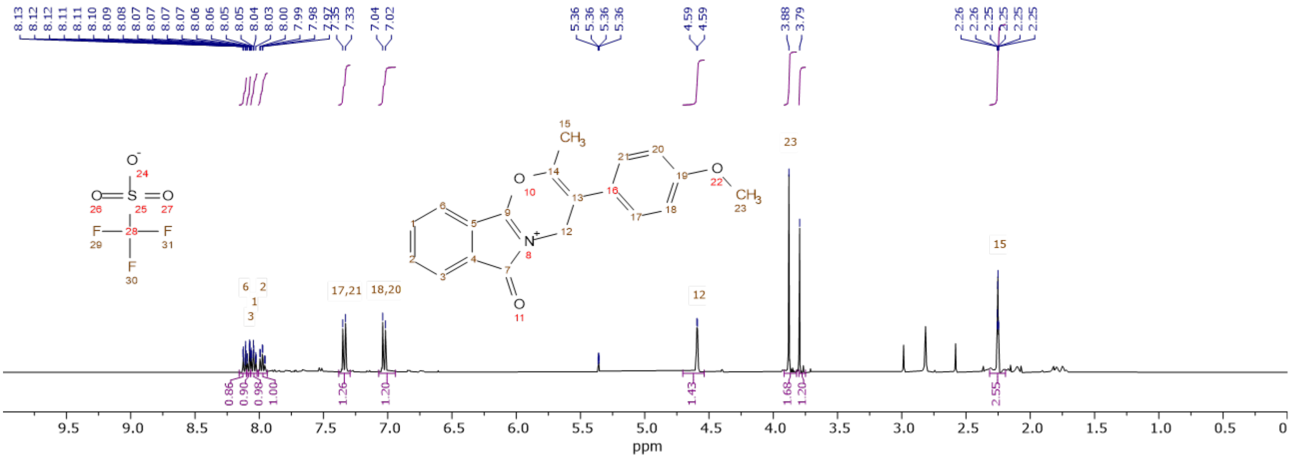

Figure S.32 ^{1}H NMR spectrum of **2g** (CD_2Cl_2, 298K).

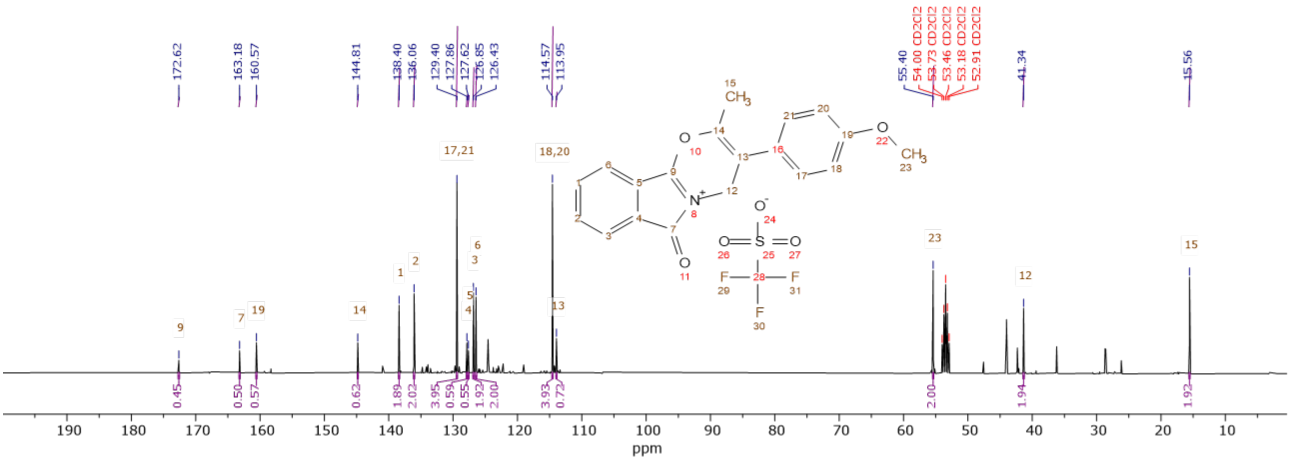

Figure S.33 ^{13}C{^{1}H} NMR spectrum of **2g** (CD_2Cl_2, 298K).

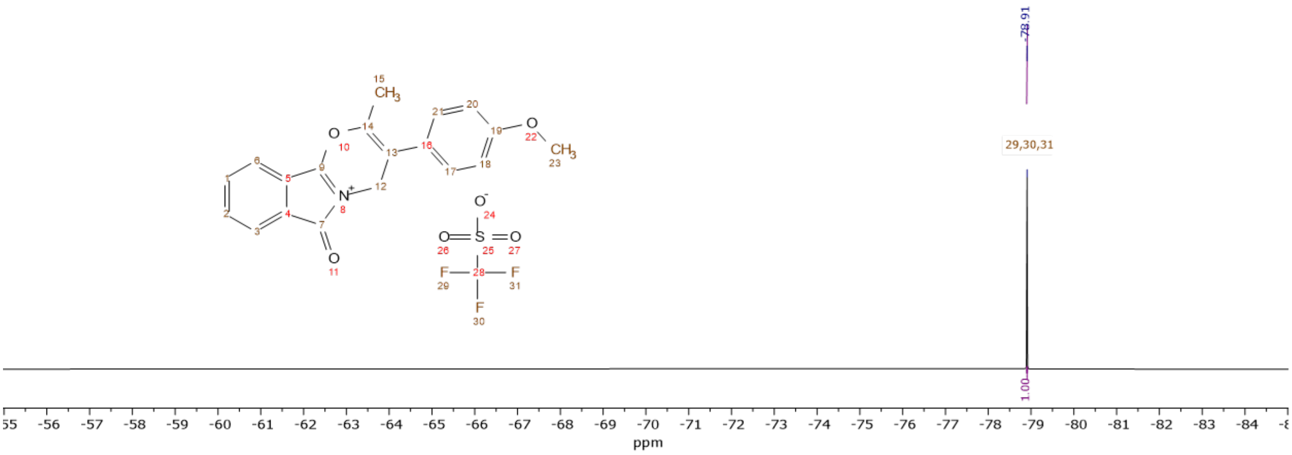

Figure S.34 ^{19}F NMR spectrum of **2g** (CD_2Cl_2, 298K).

2h - 2-(2-(4-methoxyphenyl)-3-oxobutyl)isoindoline-1,3-dione. ^{1}H NMR (400 MHz, CD_2Cl_2) δ 7.74 (ddt, *J* = 20.6, 5.6, 3.1 Hz, 4H, *H1*, *H2*, *H3*, *H6*), 7.15 (m, 2H, *H18*, *H22*), 6.83 (m, 2H, *H19*, *H21*), 4.37 (dd, *J* = 9.4, 6.4 Hz, 1H, *H13*), 4.10 (m, 2H, *H12*), 3.76 (s, 3H, *H24*), 2.07 (s, 3H, *H15*). ^{13}C{^{1}H} NMR (101 MHz, CD_2Cl_2) δ 206.3 (*C14*), 167.9 (*C7*, *C9*), 159.3 (*C20*), 133.9 (*C1*, *C2*), 131.8 (*C4*, *C5*), 129.7 (*C18*, *C22*), 127.4 (*C17*), 123.0 (*C3*, *C6*), 114.3 (*C19*, *C21*), 55.6 (*C13*), 55.1 (*C24*), 39.4 (*C12*), 28.7 (*C15*).

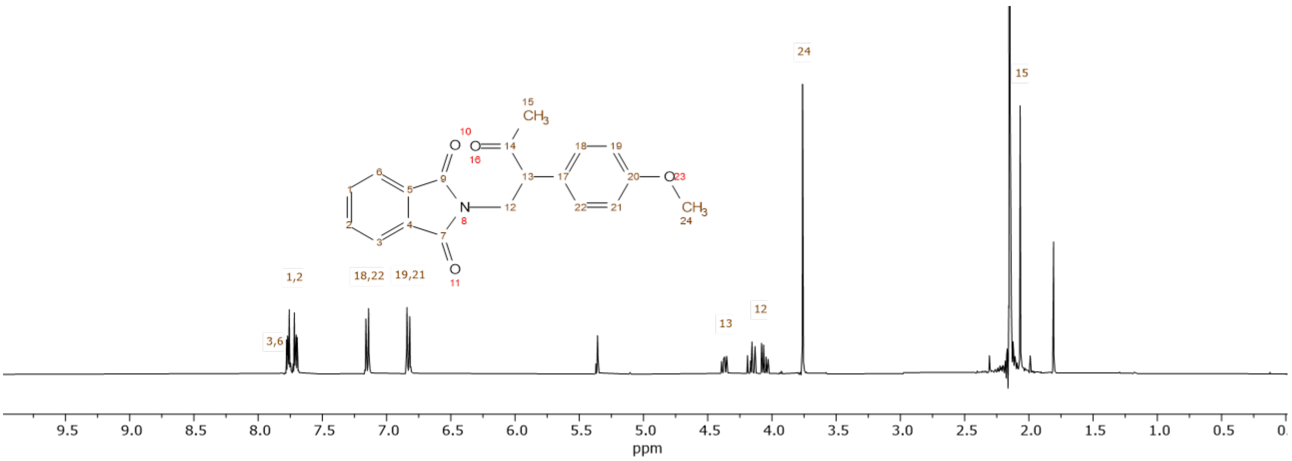

Figure S.35 ^{1}H NMR spectrum of **2h** (CD_2Cl_2, 298K).

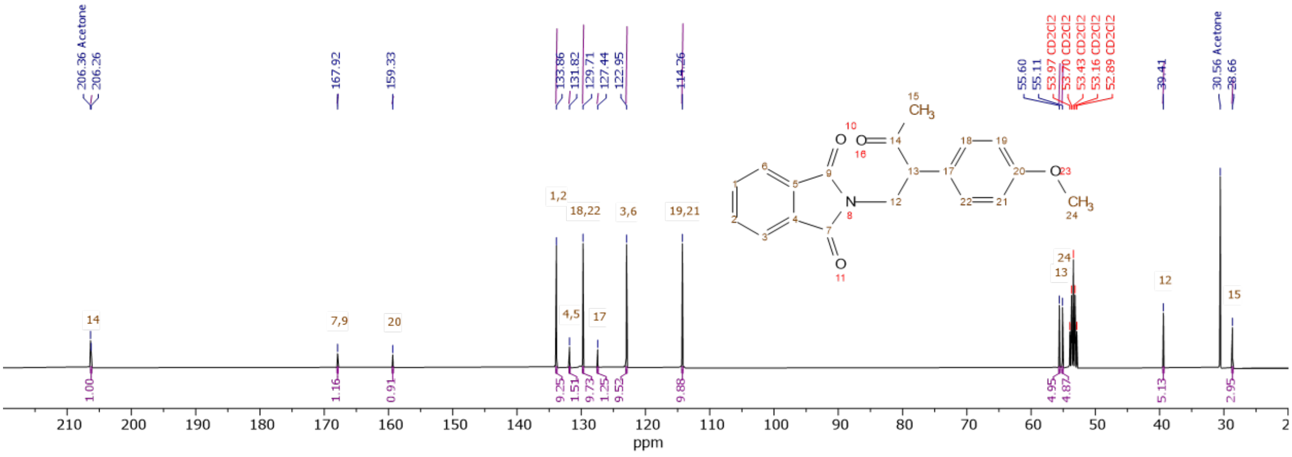

Figure S.36 ^{13}C{^{1}H} NMR spectrum of **2h** (CD_2Cl_2, 298K).

2i - (E)-4-(4-(methyl)phenyl)hex-3-en-3-yl trifluoromethanesulfonate. ^{1}H NMR (400 MHz, CD_2Cl_2) δ 7.24 (d, *J* = 7.8 Hz, 1H, *H10*, *H12*), 7.09 (d, *J* = 8.1 Hz, 1H, *H9*, *H13*), 2.52 (q, *J* = 7.5 Hz, 1H, *H6*), 2.40 (s, 1H, *H14*), 2.28 (q, *J* = 7.4 Hz, 1H, *H4*), 1.06 (t, *J* = 7.4 Hz, 1H, *H8*), 0.92 (t, *J* = 7.5 Hz, 1H, *H7*). ^{13}C{^{1}H} NMR (101 MHz, CD_2Cl_2) δ 147.4 (*C1*), 137.7 (*C2*), 135.9 (*C3*), 134.5 (*C11*), 129.1 (*C10*, *C12*), 128.2 , (*C9*, *C13*), 25.6 (*C6*), 24.5 (*C4*), 11.5 (*C8*), 11.3 (*C7*). ^{19}F NMR (376 MHz, CD_2Cl_2) δ -75.51 (*F19, F20, F21*).

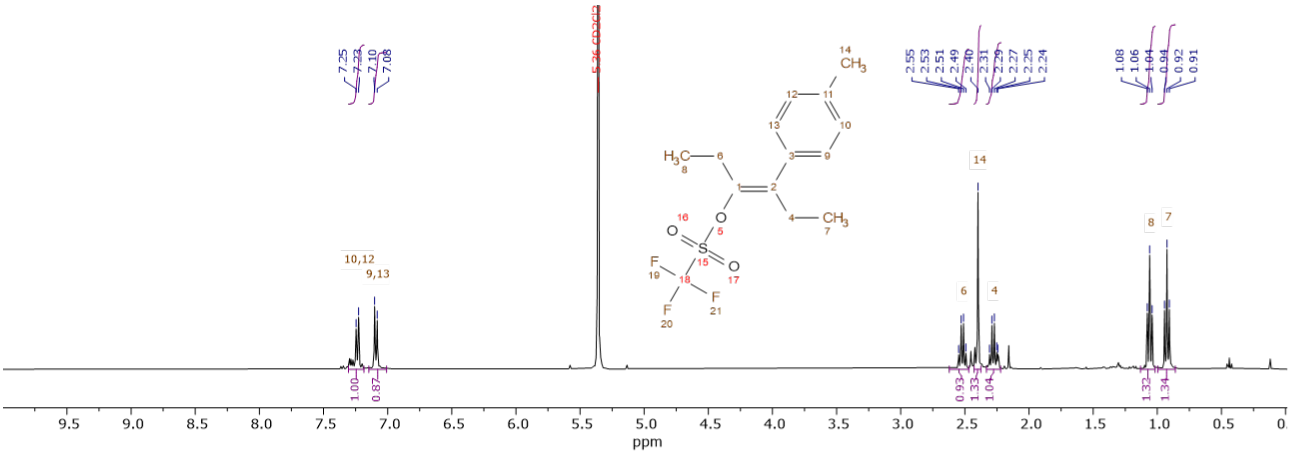

Figure S.37 ^{1}H NMR spectrum of **2i** (CD_2Cl_2, 298K).

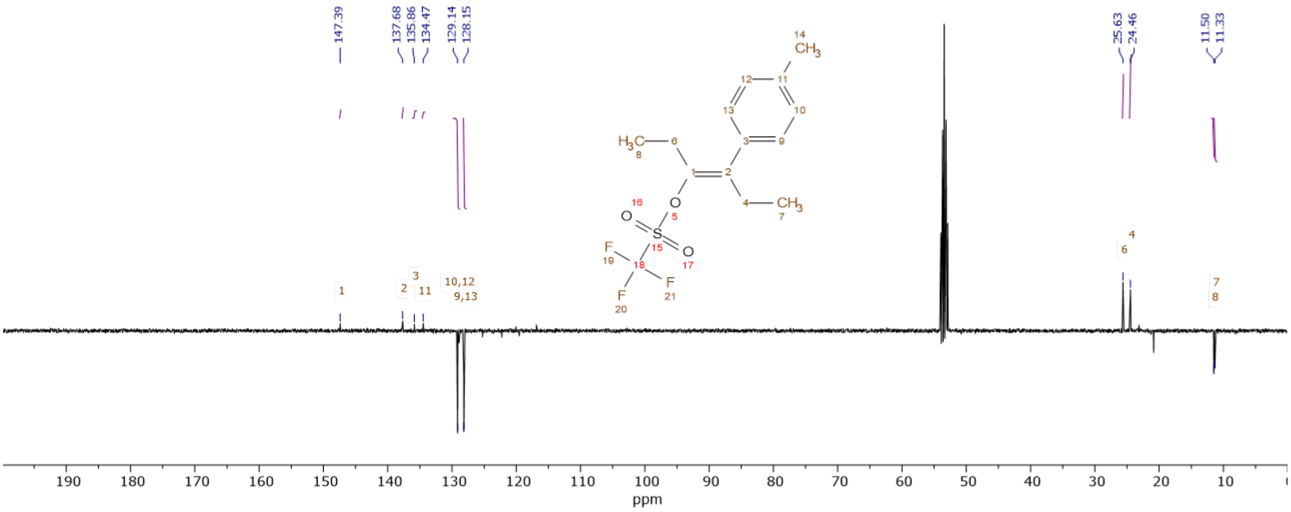

Figure S.38 ^{13}C{^{1}H} Jmod NMR spectrum of **2i** (CD_2Cl_2, 298K).

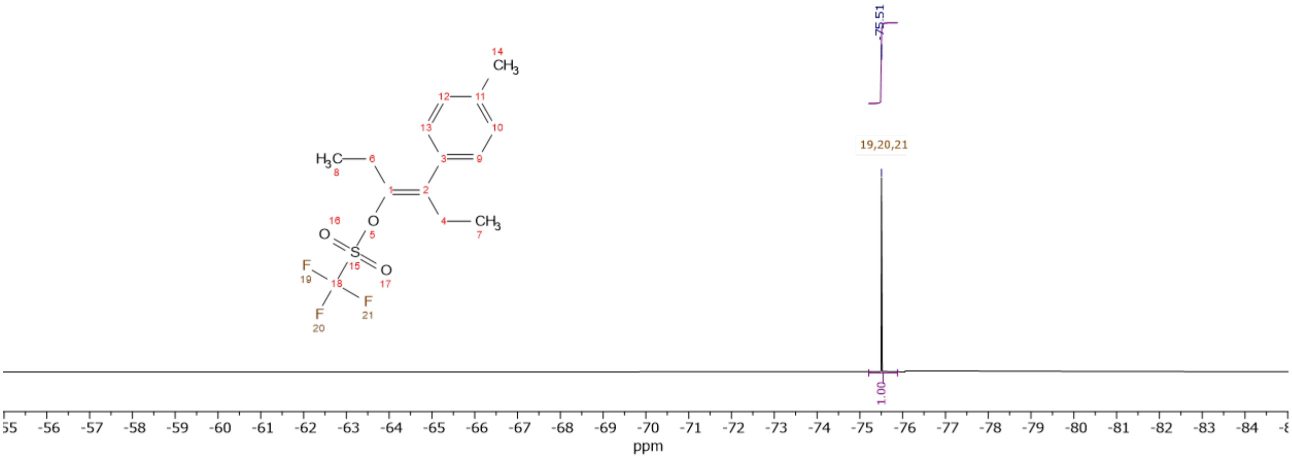

Figure S.39 ^{19}F NMR spectrum of **2i** (CD_2Cl_2, 298K).

2j - (E)-4-(4-(trifluoromethyl)phenyl)hex-3-en-3-yl trifluoromethanesulfonate. ^{1}H NMR (400 MHz, CD_2Cl_2) δ 7.71 (d, *J* = 8.0 Hz, 2H, *H9*, *H13*), 7.36 (d, *J* = 8.0 Hz, 2H, *H10*, *H12*), 2.56 (q, *J* = 7.6 Hz, 2H, *H6*), 2.25 (q, *J* = 7.4 Hz, 2H, *H5*), 1.07 (t, *J* = 7.4 Hz, 3H, *H8*), 0.94 (t, *J* = 7.5 Hz, 3H, *H7*). ^{13}C{^{1}H} NMR (101 MHz, CD_2Cl_2) δ 128.9, (*C9*, *C13*), 125.5 (q, *J* = 3.7 Hz, *C10*, *C12*), 25.5, (*C6*), 24.6, (*C5*), 11.4, (*C8*), 11.2, (*C7*). ^{19}F NMR (376 MHz, CD_2Cl_2) δ -62.97 (*F18, F19, F20*), -75.34 (*F22, F23, F24*).

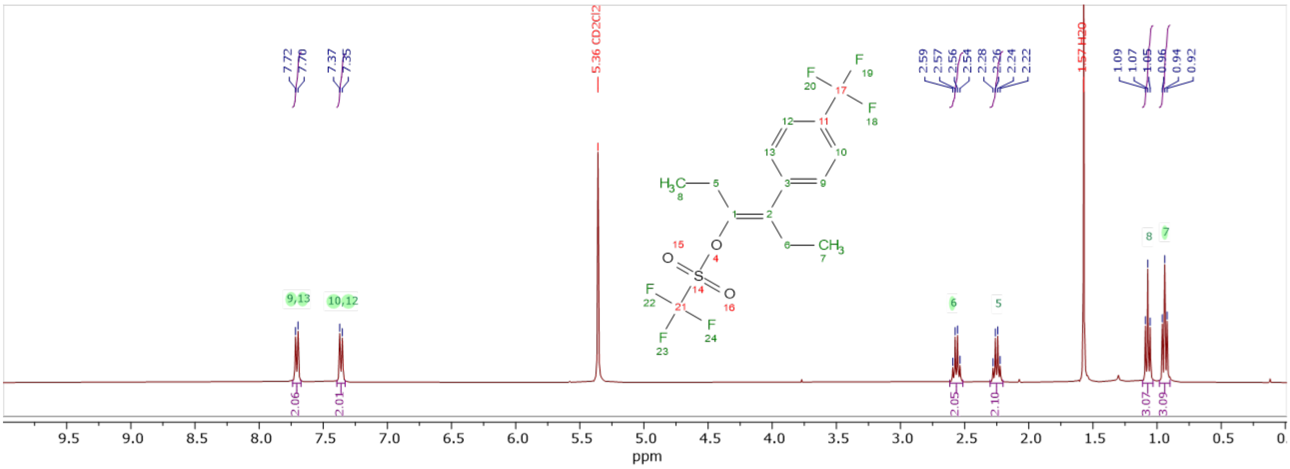

Figure S.40 ^{1}H NMR spectrum of **2j** (CD_2Cl_2, 298K).

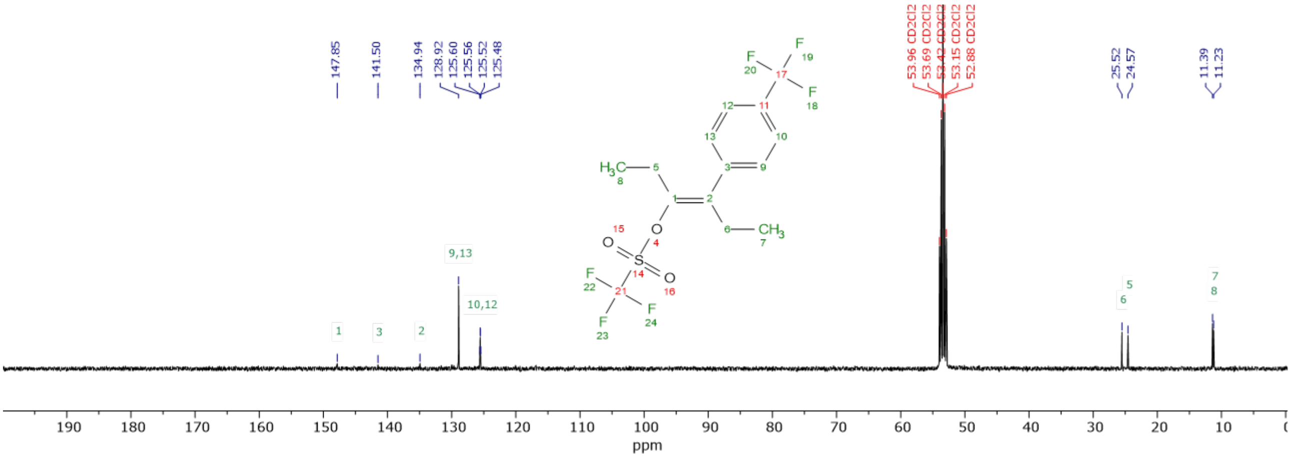

Figure S.41 ^{13}C{^{1}H} NMR spectrum of **2j** (CD_2Cl_2, 298K).

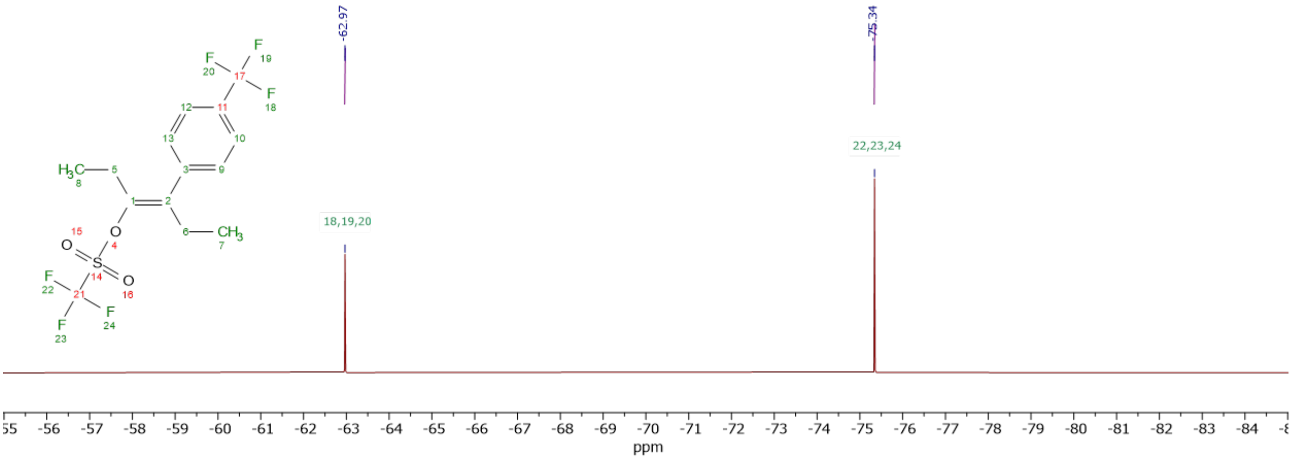

Figure S.42 ^{19}F NMR spectrum of **2j** (CD_2Cl_2, 298K).

2k - (3E,5E)-4-ethyldodeca-3,5-dien-3-yl trifluoromethanesulfonate. ^{1}H NMR (600 MHz, CD_2Cl_2) δ 6.06 (d, *J* = 15.6 Hz, 1H, *H3*), 5.93 (dt, *J* = 15.6, 6.9 Hz, 1H, *H7*), 2.51 (q, *J* = 7.4 Hz, 2H, *H6*), 2.35 (q, *J* = 7.5 Hz, 2H, *H4*), 2.17 (q, *J* = 7.2 Hz, 2H, *H8*), 1.42 (q, *J* = 7.2 Hz, 2H, *H9*), 1.30 (m, 6H, *H10, H11, H12*), 1.11 (t, *J* = 7.4 Hz, 3H, *H15*), 1.04 (t, *J* = 7.5 Hz, 3H, *H14*), 0.89 (dt, *J* = 7.3, 3.7 Hz, 5H, *H13*). ^{13}C{^{1}H} NMR (151 MHz, CD_2Cl_2) δ 148.0 (*C1*), 135.8 (*C7*), 132.1 (*C2*), 123.2 (*C3*), 118.5 (q, *J* = 319.3 Hz, *C19*), 33.5 (*C8*), 31.8 (*C10*), 29.3 (*C9*), 28.9 (*C11*), 23.7 (*C6*), 22.7 (*C12*), 20.4 (*C4*), 13.9 (*C13*), 12.9 (*C14*), 11.6 (*C15*). ^{19}F NMR (565 MHz, CD_2Cl_2) δ -75.62 (*F20, F21, F22*).

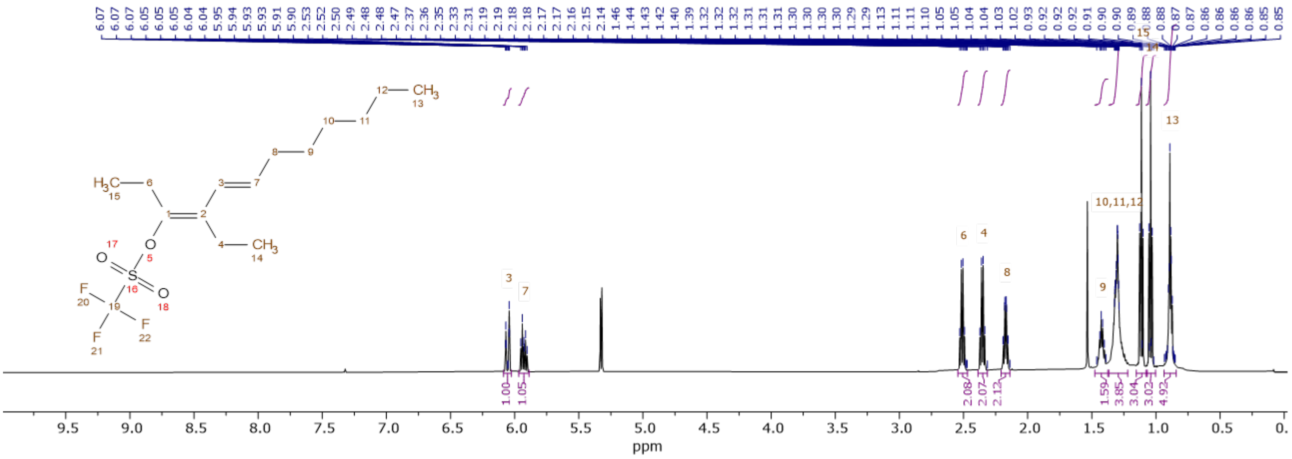

Figure S.43 ^{1}H NMR spectrum of **2k** (CD_2Cl_2, 298K).

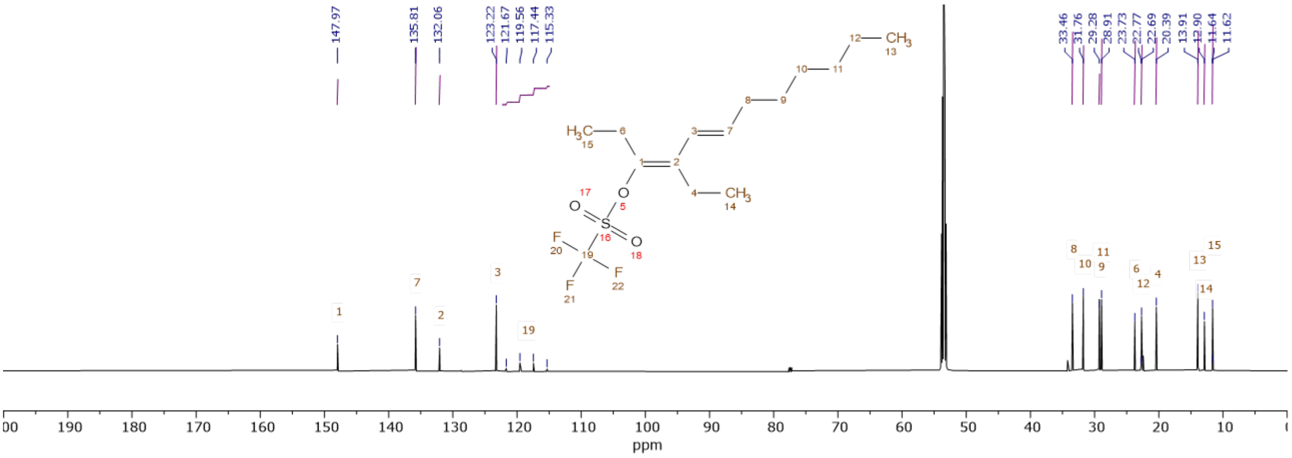

Figure S.44 ^{13}C{^{1}H} NMR spectrum of **2k** (CD_2Cl_2, 298K).

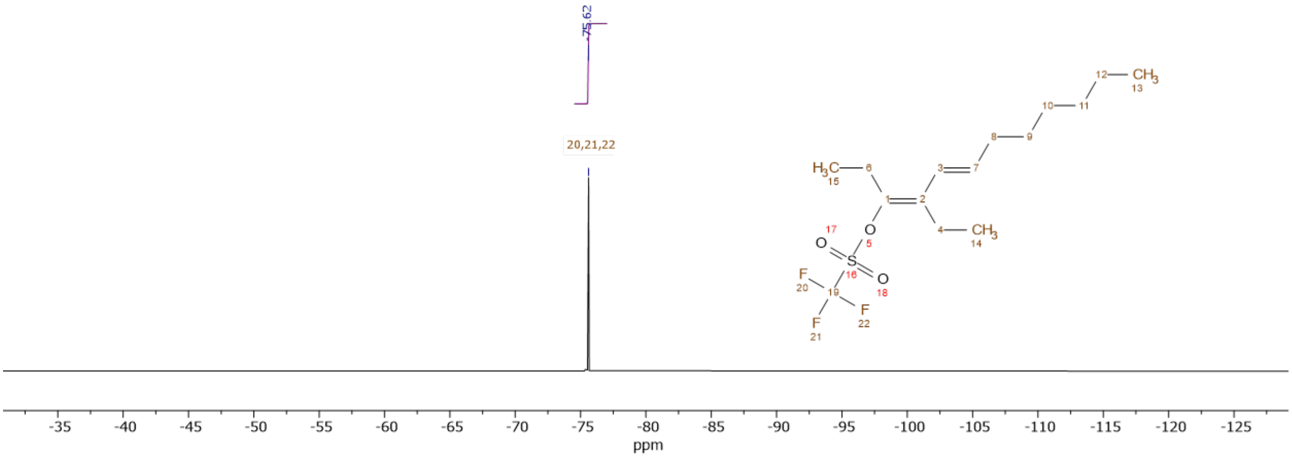

Figure S.45 ^{19}F NMR spectrum of **2k** (CD_2Cl_2, 298K).

Table S.2 Yield and purification conditions for the vinyltriflates obtained in this work

Alkyne	R–I	Products	Physical aspect	Isolated combine yield[a]	Isolation method[b]	Rf	Eluent
3-hexyne	p-iodoanisole	**2a**	Colorless oil	13.9 mg (82%)	Flash column chromatography	0.2	n-pentane
2-hexyne	p-iodoanisole	**2b/2b'**	Colorless oil	13.0 mg (71%)	Flash column chromatography	0.2	n-hexane
1-phenylpropyne	p-iodoanisole	**2c/2c'**	Colorless oil	10.0 mg (54%)	n-pentane extraction		
Methyl phenylpropiolate	p-iodoanisole	**2d**	Colorless oil	15.2 mg (73%)	Flash column chromatography	0.2	8:2 DCM/n-hexane
ethyl but-2-ynoate	p-iodoanisole	**2e**	Colorless oil	10.1 mg (55%)	Flash column chromatography	0.1	gradient[c]
diphenylacetylene	p-iodoanisole	**2f**	Colorless oil	Traces	-		
N-(2-Butynyl)phthalimide	p-iodoanisole	**2g**	Colorless oil	15.4 mg (70%)	n-pentane extraction		
N-(2-Butynyl)phthalimide	p-iodoanisole	**2h**	Colorless oil	4.7 mg (29%)	Flash column chromatography	0.3	DCM
3-hexyne	p-iodotoluene	**2i**	Colorless oil	12.4 (77%)	Flash column chromatography	0.1	n-pentane
3-hexyne	p-iodobenzotrifluoride	**2j**	Colorless oil	12.0 (64%)	Flash column chromatography	0.3	9:1 DCM/n-pentane
3-hexyne	trans-1-iodo-1-octene	**2k**	Colorless oil	10.4 mg (61%)	n-pentane extraction		

[a] All compounds were isolated as oils; yield was determined after solvent removal in a 4 mL vial. [b] Flash column chromatography was performed using silica as stationary phase (mesh 230-400). [c] Compounds **E-** and **Z-2e** were isolated via gradient flash chromatography: n-pentane -> 9:1 DCM/n-pentane, (approx. Rf 0.15 and 0.10, 9:1 DCM/n-pentane).

3. X Ray Crystallography

Single crystals of **1naph** were grown by layering a concentrated solution in CD_2Cl_2 with pentane under an inert atmosphere. One crystal was selected, mounted under oil on a MiTeGen MicroLoop and fixed in a cold nitrogen stream on a Bruker D8 Venture diffractometer (Bruker Instrument Service vV6.2.10), equipped with an Incoatec IμS 3.0 microfocus sealed-tube MoKα radiation source (λ = 0.71073 Å) and a Photon II CCD detector. Data collection employed thin-slice ϕ and ω scans, with integration and reduction carried out using Bruker AXS SAINT v8 software.[S1] The crystal structure was solved using SHELXT and refined anisotropically with SHELXL,[S2] both implemented within the Bruker APEX5 software suite.[S1]

**Complex**	**1naph**
**CCDC**	2465124
Elemental formula	$C_{41.25}H_{49.50}AuCl_{2.50}F_6NO_6PS_2$
Formula weight	1149.88 g/mol
Temperature	150(2) K
Wavelength	0.71073 Å
Crystal system	triclinic
Space group	P -1
Unit cell dimensions	
a (Å)	10.8551(3)
b (Å)	12.0723(5)
c (Å)	16.9680(8)
α (°)	89.1480(10)
β (°)	87.5680(10)
γ (°)	79.507(2)
Volume ($Å^3$)	2184.38(15)
Z	2
Calculated density (g/cm^3)	1.748
F(000)	1149
Absorption Coefficient (mm^{-1})	3.726
Temperature (K)	150(2)
Crystal colour, shape (mm)	orange, plate
Crystal size (mm)	0.050 x 0.300 x 0.400
On the diffractometer:	
Theta range for data collection	1.91 to 27.53°
Limiting indices	-14<=h<=14, -15<=k<=15, -22<=l<=22
Completeness	99.8%
Min. and max. transmission	0.5111 and 0.7456
Reflns collected (not incl. absences)	118700
No of unique reflns, R(int) for equivs	10030, 0.0485
No. of 'observed' reflns (I > 2σI)	9525
Refinement:	
Data/restraints/parameters	10030/35/599
Goodness-of-fit on F^2	1.110
Final R indices ('obsd' data)	R1 = 0.0292, wR2 = 0.0701
Final R indices (all data)	R1 = 0.0321, wR2 = 0.0724
Reflns weighted: 1/w = [a]	$w = 1/[\sigma^2(Fo^2)+(0.0255P)^2+7.0761]$
Largest diff. peak and hole	2.736 and -1.506 $eÅ^{-3}$

[a] where $P=(Fo^2+2F_c^2)^2/3$

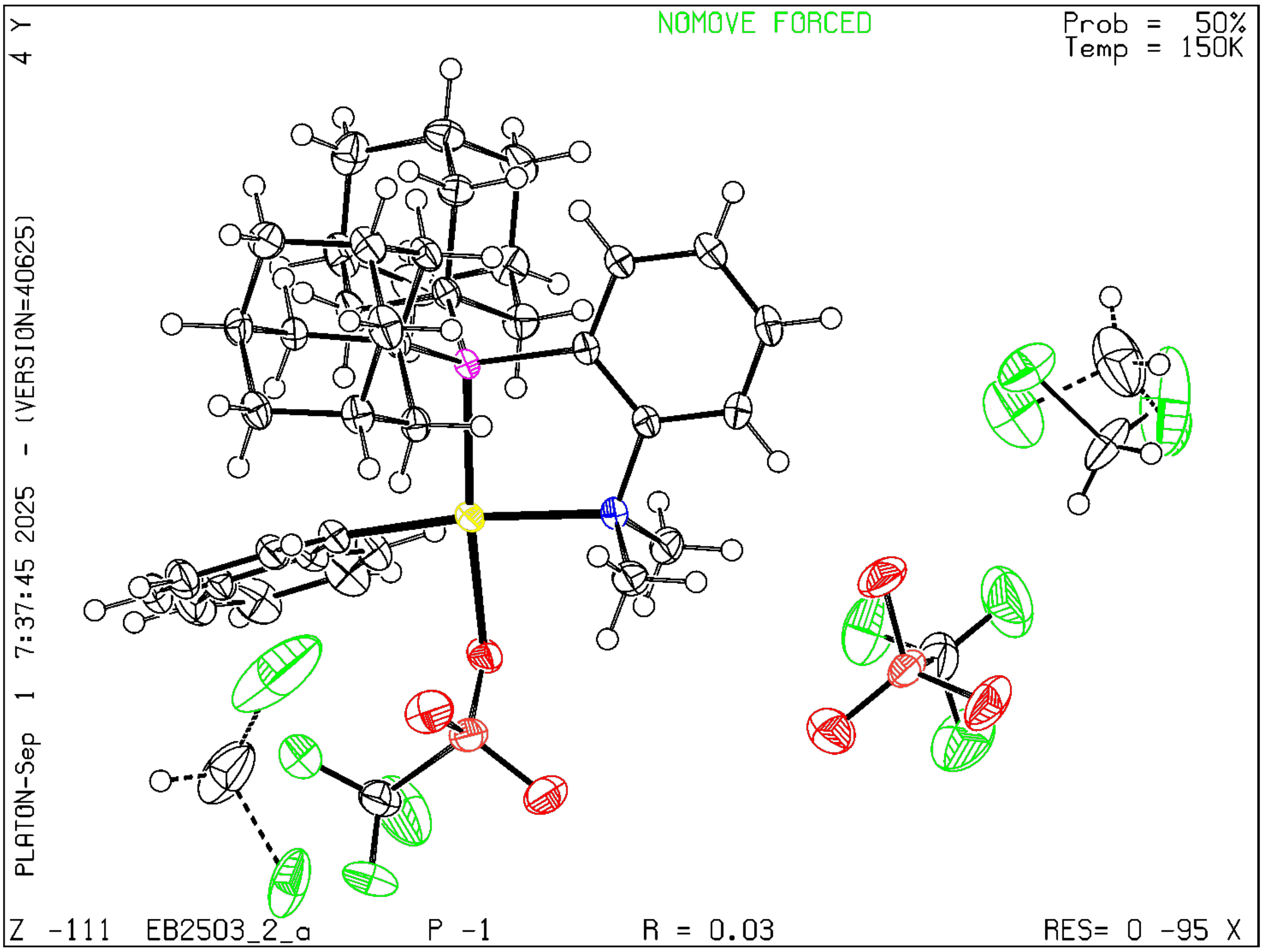

Figure S.46 Ortep plot of **1naph**

4. Details of DFT calculations and additional computational results

DFT static calculations have been performed with Gaussian16 package.[S3] Structures were fully optimized, without symmetry restrictions, using the dispersion-aware MN15 functional,[S4] the cc-pVDZ(pp)[S5] basis set[S6] on all atoms, and the SMD continuum solvation model for dichloroethane.[S7] Improved total energies were obtained from single-point calculations at the optimized geometries, using the cc-pVTZ(pp) basis set,[S8,9] and these energies were combined with thermal corrections (enthalpy and entropy, 333.15 K) to obtain the final Gibbs free energies mentioned in the text. The steric maps and %V_{Bur} reported in the paper have been computed using the web server SambVca 2.1.[S10]

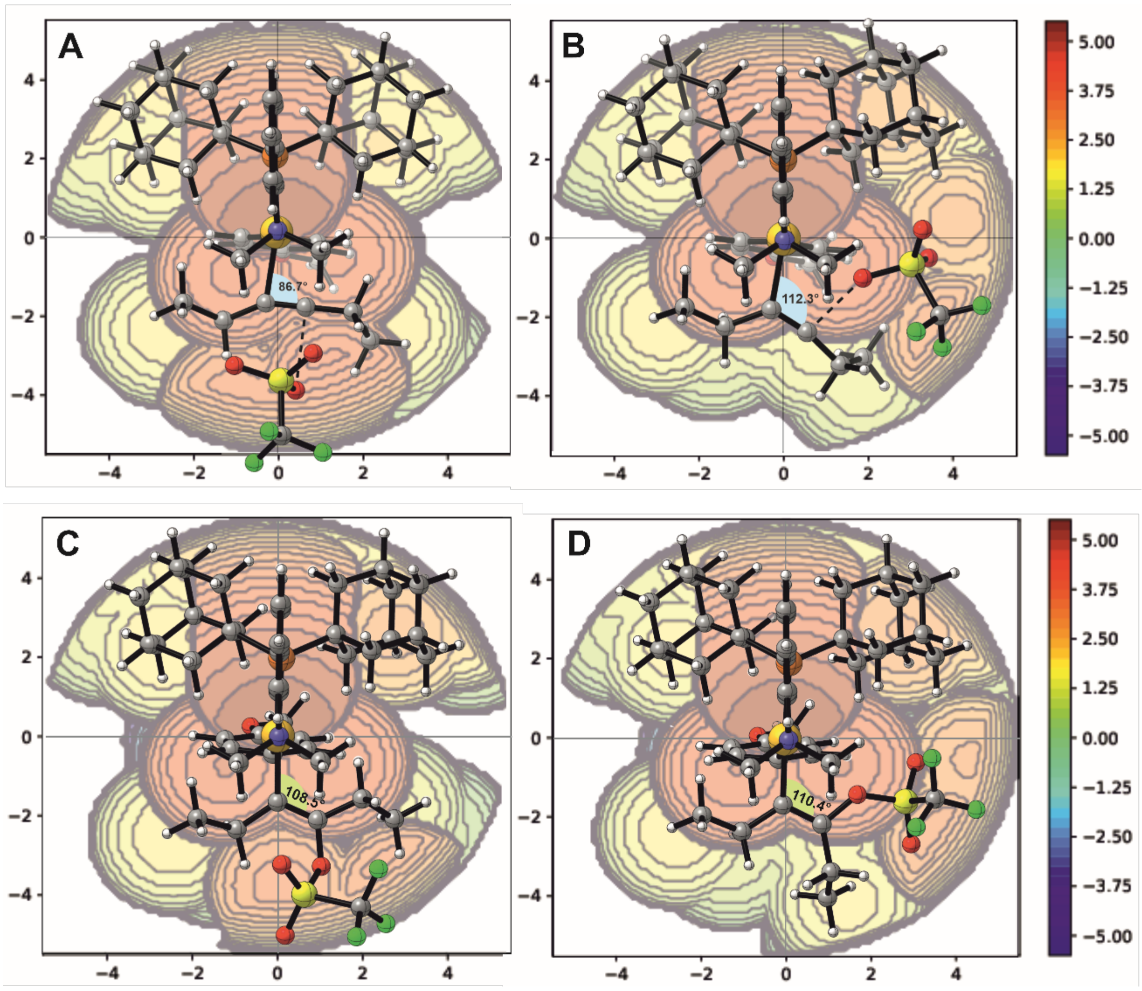

Figure S.47 DFT optimized structures of **TS1-E** (A) and **TS1-Z** (B), **TS2-E** (C), **TS2-Z** (D) with their steric maps.

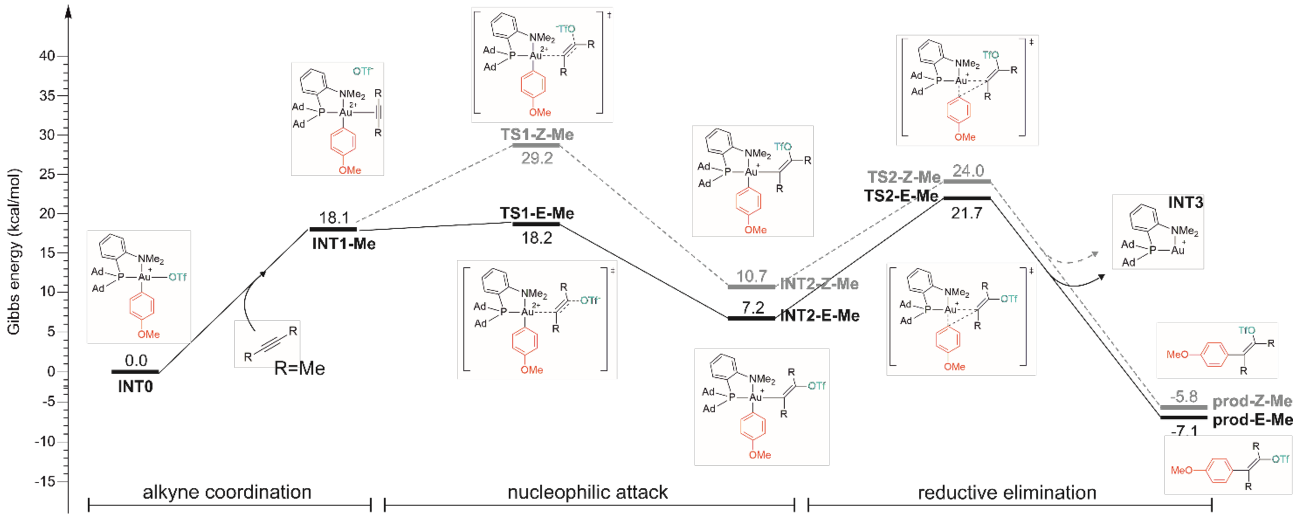

Figure S.48 Gibbs energy profile (kcal/mol) for 2-butyne carbofunctionalization.

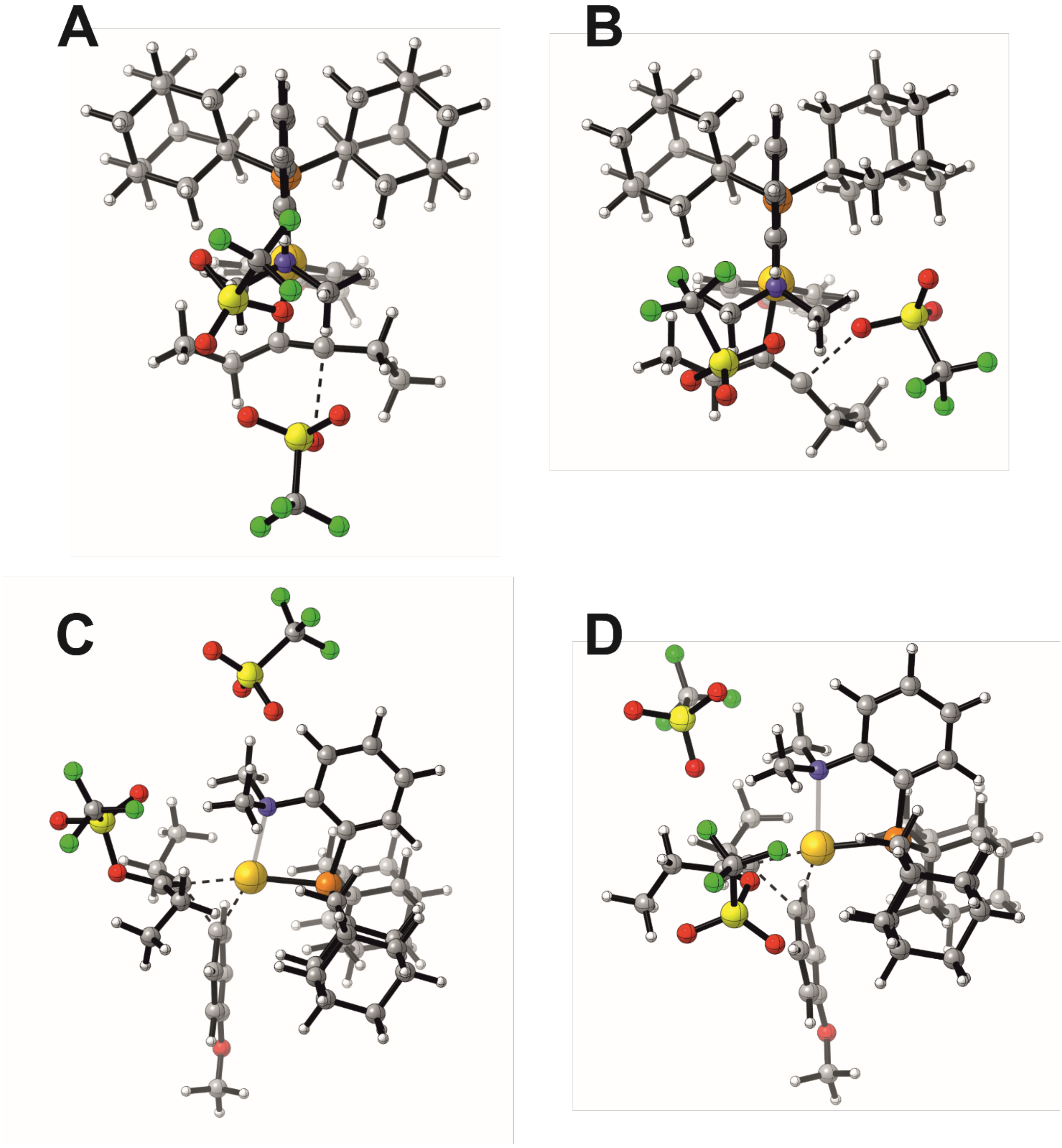

Figure S.49 DFT optimized structures of **TS1-E** (A) and **TS1-Z** (B), **TS2-E** (C), **TS2-Z** (D) in the presence of additional OTf^- molecules. The Gibbs energies between the two RDS is 4.1 kcal/mol.

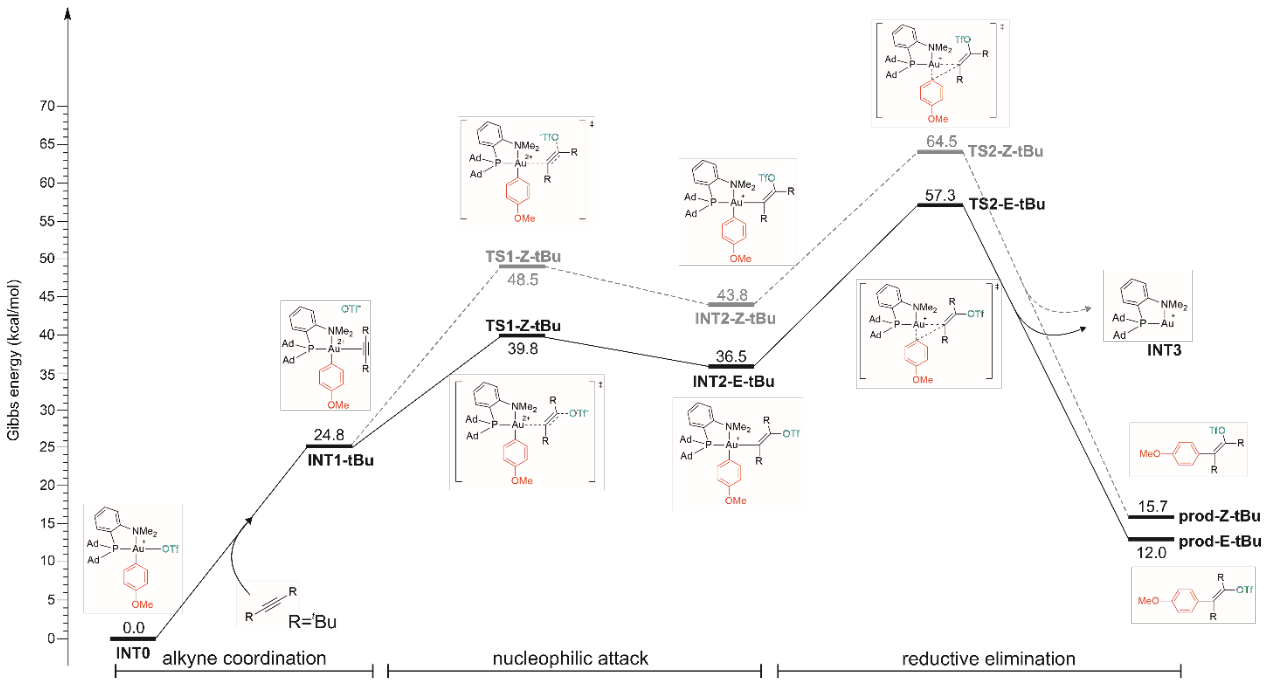

Figure S.50 Gibbs energy profile (kcal/mol) for di-tert-butylacetylene carbofunctionalization.

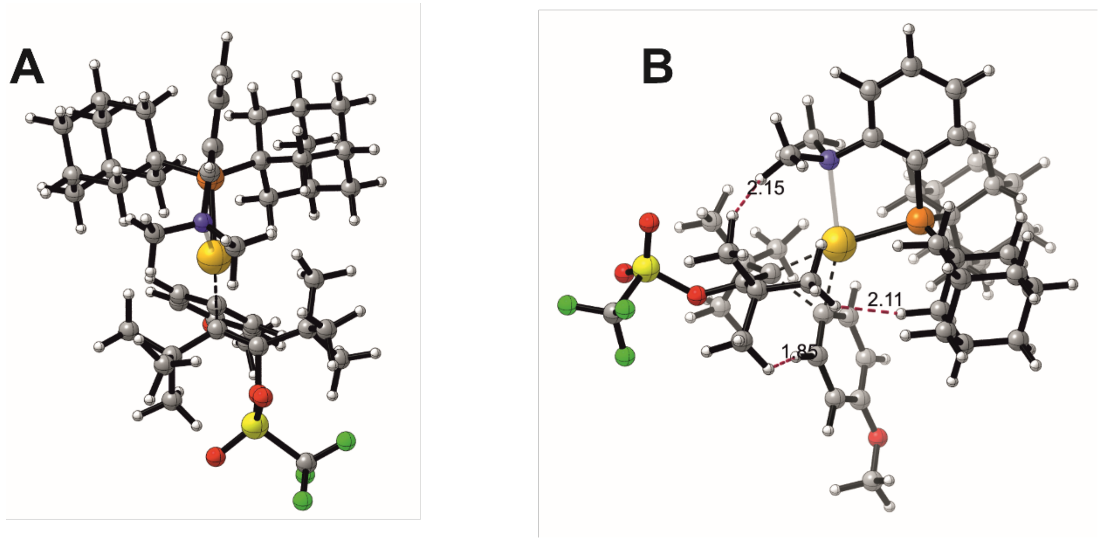

Figure S.51 Optimized structure of the transition state **TS2-E-**^{t}Bu, seen from the top (A) and side (B). Repulsive steric interactions are evidenced in red in B.

Cartesian Coordinates

94

INT0 E(MN15(SMD)/cc-pVTZ(pp))=-2928.47612664

C	-3.88201000	-1.72670400	-0.43364300
H	-4.44945500	-0.96697300	0.10115200
C	-4.55271100	-2.83356100	-0.94384500
H	-5.63256300	-2.91898400	-0.81727500
C	-3.83541800	-3.83008300	-1.60925300
H	-4.34847400	-4.70299000	-2.01519200
C	-2.45600100	-3.71357100	-1.74909300
H	-1.90090100	-4.50013100	-2.26305600
C	-1.77879100	-2.59987400	-1.23795500
C	0.02215500	-2.50804500	-2.85979400
H	-0.26703000	-3.45865000	-3.33319300
H	1.10735200	-2.36852500	-2.94901700
H	-0.50731400	-1.67731100	-3.34754300
C	0.35013900	-3.66632200	-0.74448600
H	0.21225500	-3.59220200	0.34267300
H	1.42060000	-3.63395500	-0.98269200
H	-0.06392600	-4.61869900	-1.10804800
C	-1.72570400	-0.21710500	1.94306900
C	-0.70296500	-1.29478100	2.36840700
H	0.31500000	-1.04109900	2.02334100
H	-0.97993000	-2.25828100	1.89835700
C	-0.69570100	-1.43288000	3.89585300
H	0.04549800	-2.19860300	4.16939600
C	-1.39624700	1.13765100	2.59376600
H	-0.45586300	1.55034600	2.19840000
H	-2.20166300	1.85198700	2.35411400
C	-3.10186100	-0.77682600	3.97638400
H	-4.11001100	-1.07600700	4.30209100
C	-2.72272600	0.56062900	4.61614000
H	-2.72161400	0.46167300	5.71373600
H	-3.46566900	1.33458500	4.35814400
C	-1.33644700	0.97687600	4.11939000
H	-1.05779000	1.94758900	4.55716000
C	-3.11665200	-0.65757800	2.44221000
H	-3.88678700	0.06994900	2.12891300
H	-3.37809700	-1.64150800	2.02332000
C	-0.30366100	-0.08486200	4.50897800
H	-0.25393500	-0.17142800	5.60645700
H	0.70206500	0.21045500	4.16283800
C	-2.08541600	-1.84620800	4.38505700
H	-2.36471300	-2.82325100	3.95519300
H	-2.08094500	-1.96056800	5.48109600
C	-2.25168800	1.38920100	-0.83464400

C	-1.65354800	3.61310400	-2.67512000
H	-1.94995500	4.47193100	-3.29878200
H	-0.55860100	3.50333800	-2.76532900
C	-4.23362500	2.75506100	-1.56901600
H	-5.32553600	2.84978000	-1.46593000
C	-1.58804900	2.69079600	-0.33720800
H	-1.89529800	2.89875100	0.69673900
H	-0.49298800	2.60196600	-0.34884200
C	-2.35442600	2.34332000	-3.16587600
H	-2.08713700	2.14683800	-4.21551700
C	-3.78027200	1.54970100	-0.72669900
H	-4.28652500	0.65701600	-1.12269100
H	-4.07784600	1.68501500	0.32914900
C	-2.03415300	3.86842100	-1.21529100
H	-1.52436100	4.77336400	-0.85131200
C	-3.87218200	2.49959500	-3.03537700
H	-4.38129400	1.59151700	-3.40159200
H	-4.21677900	3.34068000	-3.65836600
C	-1.88448700	1.15460500	-2.31713300
H	-0.78876900	1.04386000	-2.41814200
H	-2.35549200	0.22038200	-2.67587500
C	-3.54951100	4.03547100	-1.08762500
H	-3.89308300	4.89157100	-1.69070600
H	-3.82091900	4.24418400	-0.03876100
Au	0.52147100	-0.65408100	-0.62248000
N	-0.32641100	-2.51879000	-1.41087100
P	-1.60296700	-0.11958800	0.07222500
C	1.43057100	1.07757400	-0.11854600
C	1.85148500	1.37715000	1.17125900
C	1.79119800	1.91006500	-1.18867000
C	2.59343900	2.53995800	1.41724000
C	2.52828900	3.06292100	-0.94900600
C	2.92728800	3.39366400	0.35859700
H	2.92084200	2.75235600	2.43480600
H	2.82793100	3.72519900	-1.76266000
H	1.51552000	1.65870400	-2.21698300
H	1.64849200	0.69707400	2.00012700
O	3.63380100	4.53374700	0.48967600
C	4.10139500	4.88906500	1.77508900
H	3.26493800	5.04448700	2.47715400
H	4.77742700	4.11877400	2.18196000
H	4.65277000	5.82801100	1.65753100
C	-2.48722600	-1.58387400	-0.58111700
O	2.37815500	-1.25970300	-1.34576100
S	3.30249800	-1.89915600	-0.25433700
O	3.72658700	-3.23726300	-0.65491100
O	2.69957600	-1.68250400	1.07296700

C	4.79961000	-0.81587800	-0.39775100
F	5.26124000	-0.89361700	-1.63266800
F	4.48250900	0.43867000	-0.11662000
F	5.70894800	-1.24863500	0.45707900

16

3-hexyne E(MN15(SMD)/cc-pVTZ(pp))=-234.376920833

C	-0.56846200	0.21516500	-0.03029600
C	0.56846300	-0.21516100	-0.03038200
C	1.95594300	-0.69186600	-0.03070500
H	2.13411100	-1.28817000	-0.94074700
H	2.10288300	-1.38082200	0.81737700
C	-1.95594400	0.69186500	-0.03071600
H	-2.10293500	1.38087200	0.81731600
H	-2.13406300	1.28811500	-0.94080300
C	2.96982200	0.45312800	0.04774300
H	2.82386400	1.04045300	0.96578000
H	3.99838000	0.06387400	0.04525800
H	2.85504900	1.13427300	-0.80777100
C	-2.96982200	-0.45313000	0.04774300
H	-3.99838200	-0.06388300	0.04515300
H	-2.85497900	-1.13433300	-0.80771700
H	-2.82392700	-1.04038900	0.96583100

110

INT1 E(MN15(SMD)/cc-pVTZ(pp))=-3162.84750271

C	-1.32450800	-3.25166200	-0.15552700
H	-0.55931200	-4.02087700	-0.06014400
C	-2.65810600	-3.62970400	-0.27510300
H	-2.92085400	-4.68839500	-0.26666200
C	-3.64895600	-2.65296300	-0.40432000
H	-4.70156300	-2.92383800	-0.49213900
C	-3.30686400	-1.30385400	-0.41414900
H	-4.08354500	-0.54250300	-0.51362100
C	-1.96836600	-0.92507700	-0.28322300
C	-2.33471600	1.15157700	0.89874500
H	-3.36292800	0.77019200	1.02516900
H	-2.38797000	2.23312400	0.71835700
H	-1.74876900	0.93569500	1.80652100
C	-2.17746300	1.11801700	-1.54346000
H	-1.71919000	0.60041300	-2.39926900
H	-1.91667900	2.18433400	-1.54915900
H	-3.27753500	1.05221800	-1.55589300
C	1.68252600	-1.94337500	-1.52391100
C	1.13057600	-1.08230700	-2.68246300
H	1.31427900	-0.00982400	-2.47916100
H	0.03685700	-1.22682600	-2.76113000
C	1.81905200	-1.46948300	-3.99775400
H	1.40973600	-0.84009200	-4.80286300

C	3.20576700	-1.76481300	-1.39698000
H	3.46090800	-0.74425700	-1.07415000
H	3.58953300	-2.46031400	-0.63560600
C	2.10701900	-3.80500000	-3.16567800
H	1.89798000	-4.86691400	-3.36551300
C	3.61578100	-3.58293900	-3.04616300
H	4.11085900	-3.86853600	-3.98836800
H	4.03666900	-4.22002800	-2.24997700
C	3.88047800	-2.10816800	-2.73314200
H	4.96226700	-1.93636400	-2.62515900
C	1.39465200	-3.42287900	-1.85660500
H	1.73555800	-4.07481400	-1.03286600
H	0.31489900	-3.57677100	-1.99801100
C	3.32564800	-1.23070800	-3.85840500
H	3.83307300	-1.47155400	-4.80638700
H	3.52613000	-0.16548400	-3.64587000
C	1.54989000	-2.94521900	-4.30317700
H	0.46681100	-3.11842500	-4.41889400
H	2.02928000	-3.22361000	-5.25541900
C	1.35181700	-1.85386900	1.72246300
C	2.42270000	-1.01147000	4.33195100
H	2.71331600	-1.20489100	5.37700900
H	2.64203400	0.05164500	4.12703600
C	1.44597000	-3.64431400	3.49014400
H	1.22169200	-4.70472400	3.68127500
C	2.86082500	-1.62827600	1.92712600
H	3.42584300	-2.32921500	1.29548000
H	3.15362200	-0.60882200	1.63590100
C	0.92887200	-1.29104300	4.14457800
H	0.33607600	-0.64831800	4.81312800
C	1.03332400	-3.32949100	2.04182800
H	-0.04687600	-3.51228000	1.94970700
H	1.56411100	-3.99670900	1.33936200
C	3.23128900	-1.90860700	3.39150100
H	4.30605100	-1.70676100	3.51610700
C	0.63077000	-2.76329800	4.44052400
H	-0.44686400	-2.96448100	4.32068100
H	0.89234200	-2.99455600	5.48554000
C	0.53948700	-0.96997900	2.69568400
H	0.74301400	0.10016500	2.49422700
H	-0.54171900	-1.14200600	2.54184600
C	2.93829400	-3.37967400	3.69603700
H	3.22036300	-3.61799200	4.73427700
H	3.53881800	-4.02942300	3.03698800
Au	0.45779900	0.95396800	-0.08999100
N	-1.65570100	0.51299700	-0.27779200
P	0.76692300	-1.36301800	0.00739300

C	2.41540900	1.49617400	0.10734400
C	3.19473200	1.74997600	-1.02219400
C	2.92214900	1.84792000	1.36835800
C	4.46939200	2.32084900	-0.91064800
C	4.18566500	2.41567600	1.49113300
C	4.97539500	2.65505700	0.35273000
H	5.05197100	2.49929000	-1.81450400
H	4.59291400	2.68724000	2.46649600
H	2.33696200	1.67865800	2.27735200
C	0.00613200	3.25407900	0.46255400
C	0.18337300	3.21754400	-0.76087600
H	2.82606100	1.50723500	-2.02327100
O	6.18386600	3.20203200	0.57359200
C	7.01477600	3.47520200	-0.53856100
H	7.26176900	2.55200400	-1.08860900
H	6.53782000	4.19300400	-1.22631400
H	7.93454000	3.91458200	-0.13878900
C	0.38260000	3.44060500	-2.20551600
H	1.44454500	3.69047400	-2.35983100
H	0.19310200	2.49999100	-2.75148600
C	-0.13578300	3.52717500	1.90362100
H	0.12429100	2.61537500	2.47005200
H	-1.19757100	3.72513800	2.11904800
C	-0.96089700	-1.88807800	-0.15497800
O	-5.05593400	1.39501300	-0.76624000
S	-5.96727300	1.09371700	0.38581000
O	-5.28049000	0.26558800	1.41897400
O	-6.79721000	2.20251700	0.85504400
C	-7.13140800	-0.12627400	-0.36877500
F	-7.80555100	0.40799900	-1.37908000
F	-7.98917900	-0.59214800	0.52968300
F	-6.42540400	-1.17309500	-0.85092100
C	-0.52879900	4.55736200	-2.72724200
H	-0.34217900	4.71754000	-3.79706300
H	-1.58933500	4.30002600	-2.59464300
H	-0.33478500	5.49890100	-2.19601300
C	0.74511100	4.70462000	2.33335300
H	0.61484000	4.89072000	3.40751000
H	1.80550400	4.49356500	2.13441700
H	0.46524800	5.61753200	1.79021700

110

TS1-E E(MN15(SMD)/cc-pVTZ(pp))=-3162.8510226 imaginary frequency=-48.0112

C	-2.42941900	-3.52127900	1.06828500
H	-3.48241500	-3.24870800	1.01920000
C	-2.08305400	-4.81963000	1.42350300
H	-2.86197900	-5.54614600	1.65758400
C	-0.73465500	-5.18188100	1.47087300

H	-0.44461900	-6.19761200	1.74336900
C	0.24816500	-4.24353600	1.17514500
H	1.29700000	-4.53750300	1.23005900
C	-0.09541300	-2.93096000	0.82219200
C	1.91547100	-2.47872100	-0.47430900
H	2.35611300	-3.43462400	-0.15664000
H	2.74414600	-1.76839000	-0.61254500
H	1.35944400	-2.62228900	-1.41167700
C	1.74869300	-1.76802100	1.83893700
H	1.12762500	-1.21517700	2.55923500
H	2.69168600	-1.25028400	1.61045500
H	2.01078600	-2.74561100	2.26958500
C	-2.75078600	-0.04724700	1.69702500
C	-1.65476200	0.08805600	2.77767100
H	-0.80824100	0.68383600	2.38351100
H	-1.26896700	-0.91429000	3.04120300
C	-2.22314000	0.78112700	4.02233600
H	-1.42233200	0.85914400	4.77368600
C	-3.29449400	1.34585700	1.32591700
H	-2.51981700	1.95228800	0.83466200
H	-4.13074000	1.24174700	0.62080900
C	-4.48149400	-0.16243700	3.52103800
H	-5.31220900	-0.77062300	3.91049800
C	-4.98583200	1.23335800	3.14938600
H	-5.40397400	1.72830400	4.04088300
H	-5.79636900	1.16302100	2.40429000
C	-3.82228400	2.05084900	2.58346700
H	-4.17496400	3.05165900	2.29094000
C	-3.90603700	-0.87926200	2.28685000
H	-4.69484800	-1.02995300	1.52791900
H	-3.53846300	-1.86491600	2.60963600
C	-2.71446200	2.17772700	3.63156700
H	-3.09525400	2.70542200	4.52085700
H	-1.87833100	2.77728500	3.22981300
C	-3.38445500	-0.04445400	4.58306200
H	-3.03022600	-1.04624500	4.88001800
H	-3.78672900	0.44092200	5.48681000
C	-2.80250700	-1.05298500	-1.39839000
C	-2.94933800	-0.20789700	-4.21650300
H	-3.36206400	-0.27635700	-5.23589200
H	-2.19288700	0.59626300	-4.22838600
C	-4.45913500	-2.30060100	-2.82708100
H	-5.20961400	-3.10557500	-2.80492900
C	-3.50892100	0.25440700	-1.79980900
H	-4.35770700	0.42599000	-1.11954500
H	-2.82999100	1.11707000	-1.71196900
C	-2.30173200	-1.53943500	-3.82445300

H	-1.49132700	-1.78606400	-4.52757200
C	-3.84510700	-2.18813000	-1.42046900
H	-3.36566500	-3.14946700	-1.18281200
H	-4.63752100	-1.99941500	-0.67439900
C	-4.06660200	0.12823300	-3.22517000
H	-4.53119200	1.08789700	-3.49816900
C	-3.35272300	-2.65222100	-3.82541800
H	-2.88726300	-3.61569300	-3.55682600
H	-3.77852800	-2.76502600	-4.83542600
C	-1.69937200	-1.39975600	-2.42097800
H	-0.93944100	-0.59886000	-2.43319600
H	-1.19278000	-2.33703300	-2.12035900
C	-5.11958700	-0.98260900	-3.23580600
H	-5.56038200	-1.08176200	-4.24095400
H	-5.94050900	-0.73289300	-2.54228200
Au	0.26275400	0.03046100	-0.07534800
N	0.98219000	-1.96513800	0.57133100
P	-1.89892300	-0.87312900	0.24131000
C	-0.31495700	1.91232700	-0.59592600
C	-0.28821900	2.91246700	0.37612800
C	-0.58091300	2.25665200	-1.92679000
C	-0.57609700	4.24158200	0.04597600
C	-0.86841000	3.57626800	-2.26389000
C	-0.87719300	4.58016700	-1.28154600
H	-0.54971400	5.00001800	0.82840100
H	-1.08012500	3.86289400	-3.29518300
H	-0.55438800	1.50412500	-2.71972700
C	2.24197900	0.79851300	-0.73923300
C	2.45298700	1.08341600	0.45964400
H	-0.03932900	2.67841400	1.41614100
O	-1.17073700	5.82329700	-1.70516900
C	-1.14496300	6.87942100	-0.76480600
H	-1.88856600	6.72180400	0.03431800
H	-0.14441200	6.98907200	-0.31488500
H	-1.39534000	7.79214300	-1.31552600
C	2.69996300	1.41689900	1.86294600
H	1.76250600	1.26694000	2.43219800
H	3.45516300	0.70497200	2.23452100
C	2.57842900	0.94555700	-2.17984400
H	2.07344100	1.86538300	-2.51923000
H	3.66459000	1.13090900	-2.17242300
C	-1.44433200	-2.55987700	0.75879600
O	5.05523800	1.19475700	-0.00654900
S	5.17977300	-0.29048900	-0.01010900
O	4.66612200	-0.94303400	1.22840400
O	4.72577300	-0.94325900	-1.26556900
C	6.99839400	-0.59578900	0.04506300

F	7.51224800	-0.05001500	1.14094400
F	7.57688600	-0.06089500	-1.02269800
F	7.23089700	-1.90346300	0.05913400
C	3.20215100	2.86162700	1.98656700
H	4.13702600	2.95467900	1.41938300
H	2.46709100	3.57917200	1.59309300
H	3.39760100	3.09105300	3.04237100
C	2.24401600	-0.23274600	-3.08421300
H	2.87545700	-1.09348300	-2.83035600
H	1.17920500	-0.51842400	-3.01212300
H	2.44440600	0.03938000	-4.12892700

118

TS1-E OTf- E(MN15(SMD)/cc-pVTZ(pp))=-4124.12021854 imaginary frequency= -105.1118

C	0.50536000	-3.95462400	-0.62661000
H	1.50466600	-4.38767800	-0.61076900
C	-0.59297000	-4.77887500	-0.84110400
H	-0.44700600	-5.85017500	-0.98797900
C	-1.87406200	-4.22478300	-0.86152700
H	-2.75593800	-4.84672000	-1.01178800
C	-2.05556900	-2.85677600	-0.67694400
H	-3.06660900	-2.44101300	-0.70393900
C	-0.95488700	-2.02652800	-0.44208600
C	-2.02868700	-0.43948200	1.04098100
H	-2.89870500	-1.11274400	0.99095100
H	-2.37596800	0.60412900	1.09460400
H	-1.41360800	-0.70012600	1.91507900
C	-2.00630700	-0.03765600	-1.33588600
H	-1.43241600	-0.13797500	-2.27019900
H	-2.25883400	1.00949900	-1.12411500
H	-2.96672300	-0.57359500	-1.39287900
C	2.78918500	-1.47600400	-1.68890800
C	1.90880500	-0.76210400	-2.74001800
H	1.66845600	0.26063500	-2.39364800
H	0.95421100	-1.30720200	-2.85788600
C	2.64811700	-0.67572500	-4.08039800
H	1.99490400	-0.16800000	-4.80691000
C	4.11496200	-0.71258900	-1.51379300
H	3.94384300	0.27825300	-1.06826100
H	4.77485600	-1.27155800	-0.83507700
C	3.87289400	-2.78584900	-3.54795600
H	4.10172200	-3.80739100	-3.88960900
C	5.16920400	-1.99284900	-3.37099500
H	5.70677400	-1.93529500	-4.33213600
H	5.83455700	-2.50145000	-2.65233500
C	4.83025200	-0.58771400	-2.86658800
H	5.75594200	-0.01108100	-2.71388600
C	3.11144400	-2.88760100	-2.21595800

H	3.71181200	-3.44348500	-1.47305300
H	2.18098400	-3.44547600	-2.39596400
C	3.93734100	0.12700200	-3.88384200
H	4.46865500	0.22924600	-4.84460500
H	3.70071800	1.14609700	-3.53016600
C	2.98145200	-2.08491500	-4.57715100
H	2.05392800	-2.66105500	-4.73325900
H	3.50054800	-2.02754400	-5.54821800
C	2.53361600	-2.02273500	1.50159500
C	3.40696500	-1.20115500	4.19315300
H	3.78915800	-1.41573600	5.20484200
H	3.27964800	-0.10679400	4.12045200
C	3.24627400	-3.88921400	3.03624900
H	3.36688000	-4.98210900	3.09669000
C	3.91054600	-1.37470300	1.72715600
H	4.63076600	-1.80564900	1.01425300
H	3.87429200	-0.28938900	1.54852700
C	2.06379600	-1.90564400	3.98057400
H	1.33260500	-1.55725100	4.72607600
C	2.69597200	-3.54912800	1.64070600
H	1.72091500	-4.04467700	1.52697100
H	3.37534200	-3.93630000	0.86000800
C	4.41086900	-1.68797400	3.14460300
H	5.37723500	-1.18032500	3.28961500
C	2.24500200	-3.42104200	4.09588800
H	1.27647300	-3.92985200	3.95790600
H	2.61302300	-3.68050200	5.10229200
C	1.53516300	-1.55731600	2.58452700
H	1.38434200	-0.46499300	2.51511300
H	0.55480900	-2.03846300	2.41206900
C	4.59388800	-3.20285900	3.26861000
H	4.97746300	-3.46100000	4.26967400
H	5.33616700	-3.55480900	2.53151700
Au	0.62510900	0.57302900	0.10423500
N	-1.19939300	-0.59260200	-0.20220100
P	1.75256300	-1.48473300	-0.12151500
C	2.27912500	1.73637700	0.37536200
C	2.79609000	2.44818700	-0.70849800
C	2.82100200	1.96820400	1.64720300
C	3.86600800	3.33720300	-0.55265400
C	3.88564300	2.84965700	1.81623100
C	4.42375600	3.53597900	0.71722800
H	4.24127900	3.87361300	-1.42439300
H	4.31505500	3.03637400	2.80193600
H	2.40585900	1.47135200	2.52881600
C	-0.36149900	2.48083200	0.60293100
C	-0.52030100	2.69583600	-0.62089900

H	2.36597500	2.32801600	-1.70781900
O	5.45792900	4.36668000	0.97828000
C	5.99067600	5.11944100	-0.08941800
H	6.40681800	4.46614800	-0.87543100
H	5.22772600	5.77845200	-0.53709200
H	6.79499000	5.73317500	0.33094600
C	-0.58089300	2.92903200	-2.06406500
H	-0.00355100	2.12558000	-2.56231300
H	-1.63697500	2.83425900	-2.36578800
C	-0.39449700	3.01546300	1.99119300
H	0.60753400	3.43796300	2.17696400
H	-1.11278100	3.84737200	1.92964600
C	0.33767700	-2.56969300	-0.41842400
O	-2.17483900	4.59932600	-0.13519700
S	-3.30844300	3.62436200	-0.01791400
O	-3.56181400	2.86184500	-1.26419300
O	-3.28334100	2.82451700	1.23112700
C	-4.77994900	4.72264900	0.16661700
F	-4.88679100	5.51825800	-0.89569600
F	-4.64456500	5.47944300	1.25416100
F	-5.88589500	4.00038100	0.27848100
C	-0.03420700	4.31755500	-2.41593000
H	-0.64873300	5.06794900	-1.90257500
H	1.01299300	4.43440600	-2.09913000
H	-0.10053900	4.47246500	-3.50139200
C	-0.78749600	2.03787900	3.08903000
H	-1.83367300	1.73045700	2.96602300
H	-0.14145800	1.14134900	3.09345400
H	-0.68661700	2.52614300	4.06791300
O	-4.83027400	-1.40999400	-0.62047500
O	-6.60966100	-1.82603800	1.11345500
O	-4.33704600	-2.90808100	1.28490100
C	-5.97000200	-3.74424200	-0.56652700
F	-6.96019600	-3.41184900	-1.38994000
F	-4.95221600	-4.21116500	-1.32497600
F	-6.38198900	-4.74670200	0.20872800
S	-5.39914600	-2.29911300	0.43803100

110

TS1-Z E(MN15(SMD)/cc-pVTZ(pp))=-3162.836481 imaginary frequency= -70.9985

C	-2.47826500	-3.32296800	-0.84780500
H	-3.14077200	-3.28085700	0.01647500
C	-2.51883000	-4.43573700	-1.67953200
H	-3.21939100	-5.24549300	-1.47246000
C	-1.64952000	-4.50955100	-2.77076400
H	-1.66640200	-5.37611200	-3.43317500
C	-0.74455400	-3.48062100	-3.00719900
H	-0.05979400	-3.56156500	-3.85249500

C	-0.69984700	-2.35871000	-2.16741400
C	0.04463600	-0.74865000	-3.79100100
H	0.10021300	-1.53796700	-4.55634100
H	0.81779400	0.00137600	-4.00065600
H	-0.95007500	-0.28286200	-3.81614200
C	1.66285600	-1.90524600	-2.41970100
H	1.85812600	-2.35885300	-1.43949200
H	2.39259100	-1.10374300	-2.58556400
H	1.75265400	-2.65650200	-3.21922500
C	-1.07744300	-1.55135600	1.74962400
C	-0.15812600	-2.76930100	1.54414600
H	0.71244900	-2.49534500	0.92713600
H	-0.70424900	-3.57506900	1.02729200
C	0.31542000	-3.29609700	2.90777700
H	0.98772100	-4.14767500	2.72364400
C	-0.28636700	-0.47801200	2.51942800
H	0.62301800	-0.24198700	1.94269600
H	-0.87165700	0.45356800	2.61796900
C	-1.83698700	-2.54537100	3.92520800
H	-2.72957800	-2.85887300	4.48913100
C	-1.10437200	-1.43736800	4.68716800
H	-0.80483300	-1.80255500	5.68292500
H	-1.77819500	-0.57640200	4.84288000
C	0.13245400	-1.01239800	3.89329600
H	0.66777300	-0.21037900	4.42462800
C	-2.30521200	-2.00066600	2.56530300
H	-3.00383500	-1.16750200	2.73615100
H	-2.85206800	-2.79560500	2.03055700
C	1.06225700	-2.20957800	3.68522200
H	1.38419100	-2.60987400	4.66105800
H	1.96464700	-1.89645700	3.14052000
C	-0.90773300	-3.74425100	3.71252800
H	-1.44529200	-4.54875300	3.18070300
H	-0.59283800	-4.15006300	4.68784900
C	-3.14045900	0.04951000	-0.06390300
C	-4.53927800	2.60561900	-0.51861200
H	-5.45741400	3.20436100	-0.63539300
H	-3.68666000	3.30007000	-0.61285600
C	-5.65917200	-0.06385600	-0.10191500
H	-6.51059600	-0.75498400	-0.00450900
C	-3.23342100	1.12587200	1.03618700
H	-3.24102600	0.65320400	2.03085100
H	-2.35622900	1.78816800	1.00207400
C	-4.48036900	1.53123800	-1.60994100
H	-4.47669000	2.00549800	-2.60407000
C	-4.36555300	-0.88434400	0.03086500
H	-4.33882500	-1.61631000	-0.79160500

H	-4.37207800	-1.43505800	0.98386600
C	-4.52366900	1.93809900	0.85986900
H	-4.55736600	2.70824800	1.64553500
C	-5.68991300	0.60334100	-1.47872500
H	-5.67162900	-0.16248400	-2.27282000
H	-6.62113200	1.18033900	-1.60020800
C	-3.18692700	0.71971200	-1.45209800
H	-2.31715900	1.38637300	-1.56903700
H	-3.12005900	-0.05667000	-2.23878200
C	-5.72631400	1.00159200	0.99465600
H	-6.66538800	1.57190300	0.90600800
H	-5.72549200	0.52520900	1.99005000
Au	0.21341200	0.41128200	-1.01106100
N	0.29508000	-1.31165600	-2.44080700
P	-1.49343600	-0.84468300	0.05569700
C	0.19326400	2.05811000	0.20101300
C	1.15711400	2.18221300	1.20213900
C	-0.65924700	3.13684900	-0.06894800
C	1.22632500	3.34815200	1.97426700
C	-0.59316600	4.29887700	0.69511700
C	0.34391700	4.41052200	1.73460200
H	1.98505600	3.41031600	2.75464200
H	-1.25916100	5.14189500	0.50305200
H	-1.38651700	3.08891800	-0.88345000
C	1.48512600	1.73262700	-2.16321300
C	2.71592500	1.58494700	-1.88305200
H	1.88169500	1.38752900	1.39236300
O	0.32935100	5.56654900	2.42881300
C	1.25989900	5.72705400	3.48070200
H	1.12199300	4.95785700	4.25871200
H	2.29725700	5.68202800	3.10914700
H	1.07397800	6.71614400	3.91281900
C	4.12863800	1.78957500	-1.65690300
H	4.69670900	0.87204400	-1.86426600
H	4.37535000	2.48491200	-2.49074900
C	0.89716400	2.89572100	-2.96066300
H	0.65412900	3.65198900	-2.19720700
H	1.68938500	3.33069200	-3.58830800
C	-1.58174200	-2.26219500	-1.08728500
O	2.69325500	-0.17570900	-0.32900200
S	3.40388000	-1.03817900	0.69577900
O	3.63789500	-0.32922000	1.96450000
O	2.86687300	-2.41124700	0.74625800
C	5.06499500	-1.28961400	-0.09124100
F	5.82548500	-0.20209400	0.03210100
F	4.88714700	-1.52665600	-1.39949900
F	5.68212300	-2.31773400	0.45475100

C	-0.33819900	2.60612800	-3.80049400
H	-0.11625600	1.97160500	-4.66943800
H	-1.12184600	2.11267000	-3.20247900
H	-0.74922800	3.55123000	-4.18201200
C	4.47457900	2.42729600	-0.30995200
H	3.89633000	3.35009200	-0.15886100
H	4.25451700	1.73339500	0.51176300
H	5.54540100	2.66820000	-0.28258000

118

TS1-Z OTf- E(MN15(SMD)/cc-pVTZ(pp))=-4124.11147472 imaginary frequency= -86.9998

C	1.50774600	-2.50167100	2.49925100
H	0.76641100	-3.19427800	2.89775900
C	2.79762100	-2.51025600	3.01716400
H	3.06122700	-3.21849700	3.80426000
C	3.74508900	-1.60886800	2.52300600
H	4.77000200	-1.61044300	2.89687900
C	3.39678800	-0.69056200	1.53827000
C	2.09717400	-0.68596400	1.01448500
C	2.65179600	0.02614700	-1.20598000
H	3.70716600	0.04031000	-0.91508200
H	2.50295800	0.82988100	-1.93793200
H	2.38799500	-0.94893600	-1.63965700
C	2.08552700	1.66995300	0.47379700
H	1.52490300	1.85441800	1.39969200
H	1.76210800	2.39134800	-0.28569400
H	3.17408100	1.75741200	0.60860600
C	-1.67789000	-1.14229100	2.25944200
C	-0.92447200	-0.16707800	3.18257900
H	-0.56934100	0.70563200	2.61135300
H	-0.04400100	-0.66458100	3.62060300
C	-1.84986200	0.29256400	4.31896400
H	-1.28954600	1.00782200	4.93988200
C	-2.91924900	-0.41347800	1.71395800
H	-2.58099900	0.50424800	1.20621400
H	-3.45136600	-1.02908400	0.96776400
C	-3.00662000	-1.92023900	4.24186500
H	-3.29111200	-2.81333600	4.82132500
C	-4.25929000	-1.25840000	3.66131000
H	-4.94519500	-0.97263800	4.47614200
H	-4.79738800	-1.97350900	3.01363200
C	-3.85115100	-0.01770800	2.86433400
H	-4.74237700	0.46618200	2.43470600
C	-2.09704300	-2.37152100	3.08786000
H	-2.64018400	-3.10494500	2.47118300
H	-1.20710200	-2.87310700	3.50332200
C	-3.10903500	0.96747400	3.76926300
H	-3.75906500	1.27249400	4.60728500

H	-2.84533400	1.87426200	3.20613100
C	-2.25681600	-0.93296700	5.14280800
H	-1.36461100	-1.41994600	5.57374600
H	-2.90429600	-0.62956300	5.98267000
C	-0.87262100	-3.17552700	-0.03018900
C	-1.75371800	-4.56392600	-2.47886800
H	-1.94040700	-5.47941000	-3.06499000
H	-2.04189200	-3.70788600	-3.11298800
C	-0.73360400	-5.69297000	0.01934500
H	-0.44982100	-6.54776600	0.65328300
C	-2.36353800	-3.29360800	-0.40034300
H	-2.97914100	-3.32235200	0.51314200
H	-2.68584100	-2.41526900	-0.97903800
C	-0.26923600	-4.48016800	-2.10733800
H	0.34572800	-4.45475300	-3.02071000
C	-0.45717400	-4.40290900	0.80758000
H	0.62088600	-4.35361600	1.02469500
H	-0.99923400	-4.43022100	1.76578100
C	-2.60165700	-4.57907600	-1.20314100
H	-3.66933600	-4.63111200	-1.46777700
C	0.11209000	-5.69343000	-1.25644200
H	1.18449500	-5.65683300	-1.00111900
H	-0.06061000	-6.62212900	-1.82566600
C	-0.01453400	-3.19288800	-1.31245300
H	-0.26111800	-2.31817900	-1.93595600
H	1.05470400	-3.11229600	-1.04048300
C	-2.21781300	-5.78524100	-0.34258200
H	-2.41297100	-6.72198700	-0.89104700
H	-2.83290400	-5.80631300	0.57384200
Au	-0.28747300	0.19216400	-0.77345400
N	1.77018100	0.29471200	-0.03113000
P	-0.54201100	-1.53556400	0.81835400
C	-2.16065200	0.16466200	-1.60427500
C	-3.10467100	1.12255400	-1.22429800
C	-2.46425300	-0.68132300	-2.68115400
C	-4.35211500	1.19112400	-1.85652000
C	-3.70230100	-0.61984000	-3.31723900
C	-4.66417700	0.31089300	-2.90021600
H	-5.06283700	1.94805700	-1.52364800
H	-3.94611600	-1.28349200	-4.14889100
H	-1.72859000	-1.40125600	-3.04952500
C	0.01606100	1.53448900	-2.41911800
C	-0.15513500	2.76076700	-2.12427900
H	-2.88115200	1.84862700	-0.43923100
O	-5.84399200	0.29558200	-3.56616000
C	-6.83114900	1.22546600	-3.18010400
H	-7.12664400	1.08692800	-2.12612100

H	-6.48491100	2.26400300	-3.31830100
H	-7.69939600	1.04694600	-3.82451100
C	-0.43602400	4.17657500	-2.20796700
H	0.19281000	4.74375000	-1.50789400
H	-0.03326600	4.41046700	-3.21872500
C	0.12495800	1.02232600	-3.85671700
H	-0.91608300	0.79552900	-4.13930000
H	0.45375100	1.84957400	-4.50434800
C	1.14711300	-1.60173700	1.47566400
O	-0.66858400	2.69727000	0.12015700
S	-1.15055300	3.42046100	1.36060900
O	-2.60113900	3.68572500	1.32982600
O	-0.57025300	2.88323500	2.60370100
C	-0.31826900	5.07380100	1.21826100
F	-0.95323400	5.86041400	0.34484400
F	0.93492100	4.90047500	0.78666500
F	-0.29381600	5.67433000	2.39429400
C	0.99405300	-0.20428500	-4.09312300
H	2.06373700	0.00449400	-3.96234000
H	0.71851900	-1.02410700	-3.40962200
H	0.84620300	-0.55774800	-5.12363000
C	-1.92070900	4.53282700	-2.12277800
H	-2.49672400	3.96286600	-2.86634300
H	-2.31442500	4.30430500	-1.12346500
H	-2.05598800	5.60629500	-2.31146500
C	6.56582600	-0.77888100	-0.18024100
H	4.14549800	0.02019900	1.16932500
F	6.73802700	-0.80984100	1.15118700
F	5.51247500	-1.57572200	-0.45270800
F	7.64004800	-1.31280300	-0.75130000
O	7.54430800	1.60221400	-0.76230000
O	5.26219800	1.41590100	0.26207100
O	5.60115100	0.70736300	-2.09728800
S	6.23178300	0.95042600	-0.77949400

110

INT2-E E(MN15(SMD)/cc-pVTZ(pp))=-3162.869802

C	-2.91009300	-3.43812800	-0.37941700
H	-3.88453000	-3.06622200	-0.06605500
C	-2.77541600	-4.78323700	-0.70476900
H	-3.63832500	-5.44774100	-0.64559700
C	-1.52955600	-5.27080700	-1.10570100
H	-1.40474800	-6.32225000	-1.36838700
C	-0.43589600	-4.41339500	-1.16049100
H	0.53417000	-4.81429800	-1.45801800
C	-0.56663000	-3.05713700	-0.82760900
C	1.27909200	-2.26127000	-2.18808600
H	1.52804000	-3.29697300	-2.46384500

H	2.21138500	-1.68359400	-2.13926600
H	0.59608200	-1.83512500	-2.93545300
C	1.61424300	-2.73239100	0.14641300
H	1.19625300	-2.63445500	1.15735000
H	2.54483300	-2.16206800	0.04557900
H	1.83257100	-3.79268400	-0.05381300
C	-2.44949200	-0.77405700	1.82943200
C	-1.55850800	-1.81516300	2.53973600
H	-0.49382300	-1.58003300	2.33576500
H	-1.75904900	-2.82649000	2.14863800
C	-1.81945400	-1.78971000	4.05172700
H	-1.17846900	-2.54690000	4.52944000
C	-2.10029200	0.60685600	2.41580500
H	-1.02994700	0.79639200	2.24470500
H	-2.65464600	1.41075000	1.90604200
C	-4.17911000	-1.07458500	3.62820000
H	-5.23923200	-1.31173100	3.80673800
C	-3.85886300	0.31715200	4.17977100
H	-4.06971100	0.35205700	5.26109900
H	-4.50119800	1.07329800	3.69662200
C	-2.38393300	0.63564900	3.92207800
H	-2.14649200	1.64197700	4.30084800
C	-3.92769700	-1.09305700	2.11257500
H	-4.57806300	-0.34728300	1.62627300
H	-4.19392900	-2.08566100	1.71436100
C	-1.49815600	-0.40309900	4.61400700
H	-1.67093600	-0.38884700	5.70256100
H	-0.43276600	-0.16257900	4.44760400
C	-3.29290800	-2.12144300	4.30937700
H	-3.52837400	-3.12808500	3.92319300
H	-3.49050500	-2.13342400	5.39364900
C	-3.20039100	-0.02472200	-1.16649000
C	-3.67549400	2.19824700	-3.04268200
H	-4.29057400	2.73152600	-3.78585100
H	-2.74849700	2.78190100	-2.90773600
C	-5.38802900	-0.09377500	-2.41310000
H	-6.31265700	-0.67763100	-2.54105000
C	-3.56233000	1.38832400	-0.66916000
H	-4.12808600	1.31442200	0.27522900
H	-2.64797800	1.97017900	-0.46468600
C	-3.35092700	0.78963400	-3.55192500
H	-2.80078600	0.85240700	-4.50372400
C	-4.49461000	-0.82681400	-1.39876000
H	-4.24860700	-1.81765900	-1.81218500
H	-5.04208900	-0.97496200	-0.45322600
C	-4.43041400	2.10212600	-1.71354500
H	-4.65963300	3.11299800	-1.34294200

C	-4.64916000	0.00347900	-3.75051000
H	-4.42787100	-1.00608700	-4.13680700
H	-5.28356800	0.50853500	-4.49713400
C	-2.46697300	0.07381000	-2.52202500
H	-1.52301200	0.63194400	-2.39630100
H	-2.20788700	-0.94296000	-2.87518200
C	-5.72607500	1.31052200	-1.90666800
H	-6.38041300	1.82233600	-2.63116700
H	-6.27830400	1.25011600	-0.95300400
Au	0.28328300	-0.02996200	-0.39388500
N	0.63813500	-2.21197500	-0.84807100
P	-1.96908900	-0.79301200	0.01633700
C	0.11087500	1.97202000	-0.06915700
C	0.42668200	2.53759400	1.16581200
C	-0.26751600	2.82255900	-1.11899700
C	0.29922400	3.91438800	1.38646200
C	-0.40000200	4.19189200	-0.91062400
C	-0.12821800	4.75149800	0.34791400
H	0.54835800	4.31836300	2.36776800
H	-0.70295600	4.85991600	-1.71879600
H	-0.45260400	2.42849700	-2.12143600
C	2.22081800	0.56320000	-0.77894000
C	3.13017000	0.36728800	0.18845500
H	0.78461100	1.91311800	1.98810900
O	-0.28961400	6.08686300	0.45538600
C	0.01316400	6.69918100	1.69150200
H	-0.62939200	6.31130300	2.50004300
H	1.07045800	6.54723800	1.96629400
H	-0.17566700	7.77052100	1.56400900
C	2.97102200	-0.08373100	1.61112700
H	1.89541600	-0.23599100	1.81699500
H	3.46509400	-1.06304800	1.73997800
C	2.53488500	1.29378000	-2.06618100
H	2.25955000	2.34647900	-1.87399700
H	3.62164400	1.30398600	-2.23902200
C	-1.81654900	-2.55095000	-0.44675300
O	4.48557900	0.71047300	-0.09257000
S	5.38547800	-0.41597300	-0.84401800
O	4.60341500	-1.64350700	-0.98179200
O	6.07694800	0.20785600	-1.95388500
C	6.60179100	-0.67597300	0.53189700
F	5.96771400	-1.24934600	1.54726300
F	7.08315300	0.49191800	0.90986400
F	7.56888600	-1.45967400	0.10298100
C	3.58218000	0.91511800	2.59679300
H	4.66186100	1.01735200	2.41894300
H	3.13211300	1.91286800	2.48398500

H	3.43839100	0.57725900	3.63229900
C	1.81815900	0.83283600	-3.33388100
H	2.23331100	-0.10327000	-3.73261700
H	0.73698000	0.67767500	-3.16260200
H	1.92049000	1.59240000	-4.12136800

110

INT2-Z E(MN15(SMD)/cc-pVTZ(pp))=-3162.868068

C	-2.99611300	-3.18813800	-0.59909500
H	-3.63220300	-3.04352400	0.27277100
C	-3.16590000	-4.32956400	-1.37568700
H	-3.93172900	-5.05968000	-1.11085600
C	-2.34818400	-4.53142800	-2.48944400
H	-2.46927900	-5.41920800	-3.11172600
C	-1.36289700	-3.60028700	-2.80220100
H	-0.72154800	-3.77886400	-3.66676900
C	-1.18481900	-2.45343400	-2.01616300
C	-0.33177300	-0.97737400	-3.72696100
H	-0.43438500	-1.79029500	-4.46229300
H	0.53142700	-0.35641200	-3.99519000
H	-1.24556600	-0.36603100	-3.72440600
C	1.19856800	-2.25297900	-2.36220200
H	1.36319800	-2.69689300	-1.37206300
H	2.00004800	-1.54052500	-2.59823600
H	1.20066600	-3.04720000	-3.12433100
C	-1.12857900	-1.41806600	1.77712000
C	-0.07126600	-2.48448700	1.42501100
H	0.70134600	-2.03472500	0.77376400
H	-0.53596200	-3.32051900	0.87557500
C	0.58364800	-3.02474900	2.70070200
H	1.33190100	-3.77750700	2.40939300
C	-0.41053500	-0.28850600	2.53982600
H	0.38619400	0.11573000	1.89835000
H	-1.09878600	0.54024400	2.77004300
C	-1.54437300	-2.59847800	3.94980800
H	-2.32753700	-3.05174600	4.57714600
C	-0.86786000	-1.45541800	4.71099900
H	-0.42273500	-1.83722900	5.64442800
H	-1.61423700	-0.69257000	4.99232600
C	0.21681500	-0.83214800	3.82841100
H	0.69608700	0.00523400	4.35949200
C	-2.20581900	-2.04320700	2.67707800
H	-2.95883600	-1.28628900	2.95625900
H	-2.72381900	-2.86226300	2.15150200
C	1.26470500	-1.88490800	3.46069200
H	1.74873700	-2.27575300	4.37096500
H	2.05352100	-1.43636100	2.83143200
C	-0.50150400	-3.65846200	3.57777200

H	-0.98365100	-4.49346100	3.04061400
H	-0.05191900	-4.07579800	4.49369700
C	-3.31959200	0.22819500	0.14100500
C	-4.49428900	2.89269800	-0.28538800
H	-5.35104500	3.58383300	-0.34377000
H	-3.58430000	3.48506200	-0.48411100
C	-5.82616500	0.36038700	0.34777300
H	-6.72792500	-0.23769400	0.55120400
C	-3.20724900	1.33740600	1.20513300
H	-3.18324800	0.87763300	2.20836100
H	-2.26961900	1.90285400	1.07596200
C	-4.65031100	1.78420100	-1.33257700
H	-4.70277800	2.22524500	-2.34020900
C	-4.60816900	-0.57703300	0.39437400
H	-4.73275600	-1.33708000	-0.39280900
H	-4.56340200	-1.09289500	1.36840400
C	-4.41886500	2.27286000	1.11344900
H	-4.30339500	3.06800700	1.86587800
C	-5.92394500	0.98387000	-1.04738800
H	-6.05591000	0.19556100	-1.80811300
H	-6.80399600	1.64520000	-1.10424700
C	-3.43457900	0.85048800	-1.26776800
H	-2.51554200	1.41212000	-1.51178300
H	-3.53352300	0.04033900	-2.01551100
C	-5.68879100	1.46683900	1.39635400
H	-6.57298500	2.12437300	1.36459500
H	-5.64169200	1.03045700	2.40885200
Au	0.08041100	0.28149700	-1.05216500
N	-0.09759000	-1.52554000	-2.36722700
P	-1.73290000	-0.75576400	0.13284300
C	0.28031800	2.04090000	-0.04075100
C	1.28126600	2.22913000	0.91392800
C	-0.52834100	3.13391900	-0.38820000
C	1.43771800	3.45965800	1.56324200
C	-0.37950100	4.36160500	0.25062100
C	0.59995100	4.53520500	1.24066700
H	2.22617100	3.56587600	2.30869300
H	-1.01084000	5.21350400	-0.00817000
H	-1.28391300	3.04832500	-1.17238200
C	1.73837300	1.09299900	-1.99082000
C	2.93494400	0.69501300	-1.52825500
H	1.96974500	1.41777800	1.16296600
O	0.66709500	5.75831800	1.80792100
C	1.65174100	5.98345700	2.79471700
H	1.51417600	5.31312700	3.65993400
H	2.66758500	5.84317500	2.38826500
H	1.53497100	7.02239100	3.12147600

C	4.28224300	1.30352000	-1.78411000
H	5.05795500	0.52570700	-1.77894400
H	4.29065300	1.73058700	-2.79621500
C	1.60622100	2.30904100	-2.88172300
H	1.58267900	3.18152100	-2.20237700
H	2.51312600	2.43041000	-3.49514700
C	-2.01617400	-2.22525300	-0.91219400
O	2.88039800	-0.20922900	-0.40860000
S	3.81039600	-1.53447000	-0.29432900
O	3.03656200	-2.52191100	0.44526100
O	4.44674500	-1.82149700	-1.57066100
C	5.07570200	-0.91985100	0.93098300
F	4.45921100	-0.16574700	1.83148000
F	6.00679400	-0.21502500	0.31541300
F	5.61024500	-1.97157800	1.51246100
C	4.57832200	2.39025100	-0.74447800
H	5.58399700	2.80973500	-0.88671700
H	3.84334300	3.20711100	-0.81687000
H	4.52015800	1.98514600	0.27779700
C	0.37543900	2.37058000	-3.78363600
H	0.45091200	1.68709900	-4.64178400
H	-0.54494400	2.11637900	-3.22784400
H	0.24832000	3.38422800	-4.18835700

110

TS2-E E(MN15(SMD)/cc-pVTZ(pp))=-3162.84473868 imaginary frequency= -342.1875

C	-2.61072200	-3.60997900	-0.68189500
H	-3.59103600	-3.31578800	-0.30645500
C	-2.41866400	-4.91550400	-1.12272200
H	-3.24421700	-5.62795900	-1.09465600
C	-1.16290600	-5.30277900	-1.59677100
H	-0.99498900	-6.32256600	-1.94659100
C	-0.11731600	-4.38491500	-1.61625500
H	0.86327500	-4.70017200	-1.97861000
C	-0.30201900	-3.06748700	-1.17203000
C	1.37714100	-1.99850400	-2.54893900
H	1.67059200	-2.96817800	-2.98650100
H	2.26816800	-1.36255100	-2.48630000
H	0.61945600	-1.52764800	-3.19255200
C	1.89993000	-2.64685300	-0.28507500
H	1.50199300	-2.73533300	0.73620000
H	2.73516000	-1.93391300	-0.30820400
H	2.27790500	-3.63299400	-0.60723700
C	-2.19765100	-1.12163200	1.74661800
C	-1.25984300	-2.20247100	2.32432500
H	-0.21001200	-1.93536800	2.08640400
H	-1.46709600	-3.17972100	1.85801200
C	-1.44640800	-2.30988800	3.84277700

H	-0.77235300	-3.09251000	4.22443000
C	-1.84252600	0.21199400	2.43456800
H	-0.78358000	0.44090100	2.22680500
H	-2.43518800	1.04474200	2.02306000
C	-3.83471700	-1.60734500	3.59052900
H	-4.88124800	-1.87856200	3.79851100
C	-3.50882200	-0.26222700	4.24559700
H	-3.66806800	-0.32454300	5.33459400
H	-4.18459300	0.52048600	3.86008200
C	-2.05240800	0.10680500	3.94911800
H	-1.81389100	1.08155200	4.40366500
C	-3.65523100	-1.48967900	2.06916600
H	-4.33663700	-0.71429800	1.68082800
H	-3.92712100	-2.44641900	1.59284300
C	-1.11819700	-0.97005500	4.50602100
H	-1.23558200	-1.05077500	5.59912400
H	-0.06559200	-0.69586600	4.30798300
C	-2.90086900	-2.69002800	4.13950800
H	-3.13812200	-3.66512100	3.68065000
H	-3.04728800	-2.79655600	5.22686100
C	-3.07453900	-0.13679700	-1.14186600
C	-3.62734400	2.22691700	-2.81860900
H	-4.27692200	2.81821000	-3.48469500
H	-2.68800400	2.79242100	-2.68924100
C	-5.32037100	-0.10514400	-2.27732800
H	-6.25621700	-0.67140800	-2.40288500
C	-3.39921400	1.23555900	-0.52088700
H	-3.91979900	1.09213400	0.44167400
H	-2.46678200	1.79218900	-0.31582200
C	-3.33883700	0.86036800	-3.44794600
H	-2.83793000	0.99360700	-4.41955200
C	-4.38088800	-0.91755300	-1.37119800
H	-4.15802200	-1.87458700	-1.87010700
H	-4.87934000	-1.14018500	-0.41354200
C	-4.31136500	2.03184000	-1.46215900
H	-4.51579700	3.01253400	-1.00479800
C	-4.64954700	0.09296200	-3.63902600
H	-4.45299700	-0.88458000	-4.11131400
H	-5.31776800	0.65434400	-4.31252600
C	-2.40532300	0.07371200	-2.51912000
H	-1.45928800	0.63488100	-2.39326600
H	-2.15504600	-0.90897200	-2.96161000
C	-5.62019600	1.25844100	-1.64769900
H	-6.30829400	1.82588200	-2.29561900
H	-6.12243600	1.12676600	-0.67371400
Au	0.32823100	0.09319600	-0.42140600
N	0.83758700	-2.15746200	-1.18500900

P	-1.78654600	-0.97163700	-0.07751100
C	0.42617300	2.14739100	-0.00175600
C	0.38671600	2.63412300	1.31140100
C	-0.12499600	2.94841500	-1.02361500
C	-0.20510000	3.86217000	1.61017000
C	-0.72123000	4.16801300	-0.73234600
C	-0.77435300	4.63930300	0.58952800
H	-0.20388000	4.20790600	2.64385400
H	-1.15391300	4.78632400	-1.52086900
H	-0.09269900	2.61399100	-2.06319300
C	2.10879400	1.23475100	-0.54226700
C	2.96601400	0.80088200	0.41818200
H	0.83125900	2.06578700	2.12919400
O	-1.36832800	5.83289700	0.77637900
C	-1.41348100	6.36469100	2.08556200
H	-1.97754100	5.70537800	2.76642300
H	-0.39926200	6.52076200	2.48889500
H	-1.92517400	7.33011700	2.01184600
C	2.68745900	0.18330800	1.75962400
H	1.59482500	0.10646400	1.90181100
H	3.06483400	-0.85240700	1.76271400
C	2.58051500	1.96232900	-1.78590400
H	2.21659200	2.99857600	-1.71446900
H	3.67629900	2.05163500	-1.76681900
C	-1.56578200	-2.66512100	-0.70561500
O	4.34689900	1.05792400	0.26108900
S	5.24082400	0.18121700	-0.78475000
O	4.37304800	-0.70180500	-1.55818300
O	6.20562900	1.07760100	-1.38240100
C	6.10918100	-0.87211000	0.47167300
F	5.21309500	-1.69432500	1.01074200
F	6.62646100	-0.10201300	1.40786400
F	7.04958200	-1.56631000	-0.13115100
C	3.35039100	0.95511900	2.90412000
H	4.44334500	0.94132900	2.79249500
H	3.03109500	2.00820000	2.91881100
H	3.09879300	0.49933800	3.87175300
C	2.14455100	1.35947700	-3.12016100
H	2.73378200	0.46446400	-3.35891600
H	1.07647500	1.07486200	-3.11797700
H	2.29531100	2.08390800	-3.93268200

118

TS2-E OTf- E(MN15(SMD)/cc-pVTZ(pp))=-4124.11641344 imaginary frequency= -357.0528

C	-0.05816900	-4.04567900	0.30393800
H	-1.00787900	-4.50928700	0.57484800
C	1.09089100	-4.82675800	0.24186400
H	1.03558400	-5.89447800	0.46139600

C	2.31032800	-4.23276100	-0.09530000
H	3.22857800	-4.81887700	-0.13936500
C	2.37778500	-2.86870700	-0.36309200
H	3.33873800	-2.41215200	-0.61305400
C	1.22437200	-2.07512800	-0.29346700
C	1.98097500	-0.38469100	-1.85267600
H	2.99423200	-0.81393700	-1.89773400
H	2.07085700	0.70201400	-1.96614800
H	1.33137000	-0.79757200	-2.63978900
C	2.23589100	-0.05675400	0.52429700
H	1.73779300	-0.16005500	1.50078400
H	2.39069800	1.00485700	0.28656600
H	3.21034000	-0.57006200	0.56271700
C	-2.03515400	-1.69077300	1.97567600
C	-0.75940500	-1.65549800	2.84329100
H	-0.15881100	-0.76585900	2.56940400
H	-0.13557900	-2.54178100	2.64495900
C	-1.12874300	-1.60647600	4.33019100
H	-0.19989800	-1.59039700	4.92111100
C	-2.84397400	-0.41832000	2.29474300
H	-2.21921700	0.45864700	2.05691600
H	-3.74447700	-0.34733200	1.66297300
C	-3.22511400	-2.88166000	3.84263800
H	-3.81385800	-3.77910500	4.09051800
C	-4.05480700	-1.62564000	4.12514600
H	-4.34839800	-1.59889200	5.18804300
H	-4.98311300	-1.64340600	3.52774800
C	-3.23026200	-0.38288400	3.77708600
H	-3.82642800	0.52531800	3.96345400
C	-2.86431700	-2.93001200	2.35016200
H	-3.79144400	-2.96217800	1.75327200
H	-2.29518100	-3.85024800	2.13794500
C	-1.95481500	-0.35186500	4.62327100
H	-2.20844100	-0.30666300	5.69583300
H	-1.36659900	0.55309100	4.38503300
C	-1.94510500	-2.85405700	4.68341500
H	-1.34719900	-3.76170600	4.49407000
H	-2.20033800	-2.84659000	5.75650400
C	-2.72088200	-2.15197600	-1.07818400
C	-4.40665500	-1.26935200	-3.33548500
H	-5.12553400	-1.46435500	-4.14914900
H	-4.19057900	-0.18678000	-3.33779800
C	-4.03315100	-3.96135500	-2.23610700
H	-4.24790000	-5.04176600	-2.23065300
C	-4.03274200	-1.37708000	-0.85428200
H	-4.48887600	-1.68569200	0.10225800
H	-3.82606000	-0.29324100	-0.78966900

C	-3.11809200	-2.06373500	-3.57285500
H	-2.66733900	-1.76747100	-4.53316400
C	-3.01596200	-3.66145700	-1.12417100
H	-2.08575900	-4.21181400	-1.33745200
H	-3.40344600	-4.01504100	-0.15444600
C	-5.01783300	-1.67540900	-1.99111200
H	-5.94225000	-1.10220600	-1.81571500
C	-3.43187200	-3.56180500	-3.58604500
H	-2.51238200	-4.14056100	-3.77692300
H	-4.14090800	-3.79056000	-4.39960200
C	-2.12595300	-1.74978900	-2.44701300
H	-1.90168900	-0.66657800	-2.45106500
H	-1.17185000	-2.28573600	-2.60537800
C	-5.32638700	-3.17598600	-1.99641800
H	-6.05899100	-3.41009100	-2.78719900
H	-5.77741900	-3.47147700	-1.03295300
Au	-0.62577300	0.56296200	-0.40465200
N	1.35844200	-0.63753200	-0.52738500
P	-1.46692200	-1.59921500	0.19029300
C	-2.06037500	2.09528600	-0.52999400
C	-2.71969800	2.59392200	0.60692500
C	-2.80260900	2.05116000	-1.73359400
C	-4.05364100	2.99776100	0.56619600
C	-4.13289700	2.44787500	-1.78315800
C	-4.77932600	2.91981600	-0.63194200
H	-4.51225000	3.38383000	1.47681100
H	-4.69931200	2.40310800	-2.71530700
H	-2.32962300	1.69341600	-2.65118800
C	-0.23286600	2.63083400	-0.77219900
C	0.46736400	3.12293500	0.28640700
H	-2.18740100	2.68664400	1.55423800
O	-6.07386800	3.28601400	-0.77182800
C	-6.74694000	3.78846800	0.36084300
H	-6.78547500	3.04119700	1.17195100
H	-6.26624400	4.70548800	0.74190400
H	-7.76778400	4.02416100	0.04000200
C	0.44327800	2.71532600	1.73267500
H	-0.26860300	1.88081500	1.86256200
H	1.42957000	2.30622800	2.00380800
C	-0.14905100	3.25792500	-2.15232800
H	-1.13650800	3.69182800	-2.37303200
H	0.53397000	4.11869300	-2.12418100
C	-0.01070200	-2.66308300	0.03380600
O	1.25826300	4.27918600	0.10713200
S	2.70485500	4.19188000	-0.65174400
O	2.85220900	2.89326800	-1.29191400
O	2.88150400	5.45512200	-1.33844500

C	3.78899200	4.20477000	0.85517200
F	3.65977000	3.04551100	1.48304400
F	3.40417200	5.18837600	1.65351600
F	5.03539000	4.38489900	0.47854000
C	0.11798200	3.88664700	2.66369600
H	0.89239300	4.66198100	2.58428700
H	-0.84646600	4.35218600	2.40844800
H	0.07751400	3.54766100	3.70850500
C	0.26364300	2.32256500	-3.28816000
H	1.34405600	2.13272000	-3.26606500
H	-0.24985600	1.34569600	-3.22954000
H	0.01938400	2.77346700	-4.26073800
O	4.97721900	-1.78581200	1.15651900
S	5.81536400	-1.49131800	-0.03568900
O	5.00302100	-1.13323200	-1.23567000
O	7.03180600	-0.69949800	0.18247500
C	6.43360700	-3.17282700	-0.49549200
F	5.39476400	-3.98440600	-0.78501700
F	7.11500800	-3.73307000	0.50358900
F	7.22008300	-3.13065900	-1.56968500

110

TS2-Z E(MN15(SMD)/cc-pVTZ(pp))=-3162.8390623 imaginary frequency= -344.4442

C	-2.74094600	-3.31844700	-1.03674200
H	-3.45057600	-3.22184100	-0.21400300
C	-2.82457700	-4.42323200	-1.87904800
H	-3.60364600	-5.17058800	-1.72262200
C	-1.89974200	-4.56973000	-2.91569700
H	-1.94936600	-5.43205200	-3.58236500
C	-0.90201900	-3.61555300	-3.09229300
H	-0.17377000	-3.74556200	-3.89577500
C	-0.81428300	-2.49796400	-2.25055500
C	0.21335000	-0.97287200	-3.82123900
H	0.30259000	-1.74946500	-4.60067500
H	1.05480400	-0.27458500	-3.92783500
H	-0.73261900	-0.42864900	-3.95621200
C	1.58265700	-2.17028600	-2.24455700
H	1.62268500	-2.59887100	-1.23472800
H	2.36142200	-1.39869600	-2.33983400
H	1.78106000	-2.96713500	-2.98340900
C	-1.13776200	-1.67635200	1.61062700
C	-0.22522100	-2.89538900	1.36645100
H	0.64763800	-2.57581400	0.76399600
H	-0.76044000	-3.67241200	0.79686700
C	0.24629200	-3.47925100	2.70560000
H	0.88521700	-4.35216100	2.49798300
C	-0.30402600	-0.63516500	2.38492000
H	0.58868200	-0.38644500	1.78361700

H	-0.87166500	0.30007600	2.53033600
C	-1.86560800	-2.69627000	3.78870200
H	-2.74666900	-3.00100200	4.37461900
C	-1.08038000	-1.62510300	4.55323300
H	-0.76234400	-2.01958400	5.53226600
H	-1.72624100	-0.75092700	4.74661300
C	0.14469900	-1.20962700	3.73370200
H	0.71584900	-0.43476300	4.26896900
C	-2.34760900	-2.11450400	2.45203800
H	-3.01036500	-1.25600900	2.64782200
H	-2.93580200	-2.87477400	1.90899500
C	1.03744700	-2.42823900	3.48609300
H	1.37508000	-2.85076100	4.44709700
H	1.93346800	-2.12538400	2.92306800
C	-0.97427100	-3.91264800	3.52312700
H	-1.54349700	-4.68692600	2.98017900
H	-0.64687400	-4.35636800	4.47763900
C	-3.13739000	0.08189000	-0.12019300
C	-4.22365500	2.79164700	-0.57166600
H	-5.06395200	3.50033600	-0.65632800
H	-3.29277900	3.36468800	-0.72508800
C	-5.64518500	0.28571800	-0.03711000
H	-6.57415500	-0.28457300	0.11818700
C	-3.03137300	1.18882700	0.94917700
H	-3.03926100	0.74169000	1.95755500
H	-2.07625400	1.73388300	0.83677000
C	-4.35349700	1.69800600	-1.63668800
H	-4.34283800	2.15012100	-2.64096400
C	-4.45918100	-0.68741700	0.05179300
H	-4.55947800	-1.43208000	-0.75469200
H	-4.48352500	-1.22167000	1.01472000
C	-4.21840800	2.15175500	0.81985600
H	-4.11807700	2.93473900	1.58769500
C	-5.65964500	0.92863000	-1.42609800
H	-5.77476200	0.15252700	-2.20188200
H	-6.51774000	1.61466200	-1.51801100
C	-3.15862700	0.74319400	-1.51717900
H	-2.22299700	1.31417100	-1.67037300
H	-3.20626900	-0.03955500	-2.29777100
C	-5.51852800	1.37138400	1.03572000
H	-6.38331300	2.05305600	0.98248400
H	-5.52331700	0.91531300	2.04088400
Au	0.19764000	0.37687800	-0.97100300
N	0.26756100	-1.54341600	-2.46609500
P	-1.56997800	-0.93486200	-0.05961700
C	0.58510100	2.25828200	-0.11824900
C	1.12756000	2.30563300	1.17348300

C	-0.27217900	3.30165400	-0.52269400
C	0.82504300	3.35573300	2.04139400
C	-0.58014400	4.34470300	0.34119400
C	-0.03636600	4.38611000	1.63502700
H	1.27257500	3.35584600	3.03530100
H	-1.24703200	5.15191500	0.03314200
H	-0.71693400	3.30098000	-1.51929700
C	1.76545300	1.64419200	-1.59720600
C	2.96855000	1.09884300	-1.28581500
H	1.78563900	1.51614000	1.53271300
O	-0.38822300	5.43741600	2.39991100
C	0.15757400	5.53363400	3.70055600
H	-0.13906400	4.67389200	4.32434000
H	1.25799000	5.59539400	3.66902300
H	-0.24359000	6.45347100	4.13918800
C	4.29355000	1.35052500	-1.95601700
H	4.98233600	0.53526700	-1.69806300
H	4.13418400	1.28370500	-3.04482500
C	1.64498400	2.65985200	-2.71621600
H	1.56652500	3.65308000	-2.24577400
H	2.58764800	2.67968500	-3.28294400
C	-1.74853300	-2.33436100	-1.21189200
O	2.91450700	0.14146200	-0.23034400
S	4.01850500	-0.08919400	0.94536100
O	5.19666500	0.73844600	0.76289400
O	3.28805500	-0.16160400	2.20021200
C	4.42804100	-1.83021900	0.44284100
F	4.71118200	-1.84838200	-0.85236000
F	3.37601800	-2.60217200	0.67782100
F	5.46147900	-2.24190800	1.14173300
C	0.48413800	2.46804600	-3.69807900
H	0.74027100	1.76494200	-4.50191100
H	-0.42307100	2.08002200	-3.20269300
H	0.22216300	3.42249500	-4.17594300
C	4.91465800	2.70794900	-1.60874000
H	4.24182000	3.53271300	-1.88650600
H	5.12389500	2.77726500	-0.53404800
H	5.86005000	2.84203400	-2.15260800

118

TS2-Z OTf- E(MN15(SMD)/cc-pVTZ(pp))=-4124.11670422 imaginary frequency= -345.6907

C	-1.77786200	-2.25040700	3.48673300
H	-2.86678600	-2.26312300	3.41716600
C	-1.16249000	-2.75477700	4.62750400
H	-1.76679600	-3.17495600	5.43311600
C	0.23129300	-2.70942900	4.73521000
H	0.72757800	-3.09843200	5.62609500
C	0.99191200	-2.16615000	3.70541400

H	2.08059500	-2.13517400	3.78697900
C	0.37836500	-1.65897800	2.54963700
C	2.06227300	-2.21932600	0.94476100
H	2.71684200	-2.64722800	1.71950500
H	2.71094200	-1.78090100	0.17955100
H	1.40756100	-2.99428100	0.51608100
C	2.07167800	-0.01409500	1.96528800
H	1.42555100	0.79588600	2.33516000
H	2.68454800	0.32415100	1.11662700
H	2.77499800	-0.34520100	2.74446100
C	-2.73791500	0.57639000	1.68958000
C	-1.92363300	1.15401800	2.86648500
H	-0.89723100	1.38305200	2.52027700
H	-1.84096400	0.41424000	3.67880300
C	-2.59952100	2.42224000	3.40557300
H	-2.00009500	2.80295100	4.24797600
C	-2.78639100	1.66600800	0.59991900
H	-1.75056800	1.92054700	0.31623200
H	-3.29137600	1.29681000	-0.30926200
C	-4.82790400	1.52845900	2.71442200
H	-5.84472200	1.27414700	3.05439600
C	-4.90284100	2.57155200	1.59402600
H	-5.41650300	3.47761200	1.95740200
H	-5.49466100	2.17383400	0.75071400
C	-3.48626700	2.92284100	1.13028100
H	-3.52289300	3.66862500	0.32033200
C	-4.16032900	0.25564900	2.17596600
H	-4.76711700	-0.14781000	1.34878500
H	-4.12784200	-0.50942900	2.97111300
C	-2.68657600	3.48383500	2.30847800
H	-3.17962300	4.38935700	2.70210300
H	-1.68101700	3.77154300	1.97033000
C	-4.01065400	2.07413000	3.88893400
H	-3.96441100	1.32279800	4.69656500
H	-4.49791300	2.97300600	4.30284400
C	-2.81452400	-2.14319500	0.07087400
C	-3.27935600	-3.39307400	-2.56635800
H	-3.79905800	-4.11068100	-3.22371400
H	-2.55857500	-2.83508200	-3.18782600
C	-4.55775600	-3.95865700	0.00937500
H	-5.28018800	-4.51480900	0.62794900
C	-3.56365500	-1.40814300	-1.05879000
H	-4.30339000	-0.71011000	-0.63128600
H	-2.85072200	-0.80997100	-1.65569800
C	-2.54441300	-4.14380400	-1.45126200
H	-1.80289900	-4.83068300	-1.88880000
C	-3.80436300	-2.96862100	0.91074500

H	-3.24951900	-3.53910900	1.67372500
H	-4.52342900	-2.31624100	1.43089700
C	-4.29111900	-2.42350900	-1.94839100
H	-4.81626100	-1.87702000	-2.74782900
C	-3.55384100	-4.93199900	-0.61338500
H	-3.03304700	-5.49613600	0.17912900
H	-4.07969700	-5.66449900	-1.24896000
C	-1.80853700	-3.12904200	-0.56672400
H	-1.08001600	-2.57065400	-1.18361700
H	-1.24230600	-3.64592700	0.23051100
C	-5.29936700	-3.20169600	-1.09688200
H	-5.85842200	-3.91350100	-1.72751700
H	-6.03498500	-2.50753400	-0.65374100
Au	0.06860000	-0.26330300	-0.37316100
N	1.22002100	-1.12964200	1.49044100
P	-1.78598000	-0.88837200	0.99566200
C	-0.36597900	0.47947700	-2.28755700
C	-1.04927700	1.70213700	-2.40209200
C	-0.60644000	-0.49669400	-3.27885900
C	-1.93801800	1.94314600	-3.45077900
C	-1.49572800	-0.26277800	-4.32188400
C	-2.17511100	0.95865600	-4.42042600
H	-2.43953400	2.91025700	-3.49544600
H	-1.67870400	-1.02203500	-5.08468500
H	-0.09373900	-1.45880600	-3.23569800
C	1.51974000	0.47941400	-1.72478100
C	2.03573900	1.64942100	-1.27388800
H	-0.90754500	2.48856100	-1.66235500
O	-3.02097500	1.09941000	-5.46998700
C	-3.69285100	2.32957200	-5.61447800
H	-4.34626800	2.53848800	-4.74978800
H	-2.98336200	3.16616400	-5.73397100
H	-4.30790100	2.24753600	-6.51789600
C	3.38192300	2.23922400	-1.57522400
H	3.61885600	2.99544000	-0.81573300
H	4.11143600	1.42628400	-1.42850300
C	2.30826300	-0.38026200	-2.69060600
H	1.93154100	-0.15596000	-3.70299200
H	3.35489500	-0.04766700	-2.65392800
C	-1.02183900	-1.70170000	2.43169200
O	1.17176300	2.33688400	-0.35832500
S	1.01203100	3.93245600	-0.17112100
O	1.86657200	4.70757400	-1.05325200
O	-0.41554700	4.19841900	-0.04901000
C	1.72211900	3.98738300	1.54444400
F	2.88402300	3.36085900	1.56112900
F	0.87848100	3.38862000	2.37682800

F	1.87716000	5.24957300	1.89608700
C	2.29623900	-1.89452500	-2.45004000
H	3.13792900	-2.18647100	-1.81003000
H	1.36782500	-2.23970400	-1.96001700
H	2.39200800	-2.43739500	-3.40196100
C	3.48854500	2.83774400	-2.98073900
H	3.27832200	2.07730400	-3.74788100
H	2.78604900	3.67138300	-3.11471200
H	4.50714500	3.21410500	-3.15176800
O	4.89311000	-1.30014200	1.84226800
S	5.41216400	-0.97175600	0.49345900
O	4.35591900	-0.45729000	-0.44147300
O	6.70199500	-0.27750600	0.41645700
C	5.72617500	-2.64040000	-0.24287000
F	4.63217100	-3.41735300	-0.11786200
F	6.73889100	-3.26233500	0.35293800
F	5.99456600	-2.54089400	-1.54737700

71

INT3 E(MN15(SMD)/cc-pVTZ(pp))=-1621.46087192

C	-0.04571400	0.59480600	2.83983200
H	-0.24395300	-0.46793500	2.98464000
C	0.03651300	1.41864700	3.95785300
H	-0.09587500	0.99752100	4.95519800
C	0.29034900	2.78055800	3.78655000
H	0.36395800	3.44314500	4.65028000
C	0.44520100	3.29406700	2.50260600
H	0.63872100	4.36123800	2.37172800
C	0.35697200	2.48016100	1.36347900
C	1.85853500	3.64366900	-0.12861600
H	2.09506000	4.45814000	0.58191500
H	1.94316300	4.04384900	-1.14969100
H	2.59704100	2.83908700	-0.00219200
C	-0.52370900	4.12381500	-0.17145500
H	-1.51914900	3.67304100	-0.04817600
H	-0.42809800	4.49813700	-1.20138700
H	-0.43750900	4.98681500	0.51493500
C	-1.72659300	-0.87421500	0.18170900
C	-2.72663700	0.22550000	0.59348600
H	-2.62761300	1.08852800	-0.09456600
H	-2.50164100	0.58573600	1.61053500
C	-4.15602700	-0.33140800	0.55407200
H	-4.84849500	0.46944000	0.85558800
C	-2.08734500	-1.34736000	-1.24446900
H	-2.01524600	-0.48944400	-1.93781000
H	-1.37197400	-2.10879700	-1.59456800
C	-3.28630700	-2.60910200	1.11748700
H	-3.35153100	-3.45083700	1.82398900

C	-3.61940600	-3.08985400	-0.29652100
H	-4.63868100	-3.50804900	-0.32172700
H	-2.92793400	-3.89556800	-0.59720300
C	-3.51327700	-1.91111400	-1.26762800
H	-3.73370600	-2.24924100	-2.29168000
C	-1.84740600	-2.07001000	1.14746200
H	-1.15132700	-2.87151800	0.84855800
H	-1.59503900	-1.76944600	2.17745200
C	-4.49493800	-0.80991500	-0.85963400
H	-5.52768300	-1.19357900	-0.88859800
H	-4.43844500	0.03165000	-1.57105400
C	-4.26042800	-1.50374600	1.53530000
H	-4.03336300	-1.16310900	2.56015100
H	-5.29017700	-1.89616000	1.54312200
C	1.45571800	-1.22110300	0.20522900
C	3.80631100	-2.26412500	-1.23404500
H	4.71444100	-2.88582600	-1.29080500
H	3.69161100	-1.76310700	-2.21050100
C	2.91389700	-2.80294500	1.50651400
H	3.02660100	-3.30185900	2.48133100
C	1.32087400	-2.28152400	-0.90274400
H	0.44848800	-2.92749000	-0.70580600
H	1.15583300	-1.78126600	-1.87516000
C	3.95575300	-1.22356300	-0.11994300
H	4.82264600	-0.57680000	-0.32505400
C	1.66410200	-1.90988500	1.56961100
H	1.81533200	-1.14767800	2.35081700
H	0.78487900	-2.51413500	1.84533200
C	2.58747300	-3.14568800	-0.94781300
H	2.47062600	-3.89246000	-1.74787600
C	4.13928400	-1.93009000	1.22569600
H	4.27070600	-1.18850400	2.03192200
H	5.04875600	-2.55205200	1.20117000
C	2.69657100	-0.34755200	-0.07905400
H	2.57337700	0.16644700	-1.05174400
H	2.79082800	0.42730600	0.70414300
C	2.75784100	-3.85043500	0.40128600
H	3.64522900	-4.50343600	0.37685000
H	1.88502200	-4.49434600	0.60570800
Au	0.12841200	1.23172700	-1.68987300
N	0.50728000	3.10617100	0.06666800
P	-0.02583900	-0.08874000	0.15037600
C	0.11693200	1.09642100	1.53173600

39

prod-E E(MN15(SMD)/cc-pVTZ(pp))=-1541.40219683

C	-1.95656700	0.37512300	0.48807600
C	-2.34316900	-0.23003800	-0.71266600

C	-2.96105800	0.70593300	1.41501200
C	-3.68197100	-0.52489800	-0.98708000
C	-4.29362500	0.41353000	1.15970100
C	-4.66596200	-0.20556700	-0.04428500
H	-3.94225600	-0.99191100	-1.93695500
H	-5.07950600	0.65341000	1.87764500
H	-2.68833600	1.18485400	2.35886400
C	-0.53032000	0.71690000	0.73734900
C	0.41820400	-0.23295000	0.69489000
H	-1.57444800	-0.46204600	-1.45491800
O	-5.99079100	-0.44767000	-0.20612600
C	-6.40666900	-1.06292700	-1.40260800
H	-6.15658500	-0.44679100	-2.28352800
H	-5.94944900	-2.05996200	-1.52580100
H	-7.49540600	-1.17239800	-1.33827600
C	0.30839800	-1.71648200	0.53090800
H	-0.75414900	-1.98974900	0.48512000
H	0.75702900	-1.98850800	-0.44131800
C	-0.20843900	2.18008400	0.93566200
H	-0.90866100	2.58940300	1.68369800
H	0.80701400	2.29585800	1.33992400
O	1.76924200	0.18234900	0.87697000
S	2.58937000	0.63383100	-0.44704500
O	1.79871400	0.36789600	-1.64153100
O	3.22789200	1.90978300	-0.18082600
C	3.89271700	-0.67779700	-0.34117200
F	3.34250300	-1.85355300	-0.61346100
F	4.40570800	-0.70013600	0.87705900
F	4.83857900	-0.41226200	-1.22519100
C	1.01420800	-2.47559800	1.65684400
H	0.55137300	-2.25280100	2.62962400
H	0.95771500	-3.56008800	1.48806200
H	2.07559200	-2.19539000	1.71769800
C	-0.35058300	2.96293400	-0.37306800
H	-1.36435500	2.84978400	-0.78784000
H	-0.16121900	4.03388100	-0.21208000
H	0.36317000	2.59635700	-1.12614900

39

prod-Z E(MN15(SMD)/cc-pVTZ(pp))=-1541.40000017

C	1.58949000	0.85938000	0.06598400
C	2.63721800	0.98273700	0.98348800
C	1.68614800	-0.14038100	-0.91783400
C	3.74135300	0.12715500	0.95245400
C	2.78039200	-0.99336100	-0.96586100
C	3.81584000	-0.86990900	-0.02801500
H	4.53071800	0.24629000	1.69466300
H	2.86163500	-1.77214900	-1.72533200

H	0.89585000	-0.23259700	-1.66702500
C	0.43108200	1.79449300	0.10314300
C	-0.82949300	1.35907800	0.26017000
H	2.58689000	1.75250100	1.75822600
O	4.84294600	-1.74815400	-0.15047700
C	5.89735200	-1.66294100	0.77781500
H	6.40481000	-0.68378600	0.72752800
H	5.54295100	-1.82576900	1.81043500
H	6.61245600	-2.45124200	0.51536300
C	-2.11168800	2.13085800	0.24594700
H	-1.91462800	3.15773400	-0.09061300
H	-2.76505500	1.67898100	-0.51856100
C	0.75006500	3.25651000	-0.11302700
H	1.67629200	3.48713200	0.43847000
H	-0.03459600	3.90194500	0.30823700
O	-1.01304600	-0.01149500	0.60103600
S	-1.54297800	-1.05367200	-0.51831300
O	-1.71632800	-0.36766600	-1.79282100
O	-0.81022600	-2.29557800	-0.37003300
C	-3.23058700	-1.37115500	0.18793500
F	-4.02980300	-0.34364100	-0.06697200
F	-3.14176000	-1.54694100	1.49644400
F	-3.71712900	-2.46108000	-0.37956900
C	-2.81038200	2.12251900	1.60704100
H	-3.79477000	2.60799700	1.54900200
H	-2.95902300	1.09190700	1.96130800
H	-2.20625900	2.64959300	2.36063700
C	0.95417500	3.55789000	-1.59899000
H	1.26693000	4.60001500	-1.75885900
H	1.72650500	2.89751600	-2.02312700
H	0.02262100	3.38355200	-2.15961900

10

2-butyne E(MN15(SMD)/cc-pVTZ(pp))=-155.830603147

C	0.60714000	-0.00014700	-0.00000700
C	-0.60713000	-0.00011500	0.00003500
C	-2.06985300	0.00006600	-0.00000600
H	-2.46794700	0.52612800	-0.88046700
H	-2.46814200	-1.02539100	-0.01534900
H	-2.46802900	0.49960800	0.89572500
C	2.06984900	0.00006900	-0.00000300
H	2.46795800	0.50626800	0.89201200
H	2.46820300	-1.02545300	-0.00770200
H	2.46792100	0.51960600	-0.88433800

104

INT1-Me E(MN15(SMD)/cc-pVTZ(pp))=-3084.29919723

C	1.31416500	3.00029300	0.65489400
H	0.54168800	3.71927500	0.92362200

C	2.64713300	3.39760600	0.67039000
H	2.90282800	4.41466800	0.97059000
C	3.64602600	2.49470800	0.29754600
H	4.69834100	2.77977200	0.30977500
C	3.31259000	1.20078800	-0.09259900
H	4.09733600	0.49786000	-0.38200400
C	1.97433600	0.79848200	-0.09432300
C	2.30819500	-1.49779500	0.54000200
H	3.35023500	-1.19446900	0.74765900
H	2.31411800	-2.51898600	0.13618000
H	1.72665000	-1.44731400	1.47474700
C	2.25548400	-0.87301800	-1.81964800
H	1.83940400	-0.15784300	-2.54429000
H	2.00067500	-1.90391200	-2.09739000
H	3.35510400	-0.81443900	-1.76420300
C	-1.61622700	2.08839800	-1.14428400
C	-1.13616800	1.43055500	-2.45772200
H	-1.41592300	0.36004800	-2.47454500
H	-0.03250800	1.48956000	-2.51411800
C	-1.77621000	2.13300000	-3.66201000
H	-1.42314300	1.64038300	-4.58105600
C	-3.14934800	2.03515600	-1.03491800
H	-3.50583100	1.00134200	-0.90834300
H	-3.46189900	2.60569900	-0.14548400
C	-1.85206600	4.27166000	-2.38029400
H	-1.54290900	5.32779800	-2.36650400
C	-3.37577000	4.17230300	-2.29059900
H	-3.83536000	4.68174800	-3.15289100
H	-3.74137400	4.67689900	-1.38034000
C	-3.77950900	2.69587100	-2.27012700
H	-4.87345900	2.60772900	-2.18728600
C	-1.19193600	3.57273600	-1.17942600
H	-1.48628500	4.07606800	-0.24139900
H	-0.10030100	3.65455700	-1.28711400
C	-3.29937600	2.01071300	-3.55296700
H	-3.77679500	2.47974900	-4.42823100
H	-3.59880100	0.94717100	-3.55586300
C	-1.36861500	3.60816000	-3.67220400
H	-0.27331400	3.69956200	-3.76332000
H	-1.81285600	4.11359700	-4.54463800
C	-1.45252700	1.20661200	1.99034300
C	-2.56601800	-0.26273000	4.28540100
H	-2.90414500	-0.33053000	5.33185600
H	-2.71518900	-1.25783500	3.82982000
C	-1.71820600	2.54011000	4.10813700
H	-1.56336500	3.53841500	4.54495400
C	-2.95302000	0.86308100	2.06609100

H	-3.54123700	1.65230700	1.57772000
H	-3.17091900	-0.08192800	1.54929600
C	-1.08580400	0.12645800	4.23857700
H	-0.47997000	-0.62092700	4.77310400
C	-1.23681700	2.58610600	2.64712300
H	-0.16729500	2.84011300	2.65324200
H	-1.78246100	3.36650300	2.08586200
C	-3.39132300	0.78606200	3.53627000
H	-4.45592700	0.50747600	3.55845800
C	-0.88645200	1.50608300	4.87241400
H	0.18107600	1.78239300	4.85323500
H	-1.19833000	1.48045300	5.92882400
C	-0.62417900	0.16487100	2.77594300
H	-0.75542000	-0.83834300	2.32578400
H	0.44947600	0.42164100	2.71976800
C	-3.19820100	2.16158300	4.17843100
H	-3.52934000	2.14325500	5.22928900
H	-3.81306800	2.91340500	3.65502000
Au	-0.44407400	-1.07230200	-0.42615600
N	1.67085900	-0.59167800	-0.47172400
P	-0.77048000	1.14522600	0.24399200
C	-2.38811200	-1.67100100	-0.25999100
C	-3.33239500	-1.51454100	-1.27268700
C	-2.73169500	-2.43395200	0.87107200
C	-4.60895600	-2.08614400	-1.16738800
C	-3.99338400	-3.00285500	0.98622400
C	-4.94911300	-2.83248000	-0.03262500
H	-5.32299300	-1.94001100	-1.97784000
H	-4.27258200	-3.59260500	1.86095400
H	-2.01676500	-2.58621600	1.68543300
C	-0.00705700	-3.44268800	-0.65245500
C	-0.20839100	-3.01509100	-1.79324400
H	-3.10476900	-0.93491100	-2.17101700
O	-6.14192800	-3.41763600	0.17133000
C	-7.13485500	-3.29344700	-0.82932700
H	-7.40879200	-2.23763400	-0.99057700
H	-6.79702500	-3.73270300	-1.78249100
H	-8.01027000	-3.84297800	-0.46817800
C	-0.48597900	-2.70837400	-3.19876700
H	-1.54551000	-2.43982500	-3.32427200
H	0.14513100	-1.88672800	-3.56719000
H	-0.27883900	-3.60060300	-3.80730600
C	0.18786800	-4.17752800	0.60278500
H	0.36277400	-3.49924000	1.45028800
H	-0.70151400	-4.78763700	0.81480600
H	1.06364300	-4.83673300	0.51383300
C	0.95924900	1.68696900	0.28001600

O	5.13551000	-1.27205300	-1.06372600
S	5.98186400	-1.28811900	0.17387400
O	5.22585600	-0.77302800	1.35243000
O	6.80860000	-2.47766300	0.37268300
C	7.15869000	0.09581500	-0.16117300
F	7.90487000	-0.15122200	-1.22973900
F	7.94894900	0.31788900	0.88106200
F	6.45737400	1.22643200	-0.39896000

104

TS1-E-Me E(MN15(SMD)/cc-pVTZ(pp))=-3084.29949982 imaginary frequency= -110.2714

C	-2.25055200	-3.55611500	0.96812000
H	-3.31055700	-3.31556700	0.90272500
C	-1.86983300	-4.84694200	1.31571500
H	-2.62939100	-5.59952200	1.53025100
C	-0.51178400	-5.16808600	1.38068300
H	-0.19474700	-6.17666700	1.64958300
C	0.44610800	-4.19871700	1.10335500
H	1.50253400	-4.46284900	1.16604200
C	0.06786400	-2.89422400	0.75614200
C	2.04703200	-2.37321200	-0.56152800
H	2.46671900	-3.35402600	-0.29633600
H	2.89315200	-1.67690200	-0.66696300
H	1.48789600	-2.46259700	-1.50476700
C	1.89552000	-1.69901000	1.77209400
H	1.25276600	-1.20154600	2.51437300
H	2.80955400	-1.12524600	1.55635500
H	2.21342800	-2.67283000	2.17237200
C	-2.70863000	-0.10894800	1.63375800
C	-1.64284400	0.04172600	2.74303300
H	-0.80582300	0.66867200	2.37862900
H	-1.23258500	-0.95250100	3.00067300
C	-2.26308900	0.69827100	3.98276000
H	-1.48420900	0.78867500	4.75544400
C	-3.28417900	1.27343300	1.26855500
H	-2.51527700	1.91066000	0.80751600
H	-4.09863500	1.15478200	0.54027400
C	-4.47947000	-0.30380200	3.41113300
H	-5.30075700	-0.94234200	3.77083700
C	-5.01625600	1.08191200	3.04757800
H	-5.47050200	1.55044900	3.93567500
H	-5.80616000	0.99910900	2.28188300
C	-3.86437000	1.94226900	2.52292300
H	-4.23942800	2.93668400	2.23658200
C	-3.85250200	-0.98401400	2.18152700
H	-4.61706500	-1.14672600	1.40053500
H	-3.46372100	-1.96296300	2.49970300
C	-2.78644800	2.08558900	3.59981800

H	-3.20426700	2.58823000	4.48698100
H	-1.95959000	2.71545800	3.22649200
C	-3.41258100	-0.17010300	4.50160200
H	-3.03585100	-1.16551500	4.79229100
H	-3.85116400	0.28883400	5.40226400
C	-2.62480800	-1.05718500	-1.48509200
C	-2.71531000	-0.17224300	-4.29186900
H	-3.09446400	-0.23664100	-5.32445300
H	-1.98477200	0.65541900	-4.26925000
C	-4.19747500	-2.33472300	-2.98213200
H	-4.92176100	-3.16352700	-2.99494600
C	-3.36412300	0.23164500	-1.88637400
H	-4.23909900	0.36043600	-1.23008000
H	-2.72101500	1.11603900	-1.76011800
C	-2.03683800	-1.48893900	-3.90196400
H	-1.20004100	-1.69857900	-4.58554300
C	-3.62865000	-2.22528800	-1.55663700
H	-3.12612800	-3.17450300	-1.31712100
H	-4.44772600	-2.07218300	-0.83129700
C	-3.87257500	0.11176200	-3.33051600
H	-4.35967400	1.06026700	-3.60319900
C	-3.05124900	-2.63428400	-3.95155000
H	-2.56316600	-3.58690400	-3.68435300
H	-3.44270300	-2.74354900	-4.97571900
C	-1.47887200	-1.35466900	-2.47904600
H	-0.73216200	-0.53771500	-2.46016900
H	-0.96241400	-2.28573200	-2.17739700
C	-4.88798600	-1.03208600	-3.39082600
H	-5.29439800	-1.12917100	-4.41054300
H	-5.73732700	-0.82007200	-2.71939300
Au	0.34133500	0.09138300	-0.07078900
N	1.12009500	-1.89861900	0.50934200
P	-1.79058200	-0.88068800	0.19048400
C	-0.27022500	1.98428700	-0.50767900
C	-0.25275600	2.94263700	0.50635900
C	-0.56327700	2.38079300	-1.81811800
C	-0.57032100	4.27869900	0.23661700
C	-0.87836800	3.70777400	-2.09531900
C	-0.89216100	4.66889700	-1.07144600
H	-0.55054100	5.00293100	1.05097100
H	-1.11161000	4.03327100	-3.11028100
H	-0.54956000	1.66175000	-2.64129700
C	2.29078200	0.83842200	-0.77908500
C	2.55425800	1.17335200	0.39438500
H	0.00924500	2.67139100	1.53341200
O	-1.21384900	5.92314200	-1.43784000
C	-1.19500100	6.93853400	-0.45344500

H	-1.92616000	6.73390400	0.34651500
H	-0.19111900	7.04661800	-0.01066300
H	-1.46764400	7.86931500	-0.96172600
C	2.78986800	1.60010700	1.76744400
H	2.97209100	2.68362200	1.78609800
H	1.91803500	1.36974300	2.40263000
H	3.67401500	1.05977900	2.14098400
C	2.58218500	0.74754900	-2.22229300
H	2.40963800	-0.26656500	-2.60889500
H	1.96682300	1.47230200	-2.77535500
H	3.65200700	0.97557700	-2.33530900
C	-1.29090700	-2.56163700	0.68491000
O	5.05540000	1.35631100	-0.12492200
S	5.27310900	-0.11511700	-0.01302100
O	4.80199200	-0.69087300	1.28009300
O	4.85372200	-0.89236400	-1.20734000
C	7.10737900	-0.30276200	0.05296800
F	7.58836100	0.35435100	1.10143600
F	7.64964100	0.18445600	-1.05567600
F	7.41918700	-1.58897100	0.16377200

104

TS1-Z-Me E(MN15(SMD)/cc-pVTZ(pp))=-3084.28921374 imaginary frequency= -133.6769

C	-2.25055200	-3.55611500	0.96812000
H	-3.31055700	-3.31556700	0.90272500
C	-1.86983300	-4.84694200	1.31571500
H	-2.62939100	-5.59952200	1.53025100
C	-0.51178400	-5.16808600	1.38068300
H	-0.19474700	-6.17666700	1.64958300
C	0.44610800	-4.19871700	1.10335500
H	1.50253400	-4.46284900	1.16604200
C	0.06786400	-2.89422400	0.75614200
C	2.04703200	-2.37321200	-0.56152800
H	2.46671900	-3.35402600	-0.29633600
H	2.89315200	-1.67690200	-0.66696300
H	1.48789600	-2.46259700	-1.50476700
C	1.89552000	-1.69901000	1.77209400
H	1.25276600	-1.20154600	2.51437300
H	2.80955400	-1.12524600	1.55635500
H	2.21342800	-2.67283000	2.17237200
C	-2.70863000	-0.10894800	1.63375800
C	-1.64284400	0.04172600	2.74303300
H	-0.80582300	0.66867200	2.37862900
H	-1.23258500	-0.95250100	3.00067300
C	-2.26308900	0.69827100	3.98276000
H	-1.48420900	0.78867500	4.75544400
C	-3.28417900	1.27343300	1.26855500
H	-2.51527700	1.91066000	0.80751600

H	-4.09863500	1.15478200	0.54027400
C	-4.47947000	-0.30380200	3.41113300
H	-5.30075700	-0.94234200	3.77083700
C	-5.01625600	1.08191200	3.04757800
H	-5.47050200	1.55044900	3.93567500
H	-5.80616000	0.99910900	2.28188300
C	-3.86437000	1.94226900	2.52292300
H	-4.23942800	2.93668400	2.23658200
C	-3.85250200	-0.98401400	2.18152700
H	-4.61706500	-1.14672600	1.40053500
H	-3.46372100	-1.96296300	2.49970300
C	-2.78644800	2.08558900	3.59981800
H	-3.20426700	2.58823000	4.48698100
H	-1.95959000	2.71545800	3.22649200
C	-3.41258100	-0.17010300	4.50160200
H	-3.03585100	-1.16551500	4.79229100
H	-3.85116400	0.28883400	5.40226400
C	-2.62480800	-1.05718500	-1.48509200
C	-2.71531000	-0.17224300	-4.29186900
H	-3.09446400	-0.23664100	-5.32445300
H	-1.98477200	0.65541900	-4.26925000
C	-4.19747500	-2.33472300	-2.98213200
H	-4.92176100	-3.16352700	-2.99494600
C	-3.36412300	0.23164500	-1.88637400
H	-4.23909900	0.36043600	-1.23008000
H	-2.72101500	1.11603900	-1.76011800
C	-2.03683800	-1.48893900	-3.90196400
H	-1.20004100	-1.69857900	-4.58554300
C	-3.62865000	-2.22528800	-1.55663700
H	-3.12612800	-3.17450300	-1.31712100
H	-4.44772600	-2.07218300	-0.83129700
C	-3.87257500	0.11176200	-3.33051600
H	-4.35967400	1.06026700	-3.60319900
C	-3.05124900	-2.63428400	-3.95155000
H	-2.56316600	-3.58690400	-3.68435300
H	-3.44270300	-2.74354900	-4.97571900
C	-1.47887200	-1.35466900	-2.47904600
H	-0.73216200	-0.53771500	-2.46016900
H	-0.96241400	-2.28573200	-2.17739700
C	-4.88798600	-1.03208600	-3.39082600
H	-5.29439800	-1.12917100	-4.41054300
H	-5.73732700	-0.82007200	-2.71939300
Au	0.34133500	0.09138300	-0.07078900
N	1.12009500	-1.89861900	0.50934200
P	-1.79058200	-0.88068800	0.19048400
C	-0.27022500	1.98428700	-0.50767900
C	-0.25275600	2.94263700	0.50635900

C	-0.56327700	2.38079300	-1.81811800
C	-0.57032100	4.27869900	0.23661700
C	-0.87836800	3.70777400	-2.09531900
C	-0.89216100	4.66889700	-1.07144600
H	-0.55054100	5.00293100	1.05097100
H	-1.11161000	4.03327100	-3.11028100
H	-0.54956000	1.66175000	-2.64129700
C	2.29078200	0.83842200	-0.77908500
C	2.55425800	1.17335200	0.39438500
H	0.00924500	2.67139100	1.53341200
O	-1.21384900	5.92314200	-1.43784000
C	-1.19500100	6.93853400	-0.45344500
H	-1.92616000	6.73390400	0.34651500
H	-0.19111900	7.04661800	-0.01066300
H	-1.46764400	7.86931500	-0.96172600
C	2.78986800	1.60010700	1.76744400
H	2.97209100	2.68362200	1.78609800
H	1.91803500	1.36974300	2.40263000
H	3.67401500	1.05977900	2.14098400
C	2.58218500	0.74754900	-2.22229300
H	2.40963800	-0.26656500	-2.60889500
H	1.96682300	1.47230200	-2.77535500
H	3.65200700	0.97557700	-2.33530900
C	-1.29090700	-2.56163700	0.68491000
O	5.05540000	1.35631100	-0.12492200
S	5.27310900	-0.11511700	-0.01302100
O	4.80199200	-0.69087300	1.28009300
O	4.85372200	-0.89236400	-1.20734000
C	7.10737900	-0.30276200	0.05296800
F	7.58836100	0.35435100	1.10143600
F	7.64964100	0.18445600	-1.05567600
F	7.41918700	-1.58897100	0.16377200

104

INT2-E-Me E(MN15(SMD)/cc-pVTZ(pp))=-3084.32237752

C	-2.81568600	-3.42128100	-0.41744400
H	-3.79248500	-3.05544000	-0.10323900
C	-2.67563900	-4.76160400	-0.76062500
H	-3.53677100	-5.42941700	-0.71418600
C	-1.42685000	-5.24025200	-1.16330800
H	-1.29832100	-6.28767500	-1.43986800
C	-0.33454400	-4.37978700	-1.20006400
H	0.63897000	-4.77428300	-1.49541800
C	-0.47076600	-3.02902100	-0.84878900
C	1.40020300	-2.19971600	-2.16723700
H	1.61143700	-3.23034300	-2.48833600
H	2.35459800	-1.66408100	-2.07954700
H	0.74874300	-1.71309900	-2.90773000

C	1.68614100	-2.71576500	0.17611200
H	1.22477200	-2.65726400	1.17145400
H	2.60913600	-2.12585300	0.13289500
H	1.93257000	-3.76543300	-0.04650700
C	-2.40335900	-0.74695700	1.80109600
C	-1.53992300	-1.79774500	2.53059000
H	-0.46850000	-1.57800500	2.34534100
H	-1.74680600	-2.80734700	2.13833700
C	-1.82860200	-1.76376200	4.03743300
H	-1.20667000	-2.52760900	4.52946300
C	-2.04589600	0.63192800	2.38898500
H	-0.96967900	0.80805100	2.23693000
H	-2.58049100	1.44086900	1.86589100
C	-4.17002700	-1.01846800	3.56783100
H	-5.23639900	-1.24060300	3.72737800
C	-3.84141400	0.37099800	4.12033200
H	-4.07190000	0.41279000	5.19738300
H	-4.46454300	1.13376700	3.62266700
C	-2.35772500	0.66906300	3.88958600
H	-2.11403300	1.67318700	4.27013400
C	-3.89074000	-1.04487700	2.05743400
H	-4.52142800	-0.29144000	1.55734600
H	-4.16332700	-2.03452200	1.65554300
C	-1.49958800	-0.37955000	4.60131800
H	-1.69326300	-0.35940800	5.68623700
H	-0.42815600	-0.15403600	4.45521800
C	-3.31089800	-2.07490400	4.26866600
H	-3.55290100	-3.07961600	3.88150100
H	-3.52861300	-2.08044200	5.34910800
C	-3.09318600	-0.00822100	-1.21268900
C	-3.51752700	2.20087000	-3.11640800
H	-4.11562100	2.73294700	-3.87413900
H	-2.58703600	2.77669800	-2.97259700
C	-5.26245700	-0.06814300	-2.49103900
H	-6.19089700	-0.64398800	-2.62737100
C	-3.44747700	1.41338900	-0.73463800
H	-4.02684700	1.35469100	0.20253700
H	-2.53002100	1.98788600	-0.52323000
C	-3.19977600	0.78433000	-3.60746800
H	-2.63551200	0.83252400	-4.55177800
C	-4.39179800	-0.80002000	-1.45616700
H	-4.14957900	-1.79759400	-1.85557900
H	-4.95505100	-0.93294100	-0.51782700
C	-4.29351400	2.12513000	-1.79817800
H	-4.51816600	3.14176800	-1.44082700
C	-4.50303200	0.00953000	-3.81813700
H	-4.28637800	-1.00563700	-4.19224000

H	-5.12120200	0.51426200	-4.57845900
C	-2.33915100	0.06661800	-2.55885500
H	-1.38614600	0.60925700	-2.42769000
H	-2.09416500	-0.95781000	-2.89876900
C	-5.59388600	1.34418200	-2.00277200
H	-6.23252600	1.85539400	-2.74155200
H	-6.16054200	1.29825800	-1.05679400
Au	0.36650200	-0.01810200	-0.36492100
N	0.73427300	-2.18357300	-0.83714700
P	-1.89021000	-0.77439500	-0.00131000
C	0.21686400	1.99013000	-0.06706400
C	0.51664000	2.58372300	1.15874800
C	-0.13472900	2.81619200	-1.14552800
C	0.39421400	3.96664600	1.34062300
C	-0.25988500	4.19141000	-0.97573800
C	-0.00823500	4.77999300	0.27365400
H	0.62962200	4.39477000	2.31500200
H	-0.54191300	4.84113400	-1.80611400
H	-0.30748500	2.39538300	-2.13969200
C	2.30082500	0.53960000	-0.75624500
C	3.21549100	0.42430700	0.21498400
H	0.86151100	1.97988800	2.00195100
O	-0.16237200	6.11885100	0.34473400
C	0.12234200	6.76028600	1.57026100
H	-0.53707100	6.39702800	2.37668200
H	1.17354300	6.60887200	1.86781100
H	-0.05707200	7.82922000	1.41243500
C	3.05590200	0.02957100	1.64634100
H	3.41280600	0.83772900	2.30398400
H	2.00238200	-0.18512100	1.88632100
H	3.66003100	-0.86496200	1.86847100
C	2.59671000	1.06291200	-2.13429300
H	2.02146300	0.53071000	-2.90830000
H	2.30490200	2.12424000	-2.19115200
H	3.66419400	0.99895900	-2.39072900
C	-1.72434000	-2.53068100	-0.46786400
O	4.56546000	0.75737200	-0.09323800
S	5.47118900	-0.41974200	-0.76037400
O	4.72731100	-1.67783100	-0.71043700
O	6.07890100	0.08482500	-1.97567100
C	6.76948100	-0.47503600	0.56217300
F	6.18528600	-0.78355100	1.71173900
F	7.34577200	0.70474200	0.64979900
F	7.65093900	-1.40067100	0.24171000

104

INT2-Z-Me E(MN15(SMD)/cc-pVTZ(pp))=-3084.32117653

C	-2.74313600	-3.27427200	-0.62010800

H	-3.38519700	-3.11517600	0.24638400
C	-2.89195300	-4.43484700	-1.37155900
H	-3.65508000	-5.16586800	-1.10152900
C	-2.05190500	-4.65736300	-2.46548200
H	-2.15404300	-5.56250900	-3.06581500
C	-1.06986800	-3.72564000	-2.78658100
H	-0.41296800	-3.91915700	-3.63633800
C	-0.91678800	-2.55594500	-2.02882700
C	-0.11788300	-1.09007600	-3.76482500
H	-0.22771600	-1.91598200	-4.48460700
H	0.72792700	-0.46326900	-4.07153200
H	-1.04205300	-0.49346100	-3.75232500
C	1.46880300	-2.29824100	-2.39171900
H	1.64987800	-2.72548200	-1.39744100
H	2.24908200	-1.55970000	-2.61996400
H	1.50097800	-3.09530300	-3.15083300
C	-1.00952900	-1.47280400	1.78288100
C	-0.14019700	-2.73145300	1.58249200
H	0.69521000	-2.50104900	0.89805600
H	-0.73775200	-3.53995400	1.13080200
C	0.40039400	-3.21104000	2.93792800
H	1.01969200	-4.10477800	2.76506300
C	-0.12299300	-0.39686500	2.43742500
H	0.74670600	-0.23033800	1.77777200
H	-0.66276000	0.56162300	2.52659700
C	-1.64875400	-2.31988200	4.05881100
H	-2.50638300	-2.56808500	4.70321600
C	-0.81736600	-1.20978800	4.70790500
H	-0.45898200	-1.53766300	5.69741400
H	-1.44162200	-0.31323200	4.86637500
C	0.37371000	-0.87424000	3.80649400
H	0.97213600	-0.06903400	4.26140400
C	-2.18697700	-1.82227500	2.70868200
H	-2.82431800	-0.93937600	2.87776500
H	-2.81432900	-2.60724800	2.25283800
C	1.24258500	-2.11751000	3.60106800
H	1.62354100	-2.47878900	4.57078800
H	2.11088300	-1.87063400	2.96962900
C	-0.78237900	-3.56464000	3.84483300
H	-1.38369000	-4.37095200	3.39032400
H	-0.41583000	-3.93755500	4.81533100
C	-3.07448700	0.18290500	0.02051600
C	-4.22025600	2.83032800	-0.58618300
H	-5.07335400	3.52004100	-0.69523500
H	-3.30388300	3.40201300	-0.81064300
C	-5.58326000	0.35431300	0.18068800
H	-6.49412900	-0.22028200	0.40982800

C	-2.95405200	1.36296300	1.00744300
H	-2.92503600	0.98343800	2.04282400
H	-2.01796400	1.91906000	0.83308300
C	-4.37203100	1.66046700	-1.56429300
H	-4.40411000	2.03848800	-2.59824300
C	-4.37717300	-0.59118900	0.29978500
H	-4.49616800	-1.39435500	-0.44507100
H	-4.35985400	-1.05020200	1.30065600
C	-4.16225200	2.29573000	0.84823300
H	-4.04809200	3.13470900	1.55175000
C	-5.65798400	0.89252600	-1.25026300
H	-5.78933200	0.06012900	-1.96269200
H	-6.52942500	1.55873900	-1.35948600
C	-3.16452300	0.72363200	-1.42361200
H	-2.24108200	1.27264300	-1.67743700
H	-3.25603800	-0.12647100	-2.12659600
C	-5.44181700	1.51784300	1.16543600
H	-6.31884600	2.18137700	1.08944800
H	-5.40758900	1.13835500	2.20133400
Au	0.28216500	0.23401700	-1.12298800
N	0.15047600	-1.61331000	-2.40086700
P	-1.50303600	-0.83126900	0.08868300
C	0.33183400	2.07749100	-0.25627200
C	1.00464100	2.36882100	0.92921900
C	-0.36279400	3.10788700	-0.91064900
C	0.92102200	3.64383500	1.50388800
C	-0.45236700	4.37551900	-0.34472800
C	0.17693000	4.65243800	0.87913800
H	1.45145200	3.83562000	2.43676500
H	-1.00083200	5.17955400	-0.83880900
H	-0.84679800	2.93188600	-1.87561100
C	1.80578700	1.13154300	-2.18260700
C	3.04322600	1.09676400	-1.67075900
H	1.61996300	1.61654000	1.42247400
O	0.02653000	5.90577300	1.35846200
C	0.66853900	6.23781300	2.57147800
H	0.30776900	5.60845100	3.40262400
H	1.76339800	6.13229000	2.48887400
H	0.42233800	7.28477300	2.77907300
C	4.30539200	1.71274100	-2.17127500
H	5.10156600	0.95839400	-2.25498300
H	4.15322800	2.17459000	-3.15433700
H	4.64584300	2.48876700	-1.46741300
C	1.48491700	1.92710200	-3.42574200
H	0.43550200	1.80453400	-3.73295500
H	1.64341400	3.00250600	-3.24616600
H	2.11681000	1.63362400	-4.27961900

C	-1.76643200	-2.31153400	-0.94249900
O	3.16740900	0.53326700	-0.36286900
S	3.85254000	-0.91851700	-0.16845200
O	2.91372200	-1.76092600	0.56522900
O	4.48777600	-1.35967100	-1.40209000
C	5.14605100	-0.35309200	1.03257700
F	4.53646200	0.13599100	2.09846200
F	5.87373500	0.58334800	0.45094700
F	5.89724100	-1.38183600	1.36401100

104

TS2-E-Me E(MN15(SMD)/cc-pVTZ(pp))=-3084.29929143 imaginary frequency= -355.9898

C	-2.86728100	-3.43647200	-0.59862400
H	-3.82699600	-3.06425600	-0.23792500
C	-2.77549600	-4.74972500	-1.05011000
H	-3.65702200	-5.39218400	-1.04208200
C	-1.54927000	-5.23410500	-1.51152900
H	-1.46145700	-6.26006000	-1.87236200
C	-0.42845800	-4.40871100	-1.49678000
H	0.53191600	-4.80348400	-1.83460500
C	-0.51160100	-3.08707500	-1.03761700
C	1.40723200	-2.20359900	-2.26337300
H	1.70177000	-3.19662300	-2.64257700
H	2.32261000	-1.61129000	-2.11879800
H	0.75343800	-1.71320200	-3.00010000
C	1.61482500	-2.85375900	0.05729200
H	1.09403800	-2.88460900	1.02467200
H	2.51034000	-2.22238500	0.12345600
H	1.92988900	-3.87737900	-0.21112700
C	-2.36670300	-0.87087500	1.77879700
C	-1.58978900	-2.01877600	2.45550600
H	-0.50586800	-1.87926700	2.26983300
H	-1.88024800	-2.98874900	2.01886500
C	-1.86627300	-2.02853900	3.96434300
H	-1.30673600	-2.86156200	4.41754000
C	-1.89276200	0.45007500	2.42281000
H	-0.80433200	0.55135300	2.26233000
H	-2.36813800	1.32034200	1.94198100
C	-4.14030800	-1.07398200	3.54849500
H	-5.22047700	-1.21268100	3.70978900
C	-3.69332600	0.25894300	4.15588300
H	-3.91403300	0.27381700	5.23577400
H	-4.25328100	1.09145500	3.69603100
C	-2.19044200	0.44459600	3.92648800
H	-1.86447100	1.40716200	4.35119600
C	-3.87130500	-1.05880000	2.03642100
H	-4.44318300	-0.23782600	1.57317400
H	-4.22185200	-2.00508900	1.59043900

C	-1.41911700	-0.70229900	4.58402500
H	-1.60362200	-0.71264000	5.67077100
H	-0.33341300	-0.56117900	4.43684800
C	-3.36762800	-2.22567000	4.19771200
H	-3.69435200	-3.18995500	3.77223200
H	-3.57828400	-2.25796000	5.27921600
C	-3.01293000	0.04071600	-1.19025400
C	-3.29082300	2.36635900	-2.98749400
H	-3.85793900	2.97489700	-3.71087000
H	-2.31792700	2.86433700	-2.83095200
C	-5.18903800	0.19925100	-2.44498600
H	-6.15998700	-0.29675300	-2.59811500
C	-3.24656500	1.46417200	-0.64721900
H	-3.80909700	1.41505100	0.30055700
H	-2.27751800	1.95332600	-0.43535400
C	-3.08040100	0.95488600	-3.54501900
H	-2.52211100	1.00579000	-4.49291000
C	-4.36655500	-0.64191500	-1.45590000
H	-4.19831100	-1.63649300	-1.89954800
H	-4.92987300	-0.77794200	-0.51822600
C	-4.05327000	2.28404600	-1.66200000
H	-4.20151400	3.29637200	-1.25505400
C	-4.43720200	0.28511100	-3.77588200
H	-4.29674500	-0.72441200	-4.19828700
H	-5.02379200	0.86740600	-4.50532700
C	-2.26308600	0.13482400	-2.53843500
H	-1.28095900	0.62122600	-2.38495000
H	-2.07567700	-0.88295000	-2.92956600
C	-5.40905500	1.60678100	-1.88384700
H	-6.01796500	2.19924900	-2.58627700
H	-5.96592000	1.55480800	-0.93226000
Au	0.35175800	-0.02187000	-0.34300400
N	0.70886700	-2.29179500	-0.96621400
P	-1.85819500	-0.85404300	-0.02343600
C	0.71524500	2.03519200	-0.11405100
C	0.75958900	2.59585700	1.17198000
C	0.25732300	2.84091600	-1.17596800
C	0.34984700	3.90868000	1.40064800
C	-0.15497800	4.14836600	-0.95310800
C	-0.11623000	4.69919700	0.33744500
H	0.40653200	4.30882200	2.41293300
H	-0.51069200	4.77491600	-1.77296900
H	0.21573200	2.44402400	-2.19237600
C	2.20589100	0.88363000	-0.66969500
C	3.15295400	0.57781200	0.24988500
H	1.12431400	2.01284500	2.01882900
O	-0.53056500	5.97455000	0.45685900

C	-0.47679300	6.58427900	1.73126900
H	-1.12104500	6.05760700	2.45508000
H	0.55554500	6.61278000	2.11750200
H	-0.84217200	7.60849900	1.60170600
C	3.00962500	0.15487700	1.67196000
H	3.41050500	0.92690000	2.34981300
H	1.95872100	-0.05226200	1.92759400
H	3.59112300	-0.76491600	1.84323100
C	2.57848800	1.24664000	-2.08707500
H	1.78133700	0.96334800	-2.79235500
H	2.74422800	2.32975600	-2.18865000
H	3.50077800	0.73923300	-2.40278600
C	-1.74870200	-2.58049900	-0.59657700
O	4.49733800	0.80677300	-0.12284800
S	5.37532900	-0.44543100	-0.70095100
O	4.61190200	-1.67772400	-0.52266700
O	5.95672900	-0.05573500	-1.96952100
C	6.69844200	-0.39156100	0.59895600
F	6.12685900	-0.56629100	1.78166300
F	7.29812800	0.77808600	0.55248100
F	7.55314600	-1.36461000	0.36049700

104

TS2-Z-Me E(MN15(SMD)/cc-pVTZ(pp))=-3084.29850501 imaginary frequency= -350.7564

C	-2.65541800	-3.35369900	-0.58732800
H	-3.19609100	-3.23784500	0.35411200
C	-2.88352900	-4.48039600	-1.37112500
H	-3.60971400	-5.22962200	-1.05305300
C	-2.16890100	-4.64501300	-2.56038400
H	-2.33431200	-5.52309900	-3.18658100
C	-1.23432400	-3.68826400	-2.94560500
H	-0.67588000	-3.82829800	-3.87356400
C	-0.99879300	-2.55122000	-2.15969900
C	-0.47489000	-0.91856000	-3.84656500
H	-0.67746100	-1.65344400	-4.64539700
H	0.30738700	-0.23069700	-4.19510300
H	-1.39466100	-0.35238100	-3.63780300
C	1.30430400	-2.21633400	-2.86163900
H	1.58449300	-2.82945900	-1.99733500
H	2.06186200	-1.42735100	-2.99480900
H	1.28469100	-2.84086800	-3.77151900
C	-0.79841000	-1.57009800	1.75673600
C	-0.03367600	-2.88899600	1.51905300
H	0.73604700	-2.74337200	0.74350600
H	-0.72814800	-3.66445600	1.15757300
C	0.60665100	-3.36708400	2.82909500
H	1.15250400	-4.30199700	2.62779900
C	0.19323600	-0.52149400	2.30741900

H	0.99595500	-0.36911100	1.56645600
H	-0.31356800	0.44991900	2.44976700
C	-1.26269900	-2.32104600	4.11208000
H	-2.06769700	-2.49757200	4.84240200
C	-0.30338800	-1.25384300	4.64874100
H	0.13056100	-1.58778300	5.60564800
H	-0.85126800	-0.31617700	4.84659100
C	0.80689300	-1.00826100	3.62384400
H	1.49899900	-0.23521100	3.99525600
C	-1.89791700	-1.82935200	2.80142400
H	-2.47071000	-0.90940800	3.00181400
H	-2.60692800	-2.58982400	2.42987300
C	1.57424900	-2.30750300	3.36148700
H	2.04639000	-2.66351500	4.29226600
H	2.37662800	-2.13150600	2.62643300
C	-0.49607500	-3.62210600	3.86016500
H	-1.18399000	-4.40376900	3.49456700
H	-0.05674700	-3.98773700	4.80299600
C	-2.94340200	0.10788700	0.13312500
C	-4.09897800	2.76781500	-0.43347600
H	-4.95004700	3.46769100	-0.47263400
H	-3.20369000	3.31902900	-0.76730700
C	-5.41924000	0.33835700	0.53084900
H	-6.31573500	-0.20629500	0.86556000
C	-2.69901400	1.31550300	1.06162900
H	-2.56294000	0.97273200	2.10117300
H	-1.77330400	1.84082100	0.76482600
C	-4.36222200	1.57558900	-1.35818800
H	-4.48895000	1.92643200	-2.39459100
C	-4.22645600	-0.63000900	0.55688400
H	-4.42772300	-1.45388400	-0.14760700
H	-4.12058600	-1.06128800	1.56383000
C	-3.89985600	2.26855400	1.00073400
H	-3.70173500	3.12338600	1.66634300
C	-5.62427600	0.84117600	-0.89993500
H	-5.83838600	-0.00690200	-1.57253900
H	-6.49041600	1.52188000	-0.94338500
C	-3.15896400	0.62464300	-1.30675700
H	-2.25313800	1.16115400	-1.64938900
H	-3.32088000	-0.23431700	-1.98480900
C	-5.15404400	1.52241200	1.46568600
H	-6.02182400	2.20241400	1.46422400
H	-5.01993700	1.16572900	2.50169200
Au	0.28881900	0.28209400	-1.11577500
N	-0.00561900	-1.58202000	-2.61437500
P	-1.38491200	-0.92599700	0.09033500
C	0.66195200	2.26744800	-0.53422600

C	1.26790200	2.48214900	0.71553200
C	-0.24025700	3.23645200	-1.01550600
C	0.98867600	3.62917700	1.45746500
C	-0.51757100	4.38128000	-0.27825100
C	0.09250900	4.59321800	0.96724600
H	1.47594400	3.75890900	2.42388800
H	-1.21332100	5.13588800	-0.64924400
H	-0.74644500	3.09800200	-1.97249700
C	1.67992300	1.51982000	-2.05896200
C	2.99688100	1.31327600	-1.81414000
H	1.95957400	1.74893000	1.12721800
O	-0.23630800	5.72970900	1.61055200
C	0.37235000	5.99758400	2.85814500
H	0.11745700	5.22505600	3.60277500
H	1.46897600	6.06144500	2.76276700
H	-0.01791200	6.96435100	3.19349700
C	4.17896000	1.74107000	-2.61675500
H	4.85073100	0.89146300	-2.80705600
H	3.88107400	2.18612800	-3.57246600
H	4.74986200	2.49195900	-2.04582100
C	1.25590500	2.17603600	-3.35353300
H	0.18344300	2.03475000	-3.54955700
H	1.44869300	3.25975200	-3.33301700
H	1.80252900	1.74518200	-4.20539100
C	-1.72233400	-2.36980300	-0.96748400
O	3.35134200	0.83104800	-0.52855600
S	3.71174100	-0.74261100	-0.36606100
O	2.50265100	-1.50315000	-0.05556400
O	4.61765600	-1.17325800	-1.41793000
C	4.60349900	-0.48720000	1.24377500
F	3.77736400	0.12804400	2.08014100
F	5.67903600	0.24011600	1.04400900
F	4.92032600	-1.67540200	1.71796900

33

prod-E-Me E(MN15(SMD)/cc-pVTZ(pp))=-1462.85322858

C	-1.92846400	0.66027900	0.18392300
C	-2.32119400	-0.51139100	-0.47163700
C	-2.93084900	1.46285900	0.75793500
C	-3.66301800	-0.89662700	-0.54450600
C	-4.26640100	1.09020500	0.70021400
C	-4.64482400	-0.09406500	0.04790600
H	-3.92727300	-1.81410900	-1.07034300
H	-5.04992200	1.69928500	1.15372100
H	-2.65567100	2.38639600	1.27335300
C	-0.49802900	1.06757200	0.21730300
C	0.43566700	0.23166500	0.70031000
H	-1.55632800	-1.12856600	-0.95043700

O	-5.97213800	-0.37287300	0.03839900
C	-6.39350600	-1.54933000	-0.61094000
H	-6.13348100	-1.53566700	-1.68349000
H	-5.94907800	-2.44819600	-0.14963300
H	-7.48370100	-1.59340800	-0.50617700
C	0.29257900	-1.11371300	1.32771600
H	0.88604800	-1.15082600	2.25409800
H	-0.75570100	-1.33440700	1.55947800
H	0.68329700	-1.89330300	0.65364300
C	-0.16358300	2.41761000	-0.35660600
H	-0.41692200	2.43929300	-1.42837500
H	-0.77247900	3.19876800	0.12552300
H	0.89623900	2.67372000	-0.24003500
O	1.79038200	0.66586300	0.66753800
S	2.65495800	0.28237500	-0.65231800
O	1.89494700	-0.62946300	-1.49525400
O	3.29742400	1.48535400	-1.14929500
C	3.94148300	-0.70872600	0.23396600
F	3.35426600	-1.71984900	0.85992800
F	4.56584000	0.05727900	1.10975600
F	4.80339800	-1.17588100	-0.65400100

33

prod-Z-Me E(MN15(SMD)/cc-pVTZ(pp))=-1462.85198297

C	-1.21199900	1.38128000	-0.04460700
C	-1.75057200	0.75535300	-1.17179200
C	-1.78695500	1.10862600	1.20853000
C	-2.83224700	-0.12148900	-1.07008300
C	-2.85752000	0.23335100	1.32549300
C	-3.38814100	-0.39024900	0.18623500
H	-3.22225900	-0.59539700	-1.97064900
H	-3.30101500	-0.00178900	2.29413500
H	-1.35999700	1.56200200	2.10645200
C	-0.02610700	2.27975300	-0.14583700
C	1.15655000	1.77811700	-0.53438400
H	-1.30256300	0.94415800	-2.14994700
O	-4.43080300	-1.23189300	0.39761900
C	-4.96307000	-1.91265600	-0.71375200
H	-5.37670000	-1.21170000	-1.45964200
H	-4.20211300	-2.54414400	-1.20365100
H	-5.77057400	-2.55012000	-0.33515800
C	2.49176400	2.42666200	-0.66911600
H	2.92673700	2.19621600	-1.65352000
H	2.43532500	3.51485300	-0.55513200
H	3.17530500	2.02390400	0.09506200
C	-0.23337300	3.71402300	0.24883700
H	-0.98696500	4.18031100	-0.40495200
H	-0.63568700	3.76263900	1.27356700

H	0.68453000	4.31346700	0.21392000
O	1.22124000	0.39857100	-0.86548900
S	1.18669100	-0.68297500	0.35358400
O	1.31923000	0.00385500	1.63013100
O	0.18226100	-1.69048300	0.07262500
C	2.83271700	-1.43284800	-0.03567700
F	3.77003000	-0.49635700	0.04145500
F	2.81408900	-1.94350600	-1.25440400
F	3.08557200	-2.38533100	0.84739300

28

di-t-buylacetylene E(MN15(SMD)/cc-pVTZ(pp))=-391.478839866

C	0.61034100	0.00037900	0.00036700
C	-0.61063100	0.00029700	0.00022100
C	-2.08723300	0.00005400	-0.00007400
C	2.08737000	0.00047800	0.00054300
C	-2.59615100	1.09892600	0.94420700
H	-2.24178800	2.08851100	0.61922200
H	-3.69821000	1.10634700	0.95017700
H	-2.24231100	0.92601900	1.97147000
C	-2.59567700	0.26814600	-1.42407900
H	-2.23977300	1.24313500	-1.78892400
H	-2.24299500	-0.50950900	-2.11778500
H	-3.69777400	0.27196100	-1.43370800
C	2.59552100	-1.16260900	0.86501200
H	2.23879000	-1.06363300	1.90123600
H	2.24262700	-2.12688600	0.46927300
H	3.69785400	-1.16964900	0.87308800
C	2.59599900	-0.16879600	-1.43836800
H	2.23957800	-1.11626600	-1.86984600
H	2.24348000	0.65535700	-2.07682600
H	3.69832400	-0.17217900	-1.44784800
C	2.59508600	1.33154800	0.57319800
H	3.69796000	1.34151200	0.57485700
H	2.23829100	2.17870900	-0.03259000
H	2.24212300	1.47256600	1.60640600
C	-2.59457400	-1.36785700	0.47954600
H	-2.23921800	-2.17088300	-0.18351000
H	-2.24041600	-1.58020800	1.49946000
H	-3.69684900	-1.37829700	0.48241300

122

INT1-tBu E(MN15(SMD)/cc-pVTZ(pp))=-3319.94286794

C	1.31776000	3.38806200	0.69341000
H	0.54918800	4.10516000	0.97728100
C	2.64682300	3.79675900	0.65615700
H	2.90483100	4.81749900	0.94161700
C	3.63819100	2.90355600	0.24195300
H	4.68621300	3.20160400	0.20445400

C	3.30465800	1.59986900	-0.11456400
H	4.08780700	0.90091000	-0.41983300
C	1.97190400	1.18329200	-0.04698300
C	2.37380300	-1.01202700	0.75465600
H	3.44922300	-0.76508100	0.78419000
H	2.24966400	-2.07886000	0.53659400
H	1.92396300	-0.75055200	1.72307200
C	2.22661800	-0.63261300	-1.64070700
H	1.80611600	0.02540600	-2.41467400
H	1.93713100	-1.67612500	-1.82732300
H	3.32919800	-0.58674300	-1.61585600
C	-1.54477600	2.49559600	-1.15327000
C	-1.04223000	1.80474800	-2.44015500
H	-1.31704100	0.73588000	-2.44546400
H	0.06311000	1.85655300	-2.46732800
C	-1.63842200	2.49592200	-3.67248000
H	-1.28290800	1.97140800	-4.57265900
C	-3.08155000	2.51457600	-1.07463900
H	-3.49543600	1.50911900	-0.91516600
H	-3.37819300	3.13098700	-0.20940400
C	-1.67420500	4.66253300	-2.43806800
H	-1.32610500	5.70667500	-2.43560000
C	-3.20222600	4.62146300	-2.38957700
H	-3.61854900	5.12441100	-3.27739000
H	-3.57476000	5.16142300	-1.50272000
C	-3.65713100	3.16120300	-2.34302300
H	-4.75540400	3.11220800	-2.28768100
C	-1.07072000	3.96523100	-1.20571200
H	-1.37286000	4.49807500	-0.28670800
H	0.02483300	4.01075500	-1.28733700
C	-3.16730100	2.42571100	-3.59482100
H	-3.60845700	2.88673400	-4.49321500
H	-3.50209600	1.37278500	-3.58046700
C	-1.18383800	3.95700300	-3.70494800
H	-0.08441300	4.01069900	-3.77287300
H	-1.59207600	4.45893600	-4.59690900
C	-1.51631900	1.63036800	1.98511600
C	-2.72396900	0.18638800	4.25137300
H	-3.10129100	0.12663000	5.28489500
H	-2.86257300	-0.81011300	3.79347900
C	-1.84224700	2.98148500	4.08178400
H	-1.69371900	3.98133500	4.51732000
C	-3.02108400	1.29708600	2.01156500
H	-3.58841700	2.08160000	1.49184600
H	-3.22411400	0.34481000	1.50131500
C	-1.23997200	0.56283400	4.25474000
H	-0.65938600	-0.18804700	4.81375000

C	-1.31017100	3.01228700	2.63756100
H	-0.23866100	3.25595200	2.67789800
H	-1.82832300	3.79372100	2.05200000
C	-3.51155800	1.23774800	3.46569600
H	-4.57908100	0.96983700	3.45317800
C	-1.04857500	1.94561600	4.88406100
H	0.02181400	2.21108500	4.89961700
H	-1.39753600	1.93340100	5.92912600
C	-0.73231300	0.58486300	2.80837000
H	-0.87041900	-0.41878500	2.36357600
H	0.34715800	0.82357700	2.78359700
C	-3.32727300	2.61663900	4.10340700
H	-3.69497000	2.61052300	5.14228600
H	-3.91635500	3.36951200	3.55261200
Au	-0.43982100	-0.73962400	-0.25200400
N	1.67424300	-0.23245500	-0.31115400
P	-0.77044100	1.53423400	0.26549800
C	-2.41918600	-1.25395900	-0.21598400
C	-3.32451500	-0.92788600	-1.22425700
C	-2.86356100	-2.08475800	0.82786200
C	-4.64256500	-1.40832300	-1.20859600
C	-4.16584100	-2.56340400	0.86094900
C	-5.07469100	-2.23010900	-0.16101900
H	-5.31482400	-1.13162000	-2.02089500
H	-4.51264300	-3.20941500	1.66937300
H	-2.18478500	-2.36761800	1.63383600
C	0.10380600	-3.23887300	0.15882200
C	-0.16991700	-2.89712000	-1.00666700
H	-3.03288600	-0.29143700	-2.06028100
O	-6.31204300	-2.74177000	-0.04540200
C	-7.25920600	-2.44985600	-1.05547800
H	-7.45061900	-1.36603500	-1.12142600
H	-6.92153500	-2.82096800	-2.03725100
H	-8.18367600	-2.96383300	-0.77263800
C	-0.49443700	-3.27079600	-2.43298000
C	0.37510500	-3.99714000	1.41034900
C	0.96107500	2.06891400	0.34212100
O	5.18855600	-0.84589700	-1.08552200
S	6.08065500	-0.80900200	0.11860600
O	5.35664400	-0.29414600	1.31586000
O	6.95291200	-1.96816700	0.30748700
C	7.19889500	0.60228000	-0.29299100
F	7.91328700	0.35280000	-1.38282500
F	8.02022600	0.87430400	0.71332800
F	6.45401800	1.70437300	-0.53019100
C	-0.66033200	-2.07720300	-3.37461500
H	-0.81667000	-2.45079900	-4.39695400

H	-1.53888000	-1.47735600	-3.09986100
H	0.22920600	-1.43102300	-3.38399500
C	0.66229000	-4.16358800	-2.91277300
H	0.79072600	-5.02953000	-2.24529900
H	0.43433800	-4.53510100	-3.92341900
H	1.61371300	-3.61267300	-2.95572600
C	-1.79647100	-4.08775600	-2.38278600
H	-2.63019700	-3.49159100	-1.98228800
H	-2.05447100	-4.41621900	-3.40147200
H	-1.66976100	-4.98030200	-1.75157900
C	0.29200300	-3.17895000	2.70567600
H	-0.71656200	-2.78054700	2.89118100
H	0.53807600	-3.84966300	3.54177100
H	1.01317600	-2.35194300	2.71875900
C	1.76682900	-4.64929900	1.29273300
H	1.88113400	-5.37378200	2.11318000
H	1.87150400	-5.18978500	0.34028600
H	2.58358500	-3.91910500	1.37407000
C	-0.69522100	-5.11095100	1.42698700
H	-0.54161700	-5.73529000	2.32012800
H	-1.71311500	-4.69524300	1.45136200
H	-0.60197800	-5.75399100	0.53856900

122

TS1-E-tBu E(MN15(SMD)/cc-pVTZ(pp))=-3319.92514572 imaginary frequency= -98.0881

C	-2.40221300	-3.77699600	-0.19124700
H	-3.45455500	-3.57224400	-0.00112500
C	-1.99380200	-5.09341200	-0.36699400
H	-2.72372500	-5.90167400	-0.30875700
C	-0.64717600	-5.36845900	-0.62203700
H	-0.31155800	-6.39542300	-0.77286500
C	0.27564900	-4.33028200	-0.66946200
H	1.32733100	-4.55606000	-0.84879800
C	-0.13179000	-3.00269900	-0.47074200
C	1.51654700	-1.85691300	-1.80845200
H	1.89869000	-2.84292700	-2.11408100
H	2.37720100	-1.17881200	-1.75049800
H	0.75933300	-1.51269300	-2.52821400
C	1.93193000	-2.34221700	0.54025700
H	1.43480600	-2.65705000	1.46813200
H	2.59823600	-1.49647900	0.71772300
H	2.56540900	-3.15142500	0.14894900
C	-2.81146200	-0.85252100	1.64386200
C	-1.95065300	-1.69056800	2.61179600
H	-0.89846300	-1.35609300	2.55610400
H	-1.97439000	-2.75281900	2.31889200
C	-2.48554000	-1.54898800	4.04347200
H	-1.84927700	-2.14855200	4.71245900

C	-2.77681900	0.61622800	2.10599500
H	-1.73438700	0.96628800	2.10352600
H	-3.33358700	1.26406000	1.40782600
C	-4.79526700	-1.24565600	3.13771500
H	-5.82797200	-1.62660700	3.15235900
C	-4.77786700	0.22397400	3.56393900
H	-5.19087700	0.32566900	4.58057300
H	-5.41448900	0.82314400	2.89066600
C	-3.33833900	0.74030300	3.52665400
H	-3.31229300	1.80213000	3.81633400
C	-4.26307400	-1.36990000	1.70006700
H	-4.90996000	-0.78929200	1.02371700
H	-4.31258000	-2.42626400	1.39208000
C	-2.46079700	-0.08065300	4.47567600
H	-2.82812700	0.01185500	5.51060000
H	-1.42515500	0.30342700	4.46038500
C	-3.92404500	-2.07339900	4.08658800
H	-3.94902200	-3.13773200	3.79653700
H	-4.31918600	-2.00570500	5.11307800
C	-3.11844800	-0.61001600	-1.51868000
C	-3.69655900	1.19496100	-3.76232200
H	-4.25835100	1.48108600	-4.66628600
H	-2.95490700	1.99319100	-3.58446500
C	-4.97864300	-1.33920500	-3.05999200
H	-5.71629500	-2.13853400	-3.22997000
C	-3.88029500	0.71168400	-1.29978500
H	-4.59822200	0.58482900	-0.47235900
H	-3.19999200	1.52282800	-1.00980300
C	-2.99759300	-0.14910800	-3.99581600
H	-2.30790300	-0.07033500	-4.85006000
C	-4.15480600	-1.71655200	-1.81561100
H	-3.64016400	-2.66658600	-2.02473300
H	-4.82691600	-1.86776300	-0.95523100
C	-4.65486700	1.08182700	-2.57187300
H	-5.15488200	2.04792400	-2.40399600
C	-4.04549900	-1.23100900	-4.26843100
H	-3.55157900	-2.19892800	-4.45837400
H	-4.62496600	-0.97578900	-5.17040600
C	-2.18857000	-0.53602300	-2.75009500
H	-1.36625400	0.18257200	-2.59016000
H	-1.72084500	-1.52743900	-2.90045900
C	-5.69628200	-0.00537400	-2.84506800
H	-6.28937500	0.24824000	-3.73866200
H	-6.39768500	-0.07846500	-1.99639700
Au	0.11091400	0.12667900	-0.11560400
N	0.89266400	-1.95341500	-0.45615200
P	-2.00977500	-0.98105100	-0.05012000

C	-0.58086700	2.05993600	-0.01917600
C	-0.38040300	2.88176100	1.09138800
C	-1.15490800	2.63665900	-1.16125400
C	-0.75402100	4.23014000	1.08155100
C	-1.52941600	3.97633500	-1.18724100
C	-1.33426400	4.79289800	-0.06319200
H	-0.57395000	4.82976500	1.97373000
H	-1.97379100	4.41798200	-2.08113700
H	-1.31671700	2.05342700	-2.06607600
C	2.11079900	1.04013600	-0.28671600
C	2.55812800	0.71867800	0.86478200
H	0.08260400	2.50575700	2.00210200
O	-1.72606000	6.07493500	-0.17524600
C	-1.51511400	6.94065900	0.92355800
H	-2.07053500	6.60020500	1.81326200
H	-0.44345500	7.01759400	1.17080700
H	-1.88760200	7.92464600	0.62007800
C	2.67331200	0.70459600	2.35565100
C	2.50281700	1.86348200	-1.52598100
C	-1.48032300	-2.71103300	-0.25580600
O	4.84971700	0.42668300	0.54409200
S	5.18572700	-0.95036600	0.02384000
O	5.29048000	-1.99478700	1.05626400
O	4.39668600	-1.33839000	-1.17525200
C	6.89218000	-0.67352900	-0.62416400
F	6.86138000	0.29070500	-1.54307200
F	7.35025100	-1.78676100	-1.17925800
F	7.69904100	-0.30708200	0.36187200
C	3.71150400	-0.23300100	2.99050700
H	4.73226800	0.09107200	2.77081800
H	3.54205100	-0.20586000	4.07848100
H	3.61710700	-1.27089800	2.64763900
C	1.31808400	0.28630900	2.96350200
H	0.44370700	0.78957800	2.53085600
H	1.18506600	-0.80040300	2.85403700
H	1.34430100	0.51236300	4.04040600
C	3.04159900	2.15813700	2.71863600
H	4.00044300	2.41298500	2.24309600
H	2.27778100	2.87534900	2.38256800
H	3.16162200	2.23473600	3.81031700
C	1.69754500	1.48639300	-2.77400900
H	2.03662500	0.52648400	-3.18779400
H	0.61466100	1.43515100	-2.58880200
H	1.86543700	2.25259200	-3.54548800
C	2.26633000	3.34062900	-1.15035800
H	1.20954400	3.59718600	-1.01211400
H	2.81888200	3.58895800	-0.23101300

H	2.66675400	3.96268400	-1.96623800
C	3.98850900	1.72896000	-1.88485800
H	4.63605700	2.09545000	-1.08071100
H	4.26629600	0.69163500	-2.10285600
H	4.15199500	2.34157300	-2.78620200

122

TS1-Z-tBu E(MN15(SMD)/cc-pVTZ(pp))=-3319.91399459 imaginary frequency= -155.9795

C	2.69899200	2.82190600	-1.62204500
H	3.49090900	2.82064000	-0.87172600
C	2.67786800	3.82047500	-2.58960100
H	3.45028900	4.59021800	-2.59821600
C	1.65300900	3.82798600	-3.53636700
H	1.61178600	4.60568400	-4.30035600
C	0.67384100	2.83878200	-3.51053000
H	-0.11322900	2.85716400	-4.26446400
C	0.69093700	1.83182000	-2.53797200
C	-0.01435200	-0.11330900	-3.74804100
H	0.10158500	0.49034300	-4.66233900
H	-0.85138300	-0.80951000	-3.88526500
H	0.90698800	-0.67368400	-3.56278600
C	-1.68376200	1.33929000	-2.92179900
H	-1.89574200	2.19646700	-2.28021500
H	-2.42996800	0.55277700	-2.75480900
H	-1.71338400	1.63281400	-3.98223400
C	2.00600000	1.49615600	1.43327800
C	1.34168400	2.88589900	1.42815400
H	0.28943500	2.80494600	1.14263000
H	1.82970200	3.54162900	0.68877100
C	1.44806500	3.53110800	2.81954800
H	0.94609900	4.50990400	2.77254200
C	1.29700300	0.63748800	2.49888500
H	0.22778000	0.59335300	2.24085100
H	1.68103200	-0.39852200	2.50425000
C	3.59364100	2.34382200	3.20302100
H	4.66325600	2.45260600	3.44254900
C	2.92310700	1.43042100	4.23266600
H	3.03319700	1.86071200	5.24154400
H	3.41694000	0.44252200	4.24238800
C	1.44158800	1.27729200	3.88319300
H	0.94645400	0.62096200	4.61618600
C	3.49026100	1.71040300	1.80528800
H	4.06189200	0.77432800	1.80783100
H	3.96991400	2.38801600	1.07864800
C	0.75691800	2.64638200	3.86058700
H	0.81937300	3.11889100	4.85511600
H	-0.30875100	2.52773400	3.60648700
C	2.91745400	3.71458000	3.19640400

H	3.41924000	4.38052500	2.47318900
H	3.00281200	4.18436600	4.19020900
C	3.18412000	-0.57818700	-0.61170200
C	4.08085200	-3.37385800	-1.00587800
H	4.84332000	-4.12861000	-1.25878500
H	3.15113200	-3.92063600	-0.76492100
C	5.58230100	-0.87472100	-1.33416700
H	6.50446500	-0.32329800	-1.57440100
C	3.44982900	-1.52844700	0.57302600
H	3.78066800	-0.97438100	1.46174600
H	2.52395500	-2.04310800	0.85949200
C	3.85165500	-2.44213000	-2.19768000
H	3.50502500	-3.02211300	-3.06765600
C	4.48720500	0.15324500	-0.99989700
H	4.30797100	0.78087800	-1.88850700
H	4.83846000	0.80812600	-0.19036500
C	4.53469900	-2.54639600	0.19913300
H	4.70044900	-3.20741300	1.06375800
C	5.15055600	-1.71010100	-2.54085600
H	5.00026500	-1.06035000	-3.41987200
H	5.93717000	-2.43738500	-2.80042000
C	2.77226300	-1.41303100	-1.83912200
H	1.81269100	-1.92820700	-1.65029200
H	2.63485700	-0.72409500	-2.69152300
C	5.82636200	-1.79548700	-0.13554200
H	6.63257000	-2.50963100	-0.37044700
H	6.15601700	-1.20641900	0.73772300
Au	-0.34751700	-0.50246800	-0.72286800
N	-0.33690200	0.77711600	-2.60004900
P	1.74355000	0.59010600	-0.22895200
C	-0.41211400	-1.72290400	0.91691000
C	-1.30906700	-1.39414500	1.93378800
C	0.32687300	-2.90596700	1.02298000
C	-1.44554800	-2.20855600	3.06194300
C	0.19433600	-3.72660300	2.14214800
C	-0.68934300	-3.38463900	3.17569400
H	-2.14686300	-1.91025400	3.84189700
H	0.77065200	-4.64828800	2.23699700
H	1.02072000	-3.21430300	0.23966000
C	-2.14045900	-1.58641100	-1.15243100
C	-3.23073400	-1.06081100	-0.67293200
H	-1.90629000	-0.48170100	1.87528300
O	-0.74738200	-4.23570300	4.22086700
C	-1.62407200	-3.92832300	5.28518700
H	-1.36151600	-2.96646800	5.75664000
H	-2.67203300	-3.88793700	4.94353800
H	-1.51569800	-4.73251300	6.02086700

C	-4.65941900	-0.94738200	-0.26667900
C	-2.06384600	-2.96789100	-1.86491800
C	1.71118300	1.82053300	-1.57415400
O	-2.76324500	0.80345400	-0.29690600
S	-2.33011700	2.03796900	0.48044100
O	-2.19290800	1.82638300	1.92778600
O	-1.23864200	2.72285000	-0.23893300
C	-3.77172400	3.17343300	0.23633200
F	-4.86478400	2.66982500	0.79782200
F	-3.98136700	3.30867500	-1.07311400
F	-3.50153600	4.35062200	0.76559900
C	-0.63651600	-3.31083500	-2.30898000
H	-0.18877300	-2.53233400	-2.93856200
H	0.01754800	-3.48531100	-1.44609900
H	-0.66644300	-4.24126200	-2.89624100
C	-4.78696800	-0.56575200	1.21887600
H	-4.28655700	-1.31630700	1.84950400
H	-4.36659800	0.41835700	1.44412000
H	-5.85632400	-0.55798800	1.47884900
C	-5.33101900	-2.33459500	-0.38729900
H	-6.39271100	-2.18285000	-0.14250700
H	-5.28327200	-2.76118900	-1.39721700
H	-4.91603100	-3.04662600	0.33791000
C	-5.40594400	0.02476300	-1.19119300
H	-4.96525200	1.02622100	-1.16682500
H	-5.38456900	-0.34400200	-2.22930100
H	-6.45712300	0.09223500	-0.87275100
C	-2.93808300	-2.90581100	-3.12696600
H	-3.98045500	-2.64556100	-2.89799000
H	-2.55967800	-2.15349100	-3.83768200
H	-2.92717500	-3.88349300	-3.63356100
C	-2.51744400	-4.10200900	-0.93294000
H	-2.02616700	-4.02284200	0.04928900
H	-3.60191200	-4.10847300	-0.78657000
H	-2.23847700	-5.06872500	-1.38077900

122

INT2-E-tBu E(MN15(SMD)/cc-pVTZ(pp))=-3319.93651588

C	-3.39488600	-3.20039300	0.62582300
H	-4.29292800	-2.66202000	0.92497300
C	-3.39227100	-4.58896900	0.68598100
H	-4.27911300	-5.12214100	1.03059600
C	-2.24695000	-5.28735700	0.29892200
H	-2.22489600	-6.37763200	0.32957000
C	-1.12278200	-4.58831800	-0.12693000
H	-0.23886700	-5.14923000	-0.43095700
C	-1.11711000	-3.18582200	-0.17008700
C	0.20855000	-2.69699500	-2.11017400

H	0.17920100	-3.77251800	-2.34963800
H	1.16494700	-2.27621900	-2.44129000
H	-0.61955900	-2.18743300	-2.61779600
C	1.30897600	-3.13194600	-0.03805200
H	1.16558300	-3.25190100	1.04279100
H	2.17939900	-2.49443600	-0.23866200
H	1.49658200	-4.11888400	-0.48699200
C	-2.77058500	-0.08286700	1.89796300
C	-2.04160900	-0.99333900	2.90687500
H	-0.96298900	-1.01242700	2.67978400
H	-2.41361100	-2.02741200	2.82069200
C	-2.27375000	-0.49277200	4.33930900
H	-1.73058700	-1.15578200	5.03081500
C	-2.25271000	1.35922100	2.08021100
H	-1.16932300	1.38125000	1.88709700
H	-2.71819600	2.04004400	1.34745500
C	-4.51627600	0.36501100	3.65914200
H	-5.59831000	0.32362500	3.85882000
C	-4.01806100	1.80523600	3.80108200
H	-4.21733600	2.17303000	4.82093700
H	-4.56021700	2.46583400	3.10279500
C	-2.51701200	1.84883300	3.50773900
H	-2.14659200	2.88215600	3.59300400
C	-4.27803800	-0.12581600	2.22115100
H	-4.85016000	0.50866600	1.52788400
H	-4.66725100	-1.15261900	2.13214000
C	-1.76467500	0.94347000	4.48665900
H	-1.91626600	1.29079000	5.52186800
H	-0.68009200	0.98559000	4.28138000
C	-3.77318900	-0.54018900	4.64477100
H	-4.14425400	-1.57641500	4.56441000
H	-3.95995300	-0.20477900	5.67805800
C	-3.53371600	-0.16678300	-1.16559700
C	-4.05462200	1.49029200	-3.54491200
H	-4.67555300	1.82182400	-4.39306500
H	-3.12075500	2.07964900	-3.57995500
C	-5.75178700	-0.55494200	-2.30663800
H	-6.67512300	-1.15251000	-2.25515200
C	-3.90743800	1.32483600	-1.04431200
H	-4.46532800	1.49353100	-0.10990600
H	-3.01073000	1.95616500	-0.99471800
C	-3.74483800	-0.00361400	-3.68037400
H	-3.21475200	-0.19220500	-4.62683200
C	-4.83613100	-0.99306300	-1.14972700
H	-4.60348900	-2.06218700	-1.27786000
H	-5.36648200	-0.87047000	-0.19121400
C	-4.79111600	1.74377800	-2.22618800

H	-5.01668400	2.81670900	-2.12670700
C	-5.04563200	-0.80916300	-3.64084600
H	-4.82909500	-1.88454200	-3.75982200
H	-5.70020400	-0.51184300	-4.47636000
C	-2.84612700	-0.44595200	-2.51900400
H	-1.86738200	0.06353000	-2.57901100
H	-2.65820600	-1.53368400	-2.59448300
C	-6.08813900	0.93281800	-2.18517600
H	-6.75466000	1.23467300	-3.00962800
H	-6.62536000	1.12880400	-1.24135000
Au	0.07685800	-0.23965300	-0.37161700
N	0.10088400	-2.50708500	-0.63867000
P	-2.28140800	-0.64307600	0.16197200
C	0.07963900	1.80908800	-0.34754800
C	0.82660000	2.56876600	0.55144700
C	-0.69536100	2.49506500	-1.29448700
C	0.76700100	3.96809900	0.55625300
C	-0.75772900	3.88405700	-1.30990900
C	-0.03415700	4.63962600	-0.37429700
H	1.36646600	4.51660100	1.28293100
H	-1.36073600	4.41317400	-2.05019500
H	-1.25017800	1.94874100	-2.05761700
C	2.11009100	0.01222100	-0.77023900
C	3.01770400	-0.09633500	0.23209100
H	1.48713100	2.08820300	1.26882100
O	-0.16100100	5.97946500	-0.45749300
C	0.58014800	6.77685500	0.44317300
H	0.29788500	6.56871100	1.48900300
H	1.66435400	6.61518400	0.32264900
H	0.34214700	7.81930900	0.20599600
C	2.92970200	-0.31850400	1.75160200
C	2.48476200	0.34736200	-2.24044700
C	-2.26727300	-2.47213000	0.19496600
O	4.38107200	0.24074800	-0.08680100
S	5.54750600	-0.74578700	-0.62001400
O	5.17452700	-2.13957100	-0.40084200
O	6.03445400	-0.25250500	-1.89677500
C	6.88822000	-0.21929600	0.57205000
F	6.75150300	-0.80287600	1.75265400
F	6.83072900	1.09285200	0.71731800
F	8.03680500	-0.57036700	0.03321900
C	3.66266800	-1.60200600	2.18723400
H	4.72762100	-1.57873800	1.94975900
H	3.57098400	-1.69502700	3.28087800
H	3.24097500	-2.50568100	1.72866400
C	1.50710700	-0.44456800	2.29517500
H	0.87993000	0.44203000	2.12580400

H	0.99201900	-1.32490900	1.88008000
H	1.56244100	-0.59794500	3.38468300
C	3.62810000	0.85586200	2.46874000
H	4.69848900	0.88792600	2.22931900
H	3.20159000	1.83327300	2.20049700
H	3.52746100	0.72119300	3.55668700
C	1.26415200	0.59558800	-3.14536000
H	0.51925500	-0.21604900	-3.09838000
H	0.77191800	1.54669100	-2.91400900
H	1.61852500	0.65258900	-4.18611500
C	3.28874400	1.66484300	-2.31394700
H	2.75688500	2.45899700	-1.76401000
H	4.30769700	1.57865700	-1.92730200
H	3.35781300	1.97070400	-3.36996700
C	3.25469400	-0.83404300	-2.89676500
H	4.11319300	-0.46509300	-3.47378100
H	3.63325100	-1.57270900	-2.17689800
H	2.60341800	-1.37757600	-3.59957200

122

INT2-Z-tBu E(MN15(SMD)/cc-pVTZ(pp))=-3319.92563956

C	-2.99493800	-2.54001100	-1.95261000
H	-3.78180300	-2.60665100	-1.20255000
C	-3.07503200	-3.33817700	-3.08674600
H	-3.91336200	-4.02421300	-3.21355100
C	-2.07810200	-3.24397900	-4.06072600
H	-2.12840700	-3.84968900	-4.96663800
C	-1.00730200	-2.37904200	-3.86948400
H	-0.23258700	-2.32648300	-4.63558100
C	-0.91230200	-1.59217200	-2.71057100
C	0.33429900	0.24262100	-3.65498100
H	0.40278200	-0.27594200	-4.62417500
H	1.22799300	0.86417100	-3.51859000
H	-0.56625700	0.87020700	-3.64254700
C	1.49032200	-1.58767100	-2.62329500
H	1.50156600	-2.28565000	-1.77844700
H	2.36931000	-0.93474000	-2.58533300
H	1.50412200	-2.14824000	-3.56964700
C	-2.09535600	-1.66957800	1.22724300
C	-1.31893500	-2.97968800	0.97418200
H	-0.28614900	-2.76100700	0.66260000
H	-1.80192700	-3.54279400	0.15842400
C	-1.33137700	-3.84564100	2.24274000
H	-0.74993500	-4.75949500	2.04293900
C	-1.47406400	-0.92635700	2.42995200
H	-0.42891300	-0.67292600	2.20258100
H	-2.01063900	0.02195200	2.61115200
C	-3.57916200	-2.93386600	2.82446500

H	-4.62929900	-3.18569800	3.04080600
C	-2.97567100	-2.16825900	4.00438500
H	-3.02179400	-2.78749000	4.91539400
H	-3.55857600	-1.25165100	4.20074100
C	-1.52399100	-1.81080500	3.68056900
H	-1.07884300	-1.25110400	4.51774400
C	-3.55231500	-2.04948100	1.56642400
H	-4.16645900	-1.15561600	1.74900700
H	-4.01625700	-2.60739600	0.73694400
C	-0.71492000	-3.08507600	3.42045900
H	-0.71998700	-3.72519300	4.31850300
H	0.33190400	-2.82569300	3.19812800
C	-2.77958300	-4.21498600	2.57483500
H	-3.22708300	-4.78694700	1.74345200
H	-2.81204500	-4.85787600	3.46991600
C	-3.28300200	0.65969300	-0.55010300
C	-4.09270800	3.49341700	-0.59573400
H	-4.82134600	4.30113200	-0.77430200
H	-3.16217600	3.97364600	-0.24357000
C	-5.64569500	1.11635300	-1.31608300
H	-6.56624500	0.62571400	-1.66866600
C	-3.61071900	1.42127900	0.75070700
H	-4.04552800	0.72829800	1.48712100
H	-2.70566500	1.83519600	1.21180600
C	-3.83482100	2.72820300	-1.89779700
H	-3.44390300	3.41515400	-2.66474800
C	-4.58928300	0.02482000	-1.06971800
H	-4.40342400	-0.49368800	-2.02342700
H	-4.97842600	-0.71247600	-0.34896400
C	-4.63211700	2.53110700	0.46739400
H	-4.81777300	3.07875900	1.40424600
C	-5.13420100	2.08379700	-2.38617600
H	-4.95800600	1.54612400	-3.33356300
H	-5.89018800	2.86098900	-2.58527100
C	-2.79101400	1.63446600	-1.64118300
H	-1.83005000	2.09384300	-1.35301300
H	-2.61331300	1.06047300	-2.57140100
C	-5.93009500	1.88786900	-0.02571400
H	-6.69422100	2.66046700	-0.21174100
H	-6.33133200	1.20919800	0.74630200
Au	0.34832900	0.45014400	-0.60276000
N	0.27518700	-0.73738600	-2.54241500
P	-1.86590000	-0.56027200	-0.27775800
C	0.42226900	1.63683000	1.06771300
C	1.38012900	1.48340300	2.07032400
C	-0.48715900	2.69674600	1.19853900
C	1.41100200	2.32596800	3.18827800

C	-0.46370300	3.54826800	2.29833600
C	0.48497600	3.36742200	3.31513300
H	2.17818500	2.15754000	3.94395100
H	-1.17631600	4.37006500	2.38969200
H	-1.22899000	2.89181800	0.42584800
C	2.21266200	1.34108800	-1.01193700
C	3.34904500	0.64154500	-0.76292700
H	2.14727400	0.71669100	2.02333400
O	0.43402200	4.23296000	4.34876100
C	1.38631800	4.09135200	5.38269100
H	1.30149200	3.10782700	5.87457800
H	2.41377400	4.21607000	5.00169000
H	1.17407500	4.87978800	6.11283800
C	4.86483800	0.78471800	-1.04780200
C	2.16174800	2.78673300	-1.56399000
C	-1.92491400	-1.64492600	-1.74800700
O	3.13922900	-0.76905500	-0.42050300
S	2.94811100	-1.45919200	1.01142900
O	3.80661800	-0.92223200	2.05459800
O	1.51829000	-1.64787200	1.25798600
C	3.57487700	-3.14039600	0.49472800
F	4.68374400	-3.04735200	-0.21603100
F	2.63461400	-3.73371500	-0.22759300
F	3.79341500	-3.82350400	1.59841400
C	0.73766100	3.28794300	-1.86753800
H	0.19640400	2.62860400	-2.56104800
H	0.14259300	3.42320500	-0.95892400
H	0.82297400	4.27280300	-2.35341200
C	5.70381800	0.13505900	0.06924000
H	5.49104700	0.58163700	1.04929800
H	5.56223000	-0.94852400	0.13362700
H	6.76440800	0.30926200	-0.16600600
C	5.45919200	2.19905800	-1.15190900
H	6.49389300	2.08336400	-1.50932800
H	4.95677200	2.88986900	-1.83116100
H	5.51731300	2.66638000	-0.15808600
C	5.14225300	-0.01725500	-2.33681700
H	4.81213200	-1.06039800	-2.21163900
H	4.63657200	0.40568100	-3.21850900
H	6.22479200	-0.02977500	-2.53736700
C	2.88575200	2.89551700	-2.93375100
H	3.66830300	2.14704400	-3.08867100
H	2.16165700	2.78013700	-3.75586800
H	3.33558000	3.89432900	-3.05026200
C	2.69779300	3.76701900	-0.49858800
H	2.01804300	3.77952600	0.36777900
H	3.69399200	3.50788600	-0.13137700

H	2.73041800	4.78510500	-0.91936000

122

TS2-E-tBu E(MN15(SMD)/cc-pVTZ(pp))=-3319.89972535 imaginary frequency= -341.7490

C	-3.64042500	-2.89967100	1.30979100
H	-4.47848700	-2.22755700	1.49667800
C	-3.75512800	-4.23850400	1.66935900
H	-4.67350800	-4.60275800	2.13162800
C	-2.68694700	-5.10596800	1.43087300
H	-2.75956900	-6.16070800	1.70061700
C	-1.52267700	-4.62156100	0.84271400
H	-0.69616100	-5.30813000	0.65039100
C	-1.39625400	-3.27049700	0.48778100
C	-0.08417800	-3.32002100	-1.52769600
H	-0.14142200	-4.42303000	-1.55658700
H	0.87316500	-3.00240200	-1.95890300
H	-0.90376100	-2.90149500	-2.12332900
C	1.03593200	-3.30040700	0.58527000
H	0.93242800	-3.08280900	1.65694800
H	1.91452400	-2.77085200	0.18586400
H	1.19829400	-4.38397500	0.45317500
C	-2.76766800	0.39364900	1.82029100
C	-2.13265200	-0.30767900	3.03834500
H	-1.06095700	-0.48826800	2.84640100
H	-2.60514700	-1.29151900	3.19385400
C	-2.31223300	0.54576000	4.30072400
H	-1.84135900	0.02132500	5.14692600
C	-2.10597000	1.78231500	1.65592100
H	-1.02751000	1.65557900	1.46538100
H	-2.51819800	2.31135400	0.78100300
C	-4.45488500	1.43422400	3.37675700
H	-5.53467200	1.55464300	3.55566900
C	-3.80748400	2.80728300	3.17911300
H	-3.96461800	3.42985100	4.07528300
H	-4.27911400	3.33055200	2.32942800
C	-2.30996100	2.62582500	2.91918600
H	-1.83414500	3.60681400	2.76118300
C	-4.26926800	0.58996400	2.10539600
H	-4.76375000	1.09879100	1.26326900
H	-4.77003500	-0.38182500	2.24629400
C	-1.65744000	1.91628400	4.10841800
H	-1.77555000	2.52025900	5.02326800
H	-0.57386700	1.79891500	3.93082800
C	-3.80893400	0.72494000	4.56984200
H	-4.28502100	-0.25727300	4.73212600
H	-3.96044600	1.31771600	5.48690700
C	-3.49212200	-0.32693200	-1.14631800
C	-3.76574400	0.76826200	-3.87616200

H	-4.33533600	0.97337200	-4.79723000
H	-2.75201200	1.18568000	-4.02032100
C	-5.74358200	-0.64811300	-2.23048000
H	-6.74860000	-1.06883600	-2.07174900
C	-3.61655200	1.18544800	-1.40996000
H	-4.11460200	1.67725400	-0.55940500
H	-2.61706000	1.63834700	-1.50468800
C	-3.68383700	-0.74152600	-3.63512000
H	-3.19237500	-1.23170000	-4.49003200
C	-4.89998900	-0.92857500	-0.97487000
H	-4.82433000	-2.01969300	-0.83643500
H	-5.40304600	-0.50975400	-0.08894500
C	-4.44224000	1.43710000	-2.67693400
H	-4.50541500	2.52397300	-2.84268500
C	-5.09023500	-1.31526600	-3.44323000
H	-5.03662400	-2.40720100	-3.29455900
H	-5.69737800	-1.13643000	-4.34580100
C	-2.85441900	-1.00769700	-2.37355300
H	-1.81974500	-0.64253500	-2.51749700
H	-2.80631200	-2.09602700	-2.18651000
C	-5.84605000	0.85997400	-2.47418200
H	-6.46979000	1.05449600	-3.36204900
H	-6.33518100	1.35339400	-1.61655500
Au	0.03021700	-0.39138400	-0.27125800
N	-0.15700100	-2.82937400	-0.13824700
P	-2.31893800	-0.61707100	0.29445100
C	0.77872000	1.56630100	-0.74772600
C	1.33068200	2.55896500	0.07564300
C	-0.08932300	2.01392100	-1.77369400
C	1.04004300	3.91297400	-0.08706100
C	-0.38601800	3.35832400	-1.94719800
C	0.17205400	4.33237700	-1.10494500
H	1.52887900	4.63322100	0.56895300
H	-1.07237100	3.68013100	-2.73301700
H	-0.59150900	1.30101900	-2.42548200
C	2.03312900	0.08969400	-0.81553600
C	2.95037400	-0.00356000	0.21448500
H	2.05566600	2.28951300	0.83570300
O	-0.16518500	5.60956300	-1.35155800
C	0.38320300	6.62079800	-0.52770000
H	0.09206300	6.47809000	0.52626400
H	1.48280700	6.64271700	-0.60158900
H	-0.02237800	7.57113800	-0.89025400
C	2.88007600	0.01849700	1.75199200
C	2.47922900	-0.11618100	-2.29366400
C	-2.46934600	-2.38750100	0.71717900
O	4.31554800	0.11949800	-0.18331400

S	5.53815800	-0.92339800	-0.45668500
O	5.24926900	-2.24809700	0.07496400
O	6.01795400	-0.70514400	-1.80968600
C	6.81071700	-0.06525400	0.60554500
F	6.61874800	-0.32457400	1.88946500
F	6.73016600	1.23739900	0.39769100
F	7.98792300	-0.51696200	0.22889200
C	3.53933300	-1.26264800	2.31250900
H	4.62521900	-1.25696100	2.19616600
H	3.32448000	-1.30949400	3.39145400
H	3.15899800	-2.17489300	1.83758700
C	1.47099800	0.05007300	2.33763000
H	0.87311400	0.91277000	2.01321800
H	0.92109200	-0.87745400	2.10477400
H	1.55501500	0.10317600	3.43503000
C	3.67719200	1.20555900	2.34255800
H	4.62360500	1.37158400	1.81448400
H	3.11109800	2.14823800	2.34361000
H	3.91031700	0.97719000	3.39358600
C	1.32582400	-0.14625600	-3.31436200
H	0.46496500	-0.74741800	-2.96810000
H	0.98570400	0.85622900	-3.59770200
H	1.70842900	-0.61833100	-4.23193600
C	3.38734600	1.04292200	-2.75523400
H	2.87386400	2.00437600	-2.58943600
H	4.36105800	1.06621400	-2.25886600
H	3.56548300	0.93776400	-3.83709000
C	3.16044400	-1.50270300	-2.44659600
H	4.01715600	-1.43824900	-3.13130000
H	3.50872500	-1.94462000	-1.50481900
H	2.45595200	-2.22688800	-2.88206700

122

TS2-Z-tBu E(MN15(SMD)/cc-pVTZ(pp))=-3319.88899024 imaginary frequency= -332.9614

C	3.28720500	2.15415000	-2.11745500
H	4.03475300	2.24705800	-1.33044400
C	3.47839500	2.84325800	-3.31010900
H	4.36199600	3.46915100	-3.44192900
C	2.53505500	2.72042300	-4.33259800
H	2.67123300	3.24597800	-5.27903700
C	1.41355900	1.92149400	-4.13881200
H	0.68027700	1.82746500	-4.94230400
C	1.20862600	1.23643800	-2.93172200
C	0.02586100	-0.71295900	-3.72204900
H	0.07126800	-0.37588300	-4.77322000
H	-0.89669600	-1.29096400	-3.57642600
H	0.89278800	-1.35324600	-3.51350800
C	-1.20837000	1.22518700	-3.04932400

H	-1.23116200	2.08808800	-2.37317100
H	-2.08245500	0.58770400	-2.85988900
H	-1.25331900	1.57505100	-4.09540200
C	2.14176000	1.66641100	1.08781300
C	1.39662300	2.95693200	0.68865900
H	0.36151400	2.71532600	0.39377500
H	1.89348100	3.41919900	-0.18099100
C	1.40572700	3.95267500	1.85769600
H	0.86225200	4.85893600	1.54651000
C	1.45688700	1.05846400	2.33554100
H	0.41783900	0.78441300	2.09507600
H	1.97725800	0.13703900	2.64503200
C	3.60299300	3.03996800	2.61455100
H	4.65092600	3.28493200	2.84902800
C	2.93073200	2.41370800	3.83886700
H	2.96067100	3.11877600	4.68607500
H	3.47800000	1.50586600	4.14764600
C	1.48095800	2.06661100	3.49296500
H	0.98808700	1.60523900	4.36309700
C	3.59390200	2.03379500	1.45314300
H	4.16101200	1.13847200	1.75242900
H	4.11471100	2.48524700	0.59366700
C	0.73161300	3.33904400	3.08806600
H	0.75015400	4.06354200	3.91979200
H	-0.32035900	3.10513800	2.87034800
C	2.85533900	4.30960400	2.19841100
H	3.35008900	4.77211100	1.32663000
H	2.88001100	5.04628300	3.01839500
C	3.24515100	-0.90406800	-0.33424800
C	3.78438600	-3.78868400	0.01080900
H	4.44493500	-4.67109800	-0.00008100
H	2.77898600	-4.13803600	0.31153500
C	5.61267900	-1.63883700	-0.79745500
H	6.60653000	-1.27456900	-1.10065400
C	3.35667200	-1.54917400	1.06060300
H	3.75042800	-0.81843800	1.78412900
H	2.36248600	-1.85252500	1.42516800
C	3.72397100	-3.15845100	-1.38332300
H	3.34388200	-3.89345900	-2.11044600
C	4.64518100	-0.44304300	-0.78282300
H	4.59111600	-0.02361700	-1.80048800
H	5.03408800	0.34154500	-0.11429200
C	4.30388900	-2.75443500	1.01315500
H	4.35170900	-3.20119900	2.01853900
C	5.11696600	-2.68209600	-1.80185800
H	5.07980400	-2.24731700	-2.81529000
H	5.81393100	-3.53564300	-1.83611700

C	2.76615900	-1.96296200	-1.34617300
H	1.75308500	-2.30846600	-1.07397100
H	2.70662000	-1.49663700	-2.34742800
C	5.69522600	-2.27153800	0.59401500
H	6.40346700	-3.11631500	0.58084300
H	6.07466900	-1.53771700	1.32578000
Au	-0.31692400	-0.48130300	-0.54984400
N	0.00369900	0.42883700	-2.79338100
P	1.92383500	0.44160200	-0.32187000
C	-1.26985500	-1.57345800	1.05360000
C	-2.26332200	-1.24254400	1.98966900
C	-0.23084700	-2.41441100	1.52705100
C	-2.22194900	-1.67735000	3.31373900
C	-0.17772800	-2.85798700	2.83934000
C	-1.17159100	-2.48919300	3.76044600
H	-3.04091400	-1.39579900	3.97523100
H	0.64543100	-3.48707400	3.18288000
H	0.58913200	-2.69982100	0.87059500
C	-2.18391000	-1.54936400	-0.66573100
C	-3.23051900	-0.66087200	-0.77218700
H	-3.15721600	-0.69595100	1.69589200
O	-1.04413700	-2.96444700	5.00983600
C	-2.02079900	-2.60327300	5.96889800
H	-2.06375700	-1.50972400	6.10076000
H	-3.01794000	-2.97468200	5.68118300
H	-1.71851400	-3.07173800	6.91134100
C	-4.69034200	-0.69649600	-1.27627800
C	-2.18181100	-3.00155100	-1.21477200
C	2.15836300	1.33896100	-1.89960600
O	-2.89084600	0.72736300	-0.50475400
S	-2.89684200	1.66825200	0.82124800
O	-4.04682500	1.49216000	1.69316900
O	-1.54836400	1.71126300	1.37244200
C	-3.11986800	3.25866800	-0.12710700
F	-4.04354900	3.12919500	-1.06361600
F	-1.96386100	3.59131100	-0.68407900
F	-3.47946500	4.17632400	0.74469400
C	-0.76528100	-3.58453100	-1.37141700
H	-0.12409400	-2.93321200	-1.98536200
H	-0.27267500	-3.79890500	-0.41750500
H	-0.86140600	-4.54447900	-1.90222900
C	-5.58888800	0.15656900	-0.35723800
H	-5.53907200	-0.16932900	0.69081300
H	-5.35655600	1.22518300	-0.41080400
H	-6.62577000	0.03135900	-0.70250300
C	-5.39540000	-2.06188700	-1.27959700
H	-6.38765900	-1.91114900	-1.73099600

H	-4.90406200	-2.85923900	-1.83937500
H	-5.56276500	-2.40644200	-0.24814600
C	-4.75678700	-0.02390500	-2.66274600
H	-4.34690700	0.99586000	-2.60223500
H	-4.21380900	-0.57325500	-3.44585000
H	-5.80968400	0.05560100	-2.97435300
C	-2.74719100	-3.06986500	-2.66057100
H	-3.37060300	-2.21694100	-2.93836000
H	-1.92373600	-3.11848500	-3.38821700
H	-3.33909400	-3.98790200	-2.79780000
C	-2.91205500	-3.94469100	-0.23656600
H	-2.35221000	-4.01658100	0.70833400
H	-3.92851500	-3.61654500	0.00214000
H	-2.96556500	-4.95377000	-0.67460200

51

prod-E-tBu E(MN15(SMD)/cc-pVTZ(pp))=-1698.47948573

C	-1.95396300	0.46923700	0.24851500
C	-2.47464200	0.06405800	-0.98206100
C	-2.85296100	0.62497900	1.32036400
C	-3.83633200	-0.21566400	-1.15373200
C	-4.20313200	0.34909300	1.17077900
C	-4.70794100	-0.07945100	-0.06910600
H	-4.19682900	-0.53442400	-2.13169300
H	-4.90219500	0.44969900	2.00248500
H	-2.47594300	0.93339100	2.29765900
C	-0.48107300	0.72727000	0.37076100
C	0.32362600	-0.35578300	0.46052800
H	-1.79537800	-0.04461800	-1.83165800
O	-6.03976100	-0.32807400	-0.11730900
C	-6.58284500	-0.76832100	-1.34007900
H	-6.44421200	-0.01871000	-2.13817800
H	-6.12892300	-1.72052700	-1.66468700
H	-7.65527300	-0.91980800	-1.17176700
C	-0.00716600	-1.83993300	0.72802600
C	-0.08089400	2.21863500	0.27556800
O	1.74130400	-0.12754700	0.45020800
S	2.62011900	-0.30067900	-0.89326800
O	2.62720600	-1.67447600	-1.37962900
O	2.41368400	0.81006600	-1.81243000
C	4.21467300	-0.05468700	0.01874200
F	4.33984600	-1.00250700	0.93180500
F	4.24462100	1.13390700	0.59490100
F	5.19390100	-0.14941700	-0.86566100
C	1.19320700	-2.48974500	1.44492300
H	1.43732600	-1.95530700	2.37630500
H	0.92143300	-3.52452300	1.70192500
H	2.09095900	-2.52310600	0.81639200

C	-0.24976600	2.67666900	-1.18262200
H	-1.28961200	2.54422600	-1.52041800
H	-0.00431200	3.74895800	-1.25682300
H	0.42234200	2.11998300	-1.84985600
C	1.33604200	2.56437500	0.75649300
H	1.55819000	2.10962200	1.73346300
H	2.10878500	2.26774100	0.04229800
H	1.39838200	3.65864400	0.86670800
C	-1.03549600	3.06340000	1.14091500
H	-2.08101600	2.98909500	0.81305600
H	-0.97617400	2.78164100	2.20467800
H	-0.73217600	4.11821800	1.05622500
C	-0.29340000	-2.61850800	-0.56783200
H	-1.20962500	-2.24894500	-1.05047200
H	0.54091600	-2.56116600	-1.27761400
H	-0.45213800	-3.67952700	-0.31331900
C	-1.21690600	-2.02858300	1.66037600
H	-1.16588600	-1.36123600	2.53471600
H	-2.17600800	-1.86948100	1.15106200
H	-1.20551400	-3.06880500	2.02233400

51

prod-E-tBu E(MN15(SMD)/cc-pVTZ(pp))=-1698.473906

C	1.19784800	0.94096900	-0.04021600
C	1.86099600	0.71541300	1.16872300
C	1.84518100	0.56421200	-1.23053700
C	3.13034600	0.13256300	1.21044000
C	3.10555800	-0.01379300	-1.20502100
C	3.75834300	-0.23749000	0.01612200
H	3.61022100	-0.03427800	2.17473300
H	3.60860100	-0.32523900	-2.12160900
H	1.33082700	0.69205500	-2.18483500
C	-0.15961800	1.58215700	-0.03302500
C	-1.18804700	0.80300400	0.38477500
H	1.36489500	0.98891700	2.10364600
O	4.98441600	-0.81398900	-0.05804800
C	5.65288200	-1.09462700	1.14818200
H	5.85993700	-0.17429800	1.72190200
H	5.07024800	-1.78443900	1.78281100
H	6.60333800	-1.56982500	0.87865300
C	-2.68826500	0.97514300	0.67272700
C	-0.16738800	3.08354300	-0.41063200
O	-0.84459400	-0.56469600	0.66518000
S	-0.39586600	-1.60409800	-0.49377800
O	-0.70460100	-1.06056700	-1.81081300
O	0.87315500	-2.21058600	-0.13599900
C	-1.64720900	-2.92888500	-0.13912100
F	-2.84474400	-2.57763100	-0.59111800

F	-1.71415400	-3.15093500	1.16448300
F	-1.25197500	-4.02847200	-0.75845200
C	-3.13593800	-0.07891800	1.70850900
H	-4.17656700	0.14043600	1.99201300
H	-3.10346000	-1.10024600	1.31729700
H	-2.51425000	-0.03847500	2.61557800
C	1.12690000	3.48811000	-1.14454400
H	1.07485800	4.57022100	-1.34313800
H	2.02691700	3.29035700	-0.54630000
H	1.23564500	2.97541600	-2.11065900
C	-3.09114100	2.31195900	1.30998400
H	-2.60536300	2.43316000	2.29087800
H	-2.87806500	3.19988100	0.70876300
H	-4.17903800	2.28871000	1.47917600
C	-3.47136500	0.72118500	-0.62711500
H	-3.29466800	1.50547200	-1.37549600
H	-3.17114700	-0.23724500	-1.07303900
H	-4.55109400	0.68035400	-0.40876800
C	-1.29498500	3.45832700	-1.38322200
H	-1.20155500	2.86263800	-2.30520200
H	-2.30522700	3.30599100	-0.99342100
H	-1.20120200	4.52194200	-1.65551200
C	-0.16789700	3.92160300	0.88072000
H	0.72674600	3.67229800	1.47513900
H	-0.11601900	4.99387500	0.62985700
H	-1.04707500	3.75309400	1.51070200

References

S1) Bruker APEX3, SAINT and SADABS software, Bruker AXS Inc.: Madison, Wisconsin, USA, 2017.

S2) a: G. M. Sheldrick, Acta Cryst. 2015 A71, 3-8; b: G. M. Sheldrick, *Acta Cryst.* 2008 A64, 112-122 and 2015 C71, 3-8.

S3) Gaussian 16, Revision C.01, M. J. Frisch, G. W. Trucks, H. B. Schlegel, G. E. Scuseria, M. A. Robb, J. R. Cheeseman, G. Scalmani, V. Barone, G. A. Petersson, H. Nakatsuji, X. Li, M. Caricato, A. V. Marenich, J. Bloino, B. G. Janesko, R. Gomperts, B. Mennucci, H. P. Hratchian, J. V. Ortiz, A. F. Izmaylov, J. L. Sonnenberg, D. Williams-Young, F. Ding, F. Lipparini, F. Egidi, J. Goings, B. Peng, A. Petrone, T. Henderson, D. Ranasinghe, V. G. Zakrzewski, J. Gao, N. Rega, G. Zheng, W. Liang, M. Hada, M. Ehara, K. Toyota, R. Fukuda, J. Hasegawa, M. Ishida, T. Nakajima, Y. Honda, O. Kitao, H. Nakai, T. Vreven, K. Throssell, J. A. Montgomery, Jr., J. E. Peralta, F. Ogliaro, M. J. Bearpark, J. J. Heyd, E. N. Brothers, K. N. Kudin, V. N. Staroverov, T. A. Keith, R. Kobayashi, J. Normand, K. Raghavachari, A. P. Rendell, J. C. Burant, S. S. Iyengar, J. Tomasi, M. Cossi, J. M. Millam, M. Klene, C. Adamo, R. Cammi, J. W. Ochterski, R. L. Martin, K. Morokuma, O. Farkas, J. B. Foresman, and D. J. Fox, Gaussian, Inc., Wallingford CT, 2016

S4) H. Y. S. Yu, X. He, S. H. L. Li, D. G. Truhlar, *Chem. Sci.* 2016, **7**, 5032-5051.

S5) D. Figgen, K. A. Peterson, M. Dolg, H. Stoll, *J. Chem. Phys.* 2009, **130**, 164108.

S6) B. P. Pritchard, D. Altarawy, B. Didier, T. D. Gibson, T. L. Windus, *J. Chem. Inf. Model.* 2019, **59**, 4814-4820.

S7) B. Foresman, T. A. Keith, K. B. Wiberg, J. Snoonian, M. J. Frisch, *J. Phys. Chem.* 1996, **100**, 16098-16104;

S8) V. Marenich, C. J. Cramer, D. G. Truhlar, *J. Phys. Chem. B* 2009, **113**, 6378-6396.

S9) F. Weigend, R. Ahlrichs, *Phys. Chem. Chem. Phys.* 2005, ***7***, 3297-3305.

S10) L. Falivene, Z. Cao, A. Petta, L. Serra, A. Poater, R. Oliva, V. Scarano, L. Cavallo, *Nat. Chem.* 2019, **11**, 872.
